# Discovery of a novel ligand that modulates the protein–protein interactions of the AAA+ superfamily oncoprotein reptin†
†Electronic supplementary information (ESI) available. See DOI: 10.1039/c4sc03885a



**DOI:** 10.1039/c4sc03885a

**Published:** 2015-03-20

**Authors:** Alan R. Healy, Douglas R. Houston, Lucy Remnant, Anne-Sophie Huart, Veronika Brychtova, Magda M. Maslon, Olivia Meers, Petr Muller, Adam Krejci, Elizabeth A. Blackburn, Borek Vojtesek, Lenka Hernychova, Malcolm D. Walkinshaw, Nicholas J. Westwood, Ted R. Hupp

**Affiliations:** a School of Chemistry & Biomedical Sciences Research Complex , University of St Andrews & EaStCHEM , North Haugh, St Andrews , KY16 9ST , UK . Email: njw3@st-andrews.ac.uk; b Centre for Chemical Biology , University of Edinburgh , EH9 3JG , UK . Email: DouglasR.Houston@ed.ac.uk; c Edinburgh Cancer Research Centre , Cell Signalling Unit , University of Edinburgh , EH4 2XR , UK . Email: ted.hupp@ed.ac.uk; d RECAMO , Masaryk Memorial Cancer Institute , 656 53 Brno , Czech Republic

## Abstract

Developing approaches to discover protein–protein interactions (PPIs) remains a fundamental challenge. A chemical biology platform is applied here to identify novel PPIs for the AAA+ superfamily oncoprotein reptin. An *in silico* screen coupled with chemical optimization provided Liddean, a nucleotide-mimetic which modulates reptin's oligomerization status, protein-binding activity and global conformation. Combinatorial peptide phage library screening of Liddean-bound reptin with next generation sequencing identified interaction motifs including a novel reptin docking site on the p53 tumor suppressor protein. Proximity ligation assays demonstrated that endogenous reptin forms a predominantly cytoplasmic complex with its paralog pontin in cancer cells and Liddean promotes a shift of this complex to the nucleus. An emerging view of PPIs in higher eukaryotes is that they occur through a striking diversity of linear peptide motifs. The discovery of a compound that alters reptin's protein interaction landscape potentially leads to novel avenues for therapeutic development.

## Abstract 2

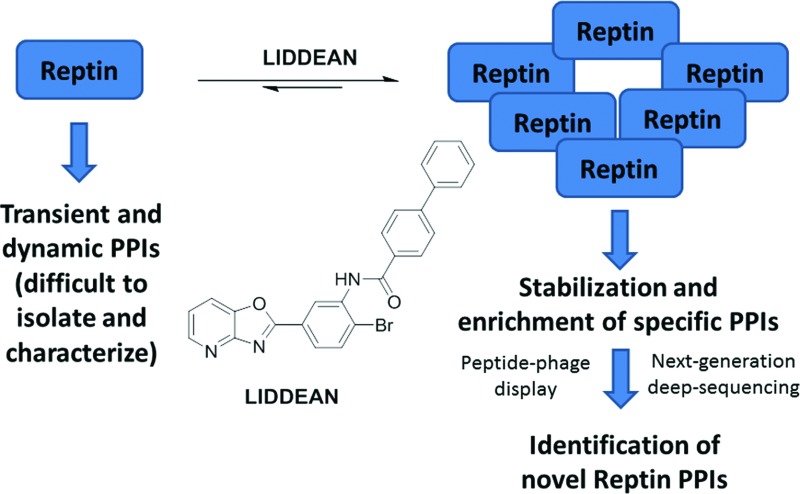
Discovery and use of a chemical tool.

## Introduction

Discovering protein–protein interactions (PPIs) remains a major challenge.^1^ However, a detailed understanding of a protein's PPI network is central to novel biomedical applications. Rate-limiting protein nodes need to be identified as they can serve as a focus for novel diagnostic and/or therapeutic advances. The current view is that drugging PPIs remains an untapped landscape in the drug discovery field.

The main approaches used to define the PPIs of a target protein include yeast two-hybrid methods^2^ and mass-spectrometry-based sequencing of multi-protein complexes using tagged-bait proteins.^3^ Although powerful, their limitations are that they are done outside an authentic cellular context, require artificial tagging of the bait protein and are unable to capture weak, or dynamic interactions. One advance in the study of PPIs is the idea that a large proportion of the polypeptide sequence information in higher eukaryotes is intrinsically disordered thus providing a template for “weak” regulatory, combinatorial and specific PPIs to occur in signal transduction.^4^ A second advance is the realization that a number of PPIs occur *via* a linear amino acid motif^1^ that provides opportunities for sequence based hotspots to be identified. Developing methods to capture such consensus linear motifs of a target protein would complement the technology currently used to discover PPIs.

The AAA+ (ATPase associated with various cellular activities) superfamily of proteins is present within all kingdoms of life.^5^ Members of this family exist as oligomers and form compelling targets in understanding allosteric control of protein function.^6^ Reptin and pontin represent two highly conserved members that are now viewed as model systems to define fundamental aspects of AAA+ superfamily function in eukaryotes.^7^ Reptin is an important regulator of key cellular functions through a range of PPIs.^8–19^ The different oligomeric forms of reptin and its ability to form a range of complexes with different compositions could underpin its functional diversity.^20–22^ The composition of these oligomeric complexes must be tightly regulated and this has been linked to reptin's bound ligand ATP/ADP.^23,24^ Development of a synthetic ATP/ADP mimetic to probe the intrinsic oligomerization properties of reptin and its ability to form diverse PPIs could provide insight into the regulation and function of reptin and the wider AAA+ family.

Here, a chemical biology platform is used to deliver a novel tool to dissect the function of reptin. This approach involves; (i) *in silico* screening of virtual libraries to identify novel ATP mimetics; (ii) optimization of a hit as a PPI and oligomerization modifier giving the novel chemical tool, Liddean; (iii) use of combinatorial-peptide libraries and next generation sequencing to identify novel responsive PPIs; and (iv) cell based validation of ligand-activated PPIs using proximity ligation assays. We also demonstrate that our chemical tool Liddean (an ATP-mimetic) can be used to discover and manipulate the PPIs of the AAA+ protein reptin.

## Results

### *In silico* screening leading to a prioritized ATP mimetic

**Fig. 1:**
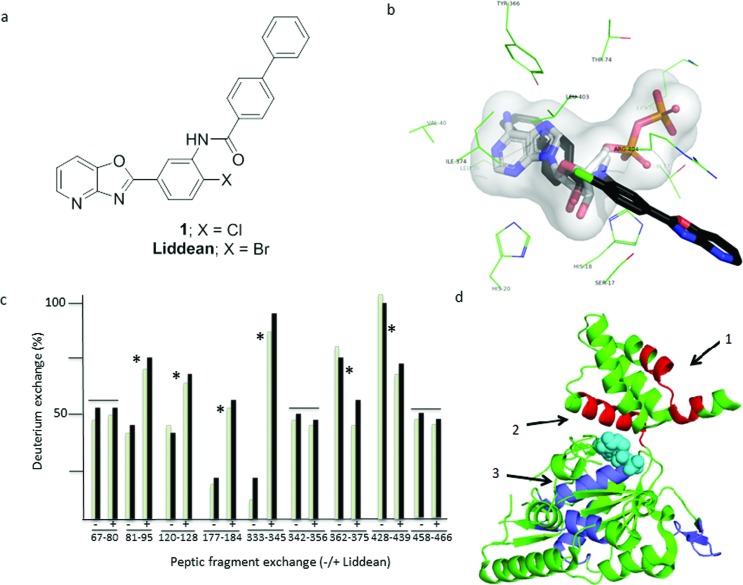
Identification of small molecules targeting reptin using an *in silico* screening programme. (a) Structure of hit compound **1** was identified through a reptin–AGR2 peptide interaction assay of the top 30 hits from the *in silico* screen (see Fig. S1 and S2†) and Liddean, the most active analog obtained through chemical optimization (see Scheme S1 and Fig. S4 and S5†). (b) The Autodock prediction of the binding mode of **1** is shown as black sticks. The side chains of residues that comprise the active site are shown as green lines and are labeled. The ADP molecule is colored white and shown as sticks. The pocket is also shown as a transparent surface representation. In all cases nitrogen is colored blue, oxygen red, phosphorous orange, and chlorine green. See also Fig. S1 and S3.† (c) Changes in hydrogen–deuterium exchange on reptin peptide motifs after pepsin proteolysis. Ligand free reptin protein (Fig. S7a†) was digested with pepsin after processing in the absence or presence of Liddean. Processing of peptides was performed using HPLC-MS/MS with the instrument operated in a data-dependent mode. All identified peptides are shown as green bars (1 minute) or black bars (five minutes) without or with Liddean (L) and the % change in deuteration as a function of peptide fragment is highlighted. Shown are representative peptide fragments with increased or decreased deuterium exchange with ligand (*) or without changes in deuterium exchange with ligand (–). The peptic peptides cover the majority of the sequence of reptin with the numbering shown below the amino acid sequence. The sequence includes six residual N-terminal amino acids from the “tag” after precision protease cleavage from glutathione beads, amino acids and includes GPLGST (Fig. S7a and b†). (d) The reptin protein (PDB 3UK6) is displayed as a cartoon in green. The ADP molecule from reptin is shown as spheres in cyan. Regions with suppressed deuterium exchange are shown in red and regions with increased deuterium exchange are shown in blue. Key regions which form a dimer interface (R428-S439, arrow 1) and the ATP pocket (T81-G95 (arrow 2) & Y362-C375 (arrow 3)) are highlighted. The most dominant peptide fragments which show alterations in deuterium exchange ((c) and Table S2†) map either around the ADP binding site or at the dimer interface.

An *in silico* screen was used to identify small molecules that might bind at the Walker A site on reptin. The rigid-body docking program LIDAEUS^25^ was used to dock a conformer virtual library of 4.4 million compounds. The results were ranked based on the LIDAEUS score and the top 49 971 compounds were redocked using Vina and Autodock (Fig. S1†). A “rank-by-rank” consensus protocol prioritized hits, culminating in the selection of 30 compounds for assessment using an ELISA assay^17^ with the peptide 104-FVLLNLVY-111 from the known reptin binding protein AGR2^17^ (Table S1†). Hits from this assay were defined as compounds that modified (increased or decreased) significantly the signal corresponding to reptin binding to the AGR2-peptide compared to control. Of the 30 compounds tested (Fig. S2†), compound **1** (Fig. 1a) led to the most dramatic response and was therefore prioritized for study. Compound **1** contains a biphenyl substituent that is predicted to sit deep in the Walker A pocket where the adenine of ADP/ATP binds and a pyridine–oxazolo ring system which is predicted to extend out of the pocket (Fig. 1b and S3†).

### Structure activity relationship and hydrogen–deuterium exchange studies

The synthesis of **1** (Scheme S1†) provided sufficient material for hit validation studies. An SAR study was then carried out to improve the activity of **1**. Modified analogs were either purchased or synthesized (Schemes S1 and S2 and Fig. S4†). The bromo-analog of **1**, now called Liddean (Fig. 1a), was found to be the most active analog. The results from this SAR study (see Fig. S5† legend for a more detailed discussion), supported by hydrogen–deuterium exchange (HDX) studies (Fig. 1c and d),^26^ were consistent with the proposed binding mode. Importantly, suppression of HDX by Liddean was most pronounced for amino acids 362–375 in the Walker A site (Fig. S6†). Interestingly, an increased rate of HDX was observed along the length of the α-helix (81–95) that contacts to the Walker A site. In addition, deuterium exchange was suppressed for amino acids 428–439 which are located at the known protein–protein interface in reptin homodimers (Fig. 1c and d). It was therefore decided to assess whether Liddean had an effect on the oligomerization status of reptin.

### Liddean alters reptin's oligomerization status

**Fig. 2:**
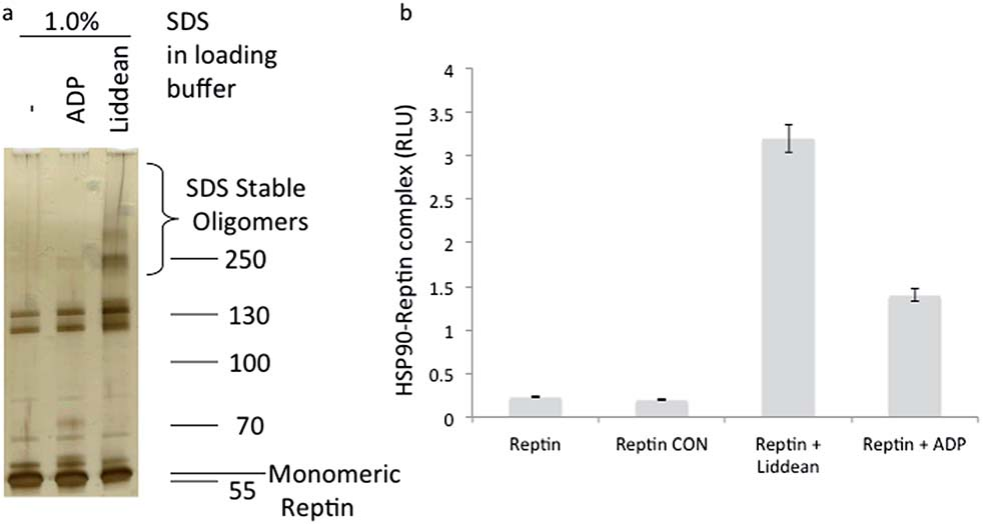
(a) The effect of Liddean on the oligomerization dynamics of reptin. (a) Reptin (1 µg) was subjected to denaturing SDS (0.1%) gel electrophoresis in ADP binding buffer without or with ADP (100 µM) or Liddean (100 µM), as indicated. After 30 minutes of incubation at room temperature, gel loading buffer was added (with 1% SDS concentration), and electrophoresis was then carried out. Reptin protein was visualized by silver staining. See Fig. S8† for additional data. (b) The effect of Liddean on the interaction of reptin with the molecular chaperone HSP90. SBP-tagged HSP90 was captured on streptavidin coated wells and reptin (100 ng) was added in the ligand free and ligand bound states (100 µM where indicated). After washing the amount of reptin bound was quantified using an anti-reptin polyclonal antibody and peroxidase conjugated-anti rabbit IgG. The data are plotted as relative reptin activity (in RLU) as a function of the ligand bound state of reptin. CON = control.

Oligomerization of reptin is known to increase in the presence of bound ligands ATP or ADP.^24^ Reptin was subjected to SDS denaturing (0.1%) electrophoresis in the presence of varying amounts of SDS (Fig. 2a and S8a†). The preincubation of reptin with Liddean induced the formation of a stable oligomeric form of reptin (MW ≈ 250 kDa, Fig. 2a) as well as additional bands, corresponding to higher order oligomers (possibly hexamers based on the apparent mass). These data suggested that Liddean was able to modify reptin's oligomerization dynamics with reptin being present in a more stable oligomeric state in the presence of Liddean. Liddean was also more effective at stabilizing reptin than ADP (Fig. 2a) with as little as 2 µM Liddean inducing stable oligomers (Fig. S8b,† lane 2). An attempt to correlate Liddean's ability to induce reptin homo-oligomerization and its stimulation of reptin's binding to the AGR2 peptide proved successful (Fig. S8†).

The link between nucleotide-induced changes in oligomerization status and function is a key feature of AAA+ proteins including reptin. To assess whether our Liddean-induced change in oligomerization status led to a modification of reptin's PPIs, we initially evaluated the effect of Liddean on reptin's known PPI with the molecular chaperone HSP90. Streptavidin-mimetic tagged (SBP) HSP90 was captured on solid phase followed by the addition of either apo-, ADP- or Liddean-bound reptin. Liddean (and to a lesser extent ADP) increased the stability of the reptin–HSP90 complex (Fig. 2b). Encouraged by the fact that clear changes could be observed in the presence of Liddean, we next used it as a tool to examine Liddean-induced changes on the global peptide-binding space of reptin.

### Discovering new Liddean-stimulated peptide docking motifs on reptin using next generation sequencing of a phage-peptide combinatorial library

**Fig. 3:**
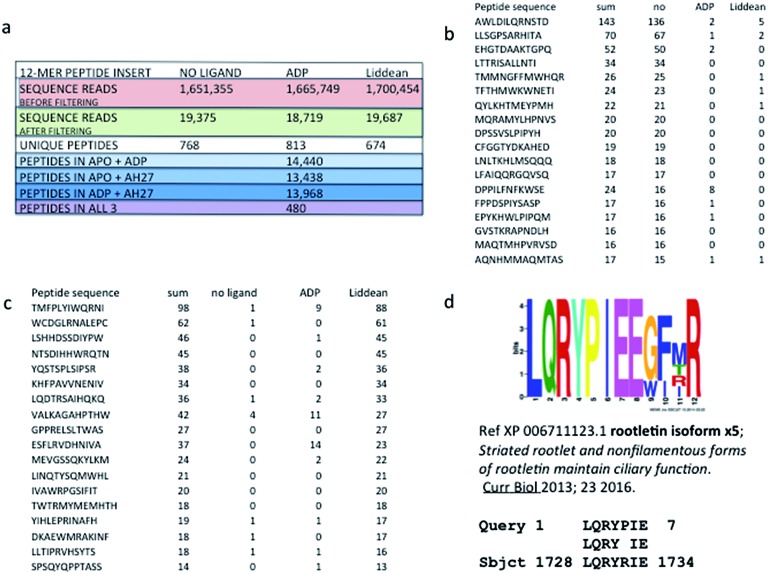
Discovery of new Liddean-dependent interaction motifs for reptin. Next generation sequencing of peptide-phage pool obtained from a reptin screen in the *apo* and ligand bound state. Reptin was captured onto the solid phase without or with ligands ADP or Liddean. After selection of the peptide library on reptin protein, elution and propagation in bacteria, the phage DNA was amplified using PCR primer sets that capture the sequences flanking the peptide insert (as in Fig. S9†). Pooling of all phage into deep sequencing reactions can be done with subsequent deconvolution using the “bar code” whose position in the primer is indicated. (a) Parameters from the sequencing reactions from a representative screen are summarized. These include: (i) the sequencing reads before filtering non-specific binding peptides; (ii) the number of sequencing reads in *apo* or ligand bound protein; (iii) and the number of peptides that are shared in a number of *apo* or ligand bound screens. (b and c) Representative peptides that are enriched in the ligand bound state or suppressed in the ligand bound state are indicated to highlight a representative set of raw sequencing reads. (d) An example ciliopathy protein present in the list of human proteins which contain consensus sites identified by our Liddean-bound reptin screen. Processing the top 500 peptides from the *apo* and Liddean bound reptin using MEME to identify the top 10 consensus motifs (; http://meme.nbcr.net/meme/cgi-bin/meme.cgi) highlights the distinct sets of motifs acquired in the *apo* and ligand bound form. The motifs were processed using MAST or *blastp* to identify targets in the human proteome that have matches to these motifs, some of which are listed as potential ciliopathy targets.

A combinatorial peptide-based selection assay exploiting next-generation “deep” DNA sequencing of peptide-phage pools was carried out using reptin in its ligand-free, ADP- or Liddean-bound form. Peptides were processed through 3 rounds of biopanning using a 12-*mer* peptide library, and peptide pools were sequenced (Fig. 3a, S9 and Table S3†). Comparison of the peptides identified using apo-reptin with those obtained when Liddean-bound reptin was used showed that, as expected, the binding of some peptides to reptin were suppressed (for representative raw peptide reads see Fig. 3b) or elevated by Liddean (Fig. 3c). Recent data has shown that reptin has an important interaction with the cytosolic cilia machinery.^27,28^ This is a new cellular interactome for reptin distinct from its known links to the chaperone and transcription systems. It is interesting to note that several ciliopathy proteins are present in the list of human proteins which contain consensus sites identified by our Liddean-bound reptin screen (Fig. 3d for one example, and Fig. S10†). Whilst the identified binding sites have yet to be validated, these data are entirely consistent with this approach being able to deliver a molecular peptide-binding “fingerprint” for reptin. In addition, the change in binding motifs identified in the presence of Liddean, linked with this ligand's observed effect on the oligomerization of reptin, provides indirect evidence for a substantial allosteric effect on the peptide-binding profile of reptin.

**Fig. 4:**
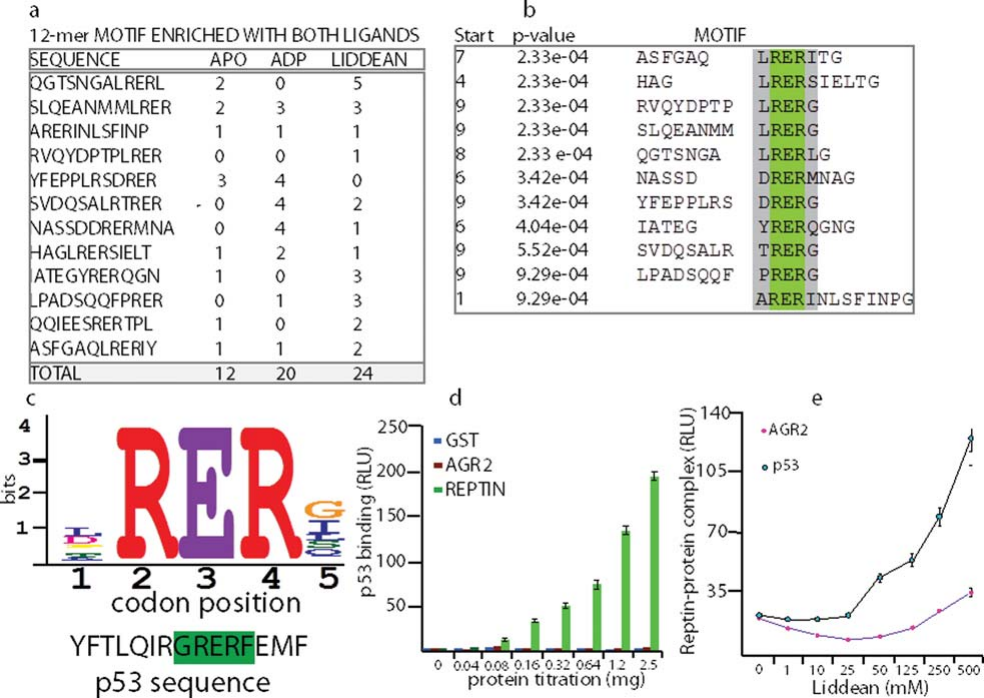
Identification and validation of p53 as a novel reptin interacting protein. (a) 1000 peptides that were enriched in the ligand bound state (including those shown) were processed using MEME to identify consensus motifs (http://meme.nbcr.net/meme/cgi-bin/meme.cgi). (b) The panel represents data from a 12-*mer* peptide screen where the core motif identified is highlighted as φRERφ or LRER[L/G]. (c) A blast motif screen using the MEME derived peptide consensus sites gave rise to a peptide derived from the tumor suppressor protein p53. (d) GST-tagged reptin and AGR2 proteins were assayed for their ability to bind to full length p53 using ELISA. p53 protein was absorbed onto the solid phase, and the indicated proteins were titrated in solution phase. The binding was detected using GST antibodies, followed by peroxidase conjugated secondary antibodies and processing using chemiluminescence. The data is plotted as binding activity as a function of protein amount (in RLU). (e) The effect of Liddean on the stability of the reptin–AGR2 and reptin–p53 protein interactions was evaluated. Either p53 or AGR2 were absorbed onto the solid phase and reptin (200 ng) was added in 50 µl of buffer containing increasing amounts of the indicated ligand. After 60 minutes incubation at room temperature, reptin protein bound to its target was quantified as indicated in the methods (in RLU).

During these studies, several consensus peptide motifs were identified that were enriched in both ADP and Liddean bound forms. Our attention was drawn to the enrichment of motifs containing a φRERφ sequence by ADP and/or Liddean (Fig. 4a and b). When this motif was compared with the human proteome, a motif was identified in the tetramerization domain of the p53 tumor suppressor (Fig. 4c). An ELISA assay demonstrated that the reptin protein was able to bind to purified human p53 whereas GST and AGR2 did not (Fig. 4d). Liddean was shown to stabilize the p53–reptin complex, consistent with the results from peptide-phage display (Fig. 4e).

**Fig. 5:**
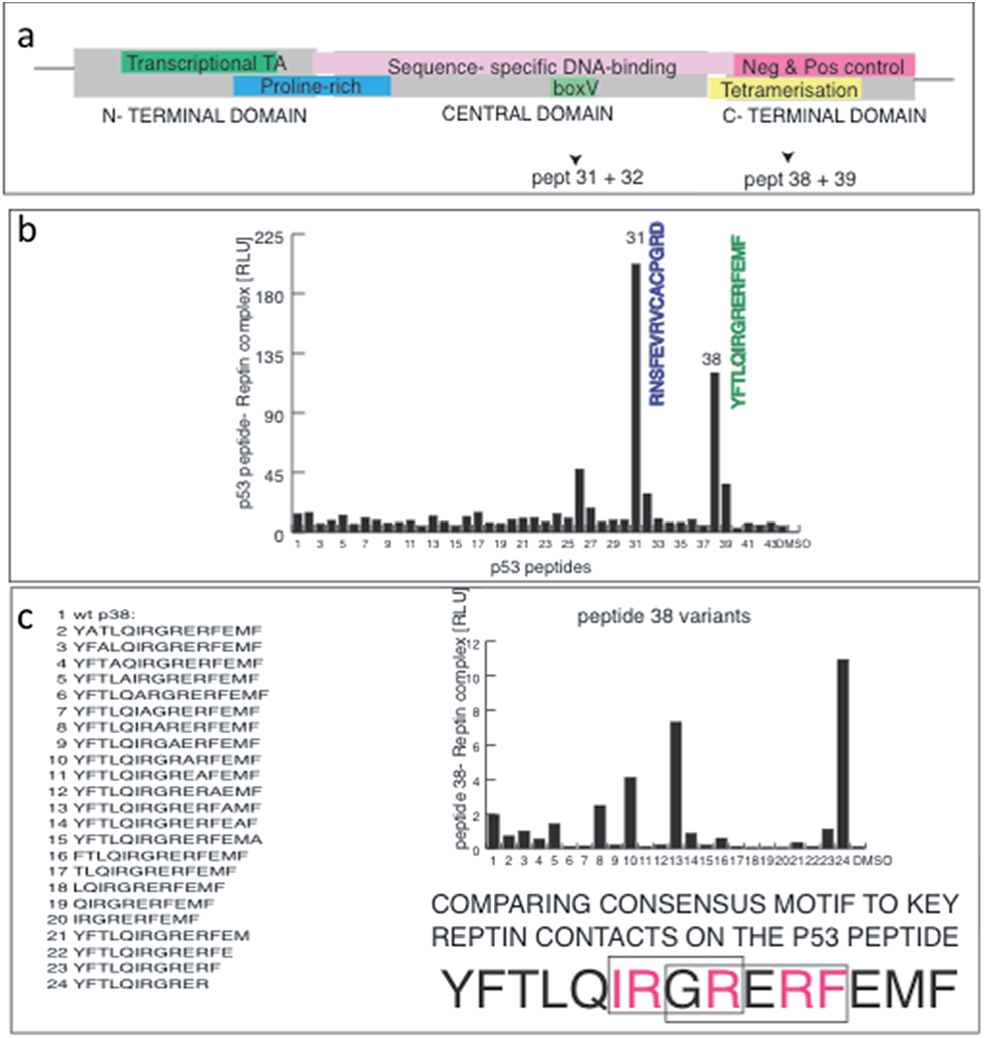
Fine mapping of the dominant linear peptide docking site of reptin on p53. (a) The domain structure of p53 including the sites bound by MDM2 (in green), proline rich motif (blue), the specific DNA-binding domain (pink); tetramerization domain (yellow); and the C-terminal regulatory domain (in red). The arrows highlight the two binding sites mapped for reptin (in panels below). (b) An overlapping series of synthetic biotinylated peptides derived from the open reading frame of p53 were captured on streptavidin coated solid phase and reptin binding was measured as indicated in the methods. Two domain regions from p53 bound to reptin and mapped to the BOX-V domain (RNS…GRD) and to the tetramerization domain (YFT…EMF). The latter peptide contain two repeats of the φRERφ or LRER[L/G] motifs identified from the ligand responsive peptides using MEME (Fig. 4). (c) Alanine scan mutagenesis of peptide 38 identifies important amino acids for reptin binding to p53; in the core sequence IRGRERFEMF, mutating IRGR or the overlapping RERF motif abrogates reptin binding to p53. This functional alanine mutagenesis is consistent with the MEME derived peptide motif from the deep sequencing.

In order to fine map the reptin binding site on p53, overlapping biotinylated peptides from human p53 (Fig. 5a and b) were probed with reptin to evaluate binding. Two dominant peptides (Fig. 5b), one which overlapped with the known MDM2 binding site in the central domain of p53 (peptide 31) and one with a motif in the tetramerization domain of p53 (peptide 38) bound to reptin. This provides at least two docking sites for reptin on p53. Alanine scanning mutagenesis of peptide 38 revealed that the key amino acid contacts required for efficient reptin binding to p53 form the core φRERφ motif (Fig. 5c). These data further confirmed that our peptide combinatorial screen linked with next-generation sequencing can identify dominant docking sites for reptin on substrates and that Liddean can induce changes in reptin binding activity to important proteins such as AGR2, HSP90, ciliary proteins and p53.

### Evaluation of the effects of Liddean on reptin–pontin interactions in a cell-based assay

**Fig. 6:**
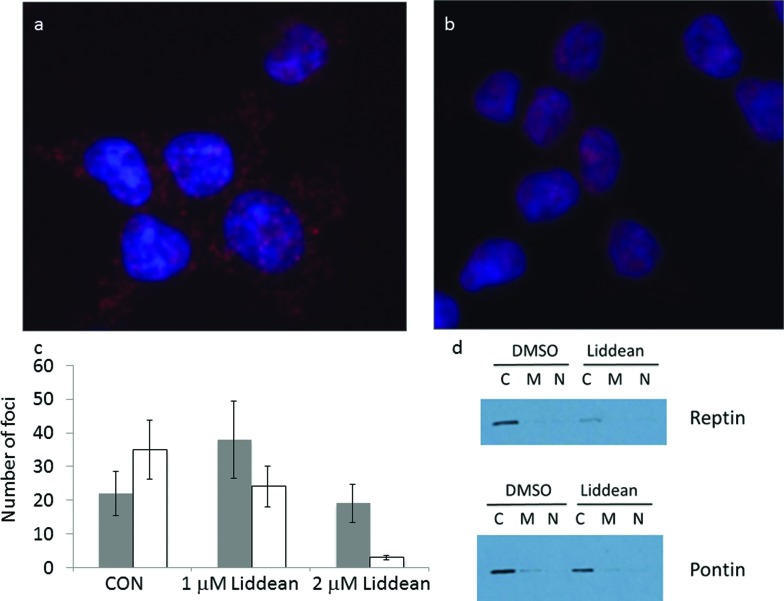
The effects of Liddean on reptin–pontin interactions in cell models using the proximity ligation assay. HCT116 cells were processed using the proximity ligation method to identify whether reptin forms a PPI in cells and the images are superimposed using DAPI to highlight the nuclear (blue) or cytosolic foci location. The data highlight the foci of: (a) reptin–pontin in DMSO treated cells; (b) reptin–pontin foci in cells treated with 2 µM Liddean. (c) Number of cytoplasm (black bar) and nuclear (white bar) foci in the absence of presence of Liddean (1 µM and 2 µM). See Fig. S11† for the image of HCT116 cells in the presence of 1 µM Liddean; (d) immunoblots that show the amount of reptin or pontin after chemical fractionation into cytoplasmic, mitochondrial, and nuclear fractions.

In the final part of this study we assessed whether Liddean could be used to study reptin function in cells. To do this reptin's interaction with its most dominant paralog, pontin was observed using proximity ligation assays^29^ in HCT116 cells. The complex between reptin and pontin was found to be largely cytoplasmic in the absence of Liddean (Fig. 6a; DMSO only). By contrast, Liddean (used at up to 2 µM) led to a substantial reduction in the cytoplasmic reptin–pontin foci and a change from clear punctate nuclear foci to aggregated reptin–pontin foci (Fig. 6b, c and S11†). Similar results were observed in p53-null cells (Fig. S12a–c†). Reptin and pontin expression levels were found to be equivalent with or without Liddean with the majority of both proteins being in the cytoplasmic fractions, relative to the mitochondrial and nuclear fractions using chemical fractionation (Fig. 6d). However, since the nucleus can be leaky in such chemical fractionation experiments, we also evaluated reptin and pontin proteins using immunofluorescence (IF; Fig. S12d–g†). The total amounts of reptin or pontin proteins also remained relatively unchanged in the absence or presence of Liddean (Fig. S12d–g†) and as defined by immunoblotting using urea lysis buffer (Fig. S12h and i†). Although the IF demonstrates a largely cytosolic reptin pool (Fig. S12d and f†), pontin does indeed show mixed cytosolic and nuclear pools (Fig. S12e and g†) suggesting the chemical fractionation does induce a leaching of pontin into the cytosol. These data together indicate that Liddean does not induce global changes in reptin or pontin protein levels or localization, but instead stimulates the production of reptin–pontin complexes in the nucleus. Together with the previous experiments, these data validate Liddean as a novel chemical tool with which to probe both *in vitro* and *in vivo* changes in the functions of the AAA+ protein reptin.

## Discussion

The AAA+ proteins, including reptin, are known for their nucleotide binding sites, oligomeric propensity, and wide range of functions.^5^ Here we built on the observation that nucleotide binding is known to regulate the oligomerization status of reptin. In particular, we were interested in exploring whether by modifying reptin's oligomeric state we would change the proteins it interacts with.^30^ A number of assays could have been used to screen for novel reptin ligands, including helicase activity, ATPase activity, fragment based drug discovery, and/or high throughput competitive binding with nucleotide ligands.^31^ Indeed, a recent approach has identified small molecule inhibitors of the ATPase activity of the reptin paralog, pontin.^32^ Instead we used an *in silico* screen to predict small molecules that would target the deep nucleotide binding pocket (Walker A site) that is a characteristic feature of oligomeric AAA+ proteins. The identified hit (compound **1**) was subjected to SAR analysis guided by an ELISA based PPI assay to generate the chemical tool, Liddean. The SAR data and results from hydrogen–deuterium (H–D) exchange experiments supported our proposal that Liddean binds in the Walker A site of reptin. In addition, Liddean stabilised higher order oligomers of reptin as evidenced by (i) the estimated mass using a denaturing gel electrophoretic screen and (ii) the use of HDX experiments that revealed suppression of deuterium incorporation at the previously reported dimer interface.

Reptin is known to interact with a variety of chromatin and chaperonin signalling proteins and is often considered important in oncogenesis.^33^ More recently, based on genetic screens, reptin has been linked to the assembly of cilia structures on the plasma membrane.^27,28^ With Liddean in hand, we decided to assess whether it could be used to find and provide details about reptin PPIs. As a proof of principle we confirmed that Liddean-bound reptin modified reptin's known interaction with HSP90. Next we decided to use the stabilized, oligomeric state of Liddean-bound reptin *in vitro* to search for “consensus peptide motifs” using a combination of next generation deep-sequencing and a combinatorial peptide-phage library. Whilst a very information rich dataset was obtained, our focus turned to a novel reptin-interacting motif in the p53 tumor suppressor protein. Independent screens verified that indeed reptin can bind to p53-derived peptides mainly through a peptide motif that is located in the p53 tetramerization domain (Fig. 4 and 5). As a further demonstration of the utility of our approach, it was also noted that peptide motifs in proteins of the cytosolic cilia machinery were identified. On-going studies will probe the details of these proposed interactions.

Whilst Liddean had proved a very useful tool *in vitro*, we wanted to assess whether it could also be used in cells. A proximity ligation assays^29^ was therefore used to assess the effect of Liddean on the interaction of reptin with its dominant partner, pontin, in cells. This technique enabled us to observe directly the reptin–pontin complex in the cytoplasm of cancer cells. Interestingly, reptin formed significantly more nuclear foci with pontin on addition of Liddean. Whilst an explanation for this observed redistribution remains challenging, dramatic changes in protein expression levels or localization have been ruled out.

## Conclusions

In discovering and subsequently using Liddean, our chemical biology platform has provided novel insights into the PPIs associated with the important human protein reptin. Ultimately, identifying reptin's complete PPI network and explaining how the network is controlled is central to understanding its role in normal and disease processes. An important concept relating to PPIs is that small linear peptide motifs can form dynamic and specific docking sites for a protein.^34–36^ Small molecule stabilization/destabilization of these motifs provides a promising approach towards overall modulation of protein function.^1^ Indeed, there are PPI drug leads emerging that are being applied in the clinic; the most notable of which targets the linear peptide motif-binding groove of the MDM2 oncoprotein.^37^ As dynamic linear peptide-motif based PPIs form a vast untapped landscape in biology and medicine,^38^ approaches that facilitate the discovery of such interactions will provide new avenues to impact on drug discovery programs.^39^ We believe that the approach we have outlined in this report is applicable to other members of the AAA+ superfamily.

## Materials and methods

### Protein–protein interaction assays of reptin

The expression and purification of reptin protein was carried out as previously described^17^ and as discussed in Fig. S13† with the following exceptions. The cells were initially put into a buffer containing 50 mM HEPES pH 8.0 and 10% sucrose before being snap frozen, the rest of the components of the lysis buffer were then added with the exception of Triton X-100 which was added at a 0.1% concentration rather than 0.5%. The lysate was incubated with glutathione beads for 150 minutes at 4 °C with rotation before the washes were carried out. The protease used to cleave the reptin from the beads was HRV 3C. Biotinylated AGR2 derived peptides (or p53 where indicated) were coated overnight onto streptavidin coated wells and reptin binding was measured in buffers as described previously.^17^ All biotinylated peptides were obtained from Chiron Mimotopes (Australia). When small molecules were evaluated to the indicated final concentrations (balanced with DMSO carrier), reptin was added immediately to the reaction well to allow binding competition to take place in the presence of the AGR2 (or p53) peptide. The wells were washed^17^ and bound reptin was detected using a reptin antibody coupled to anti-rabbit secondary antibody and chemiluminescence. Binding activity was quantified by chemiluminescence using a Fluoroskan Ascent FL Labsystems. For measuring the effects of ligands on the SDS-resistant oligomerization state of reptin using SDS gel electrophoresis, reptin protein (1 µg) was added to buffer B (25 mM HEPES, pH 8.0; 10% glycerol, 10 mM KCl, 1 mM DTT) with the indicated amounts of ADP or Liddean. Following incubation at room temperature for 60 minutes, samples were processed for electrophoresis as indicated in the Fig. S8† legend.

### Hydrogen–deuterium exchange mass spectrometry

Deuteration of the reptin either free or in complex with Liddean was initiated by a sequential dilution into deuterated water with 0.1% DMSO final concentration. The molar ratio between reptin and Liddean was 1 : 5 (as summarised in Fig. S7†). The exchange was done at 21 °C and was quenched by the addition of 1 M HCl in 1 M glycine at 1 min and 5 minutes followed by rapid freezing in liquid nitrogen. Each sample was quickly thawed and injected onto an immobilized pepsin column (15 µl bed volume, flow rate 20 µl min^–1^, 0.1% formic acid in water). Peptides were trapped and desalted on-line on a peptide microtrap (Michrom Bioresources, Auburn, CA) for 1 minute at a flow rate 20 µl min^–1^. The peptides were eluted onto an analytical column (Jupiter C18, 1.0 × 50 mm, 5 µm, 300 Å, Phenomenex, CA) and separated by a linear gradient. The injection, switching valves, immobilized pepsin column, trap cartridge, and the analytical column was kept at 1 °C in a cool box (within the robotics system). Mass spectrometric analysis was done on an Orbitrap Elite mass spectrometer (Thermo Fisher Scientific) with ESI ionization on line connected with a robotic system based on a HTS-XT platform (CTC Analytics company). The instrument was operated in a data-dependent mode for peptide mapping (HPLC-MS/MS). Each MS scan was followed by MS/MS scans of the top three most intensive ions from both CID and HCD fragmentation spectra. Tandem mass spectra were searched using SequestHT against the cRap protein database (; ftp://ftp.thegpm.org/fasta/cRAP) containing sequence of reptin protein. Sequence coverage was visualized with Proteome Discoverer 1.4 software (Thermo Fisher Scientific). Analysis of deuterated samples was done in HPLC-MS mode with ion detection in the orbital ion trap and data were processed in HDX Workbench. Graphs showing deuteration kinetics were plotted by DrawHDXPlot (MSTools).

### Combinatorial peptide phage screen

Peptide phage was carried out using the 12-*mer* Ph.D.™ Phage display library (New England Biolabs). The surface panning procedure (direct target coating) was carried out as instructed by the manufacturer's protocol with an additional protein capture step. The micro titer wells were coated as directed with a rabbit anti-reptin polyclonal antibody overnight. The wells were then washed three times with tris buffered saline with 0.1% Tween-20 (TBST). The wells were then blocked with 3% bovine serum albumin in TBS for 1 hour. Washes were once again carried out before the addition of reptin with and without ligand (either ADP or Liddean at 100 µM) and incubated for one hour at RT. Washes were then carried out 6 times with TBST, containing ligand in the ligand treated wells. The phage pool was then added, again containing ligand if required, and incubated for 1 hour. Non-binding phage was removed and the wells were washed 10 times with PBST prior to the elution of the phage as directed by the manufacturer. Amplification and titering of the phage was also carried out with each round of panning as per the manufacturer's instructions. Titering was carried out to ensure that the phage pool being panned was not greater than that of the original pool once it had been amplified and to check for white-type lytic phage contamination. Polymerase chain reaction (PCR) and deep sequencing of phage was carried out in the following stages; (i) PCR was used to amplify phage DNA from each round of screening using the primer bar codes in table code (Table S3†) that have an Illumina adaptor sequence and a 3 letter bar code; (ii) equal amounts of DNA was gel purified on a 2% agarose gel to create a pool (5 µg) that was sequenced by Otogenetics (USA). Fastq files were then captured using a custom tool programmed in Java language (script available upon request) that was used to extract amino acid sequences from raw NGS reads. Only forward reads were processed (as reverse reads do not capture the bar code). Barcode and mimotope DNA sequences were extracted from reads that passed quality control based on exact match search for bordering sequences. All sequences having nonsense (not in list) bar code were filtered out. Mimotope sequences having inappropriate length or containing nonsense codons (stop codons as well as some other “forbidden” codons that should not be present according to New England BioLabs phage library manual) were filtered out. Sequences passing these filters were translated, grouped by resulting peptide sequence and sorted as indicated in the tables.

### Duo-link proximity ligation assay

Cells were grown on coverslips until they reached around 30% confluency upon which the small molecule Liddean was transfected into cell and incubated for 24 hours (transfection was performed using 1 or 2 µM molecule with DMSO control balanced in 100 µl of DMEM containing attractene carrier). Cells were fixed with 4% paraformaldehyde dissolved in PBS and permeabilized using 0.25% Triton X-100 in PBS. Duolink II (green) assay from Olink Bioscience was carried out following manufactures instructions. Briefly, unspecific antibody binding was blocked by the addition of 3% BSA (w/v) in PBS for 30 minutes at room temperature. Primary antibodies were diluted in 3% BSA (w/v) in PBS and added to the cells overnight at 4 °C. Negative controls include no primary or no secondary antibodies which gave rise to no foci in subsequent assay processing (data not shown). PLA probes were added to cells and incubated to 1 hour at 37 °C. Ligation mix was added for 30 minute at 37 °C followed by the polymerase amplification mix for 2 hours at 37 °C. Coverslips were mounted in S3023 Mounting medium (Dako). Results were visualized using a BX51 (Olympus) fluorescent microscope. Antibodies and PLA probes include; anti-reptin/TIP49B/RUVB2 rabbit antibody ab36569 (abcam) (1 : 250 dilution); monoclonal anti-pontin 5G3-11 (Sigma) (1 : 250 dilution); Duolink II PLA probe anti rabbit PLUS (Olink) (1 : 10 dilution); and Duolink II PLA probe anti mouse MINUS (Olink) (1 : 10 dilution).
