# Supplementary material for: Discovery of a novel ligand that modulates the protein–protein interactions of the AAA+ superfamily oncoprotein reptin
Source: Chem Sci. 2015 Mar 20;6(5):3109–16. doi: 10.1039/c4sc03885a (PMC5490336; doi:10.1039/c4sc03885a)
Supplement: Supplementary file 1 [file SC-006-C4SC03885A-s001.pdf]

Electronic Supplementary Material (ESI) for Chemical Science.
This journal is © The Royal Society of Chemistry 2015

Supporting Information

Discovery of a novel ligand that modulates protein-protein interactions of the AAA+ superfamily protein Reptin

Alan R Healy, Douglas R Houston*, Lucy Remnant, Anne-Sophie Huart, Veronika Brychtová, Magda M Maslon, Olivia Meers, Petr Muller, Adam Krecji, Elizabeth A Blackburn, Borek Vojtesek, Lenka Hernychova, Malcolm D Walkinshaw, Nicholas J Westwood* & Ted R Hupp*

1 Contents

2 Additional Experimental Information 4

2.1 *In Silico* Screening and Hit Identification 4

2.2 Chemical Synthesis and SAR Study 8

2.3 Analysis of Liddean-induced changes in the conformation and oligomeric state of reptin 13

2.4 H/D Exchange Raw Data 14

2.5 Discovering new Liddean-Stimulated Peptide Docking Motifs on Reptin 24

2.6 Proximity Ligation Assay 27

2.7 Purification and thermal shift analysis of the binding of ADP to wt-reptin. 32

3 Virtual Screening Materials and Methods 33

3.1 Virtual Library Collation 33

3.2 Virtual screening workflow 33

3.3 Redocking Control Experiments 34

4 Synthesis and Characterization of Compounds 36

4.1 General Considerations 36

4.2 General Procedures 37

4.3 Experimental procedures 37

5 ^{1}H NMR Spectra of Purchased Compounds (Chembridge) 54

6 References 58

List of Supplementary Figures, Tables and Schemes.

Page S4	**Figure S1.** Identification of small molecules targeting reptin using an *in silico* screening programme.
Page S5	**Table S1.** The 30 hits selected for testing in the reptin-AGR2 peptide binding assay.
Page S6	**Figure S2.** The effect of small molecules on the reptin-AGR2 peptide binding activity.
Page S7	**Figure S3.** Computational docking of Liddean into the ATP pocket of Pontin/Reptin.
Page S8	**Scheme S1.** Synthetic modification of Compound **1** to optimize its bioactivity towards reptin.
Pages S9-10	**Figures S4(a-d).** Structures of analogs of initial hit **1** prepared during the SAR study.
Page S11	**Scheme S2.** Synthesis of C ring analogs (**S36** and **S37**).
Pages S12-13	**Figure S5.** Screening of the small molecule series in a reptin-AGR2 peptide interaction ELISA and a discussion of the SAR data that was generated.
Pages S13-14	**Figure S6.** A more detailed view of changes in the rate of hydrogen/deuterium exchange around the nucleotide pocket.
Pages S14-15	**Figure S7(a and b).** Sequence coverage after HDX Workbench evaluation and an overview of deuteration of reptin peptides after 30 minutes in the absence of Liddean.
Pages S16-22	**Table S2.** Output data from hydrogen-deuterium Exchange at the 1 and 5 minute time point.
Page S23	**Figure S8.** The effects of Liddean on the oligomerization dynamics of reptin.
Page S24	**Figure S9.** Discovery of new Liddean-dependent interaction motifs for reptin.
Page S25	**Table S3.** PCR Primer bar codes.
Page S26	**Figure S10.** Additional ciliopathy proteins present in the list of human proteins which contain consensus sites identified by our Liddean-bound reptin screen.
Page S27	**Figure S11.** The effects of Liddean on reptin-pontin protein interactions in HCT116 p53+/+ cells at 2μM.
Page S28-31	**Figure S12.** The effects of Liddean on reptin-pontin protein interactions in HCT116 p53-/- cell models using the proximity ligation assay, immunofluorescence and western blotting.
Pages S32-33	**Figure S13.** Purification and thermal shift analysis of the binding of ADP to wt-reptin.

2 Additional Experimental Information

2.1 *In Silico* Screening and Hit Identification

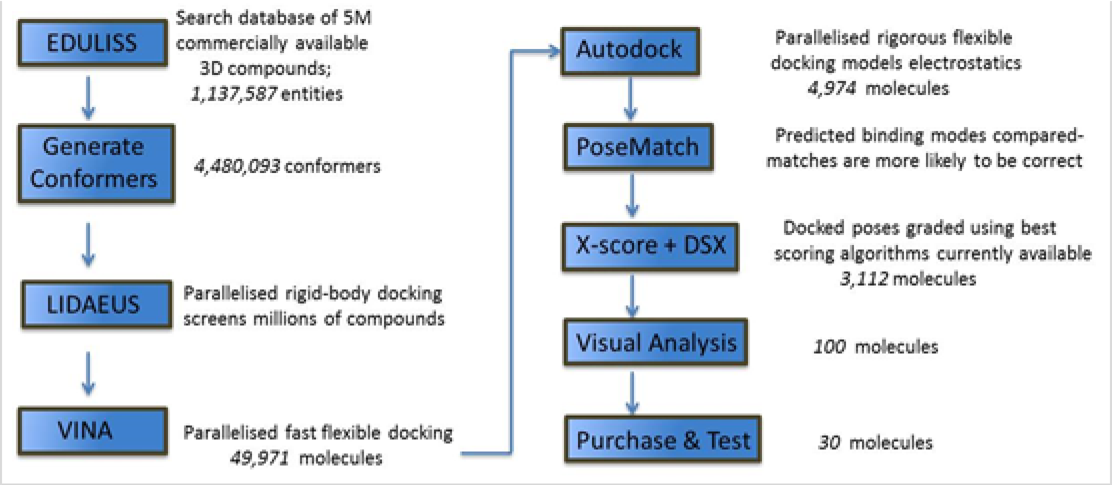

Fig. S1. Identification of small molecules targeting reptin using an *in silico* screening programme. (a) Workflow diagram of the *in silico* screening process. Numbers of molecular entities (compounds or conformers) are indicated at each stage. At the start of this research, no X-ray crystal structure of the reptin protein was available, therefore the X-ray crystal structure of pontin (PDB 2C90), a highly conserved paralog of reptin was used. The rigid-body docking program LIDAEUS[1] docked a conformer virtual library of 4.5 million compounds into pontin's Walker A site. The results were ranked based on the LIDAEUS score and the top 49971 compounds were redocked using Vina and Autodock. A "rank-by-rank" consensus protocol prioritized hits, culminating in the visual inspection of the binding mode of the top 100 hits. The hits were divided into 14 classes based on structural similarity and examples of each class were bought for testing (**Table S1**). This screen was revisited when an X-ray crystal structure of reptin in an alternating heterododecameric complex with pontin was reported (PDB 2XSZ). Comparison with the structure of hexameric pontin (PDB 2C90) verified the high structural similarity between the reptin and pontin ATP-binding pockets (**Fig. S3a**). However, an important difference was observed in the position of the sensor II residue (Arg400) in reptin compared to pontin (Arg404, **Fig. S3a**). In the pontin structure, Arg404 was folded over the ATP pocket whereas in reptin Arg400 was located such that the Walker A site was more open (**Fig. S3a**). On rescreening the library using the Walker A site open structure of reptin, **1** was not identified as a hit. It has been shown using molecular dynamics that Arg400 in reptin and Arg404 in pontin are flexible and control access to the ATP-site[2]. The *in silico* screen was repeated but this time the side chain of Arg400 was locked over the pocket. This screen predicted that **1** bound to reptin's Walker A site, implicating the correct positioning of Arg400 as essential for its binding. When **1** was docked into reptin keeping Arg400 flexible and using energy minimization methods, the side chain of Arg400 folded over the pyridine-oxazolo rings (**Fig. S3b**). The formation of

hydrogen bonds between Arg400 and both the heterocyclic rings and the amide oxygen in **1** was predicted, suggesting that these may be essential for the activity of **1**.

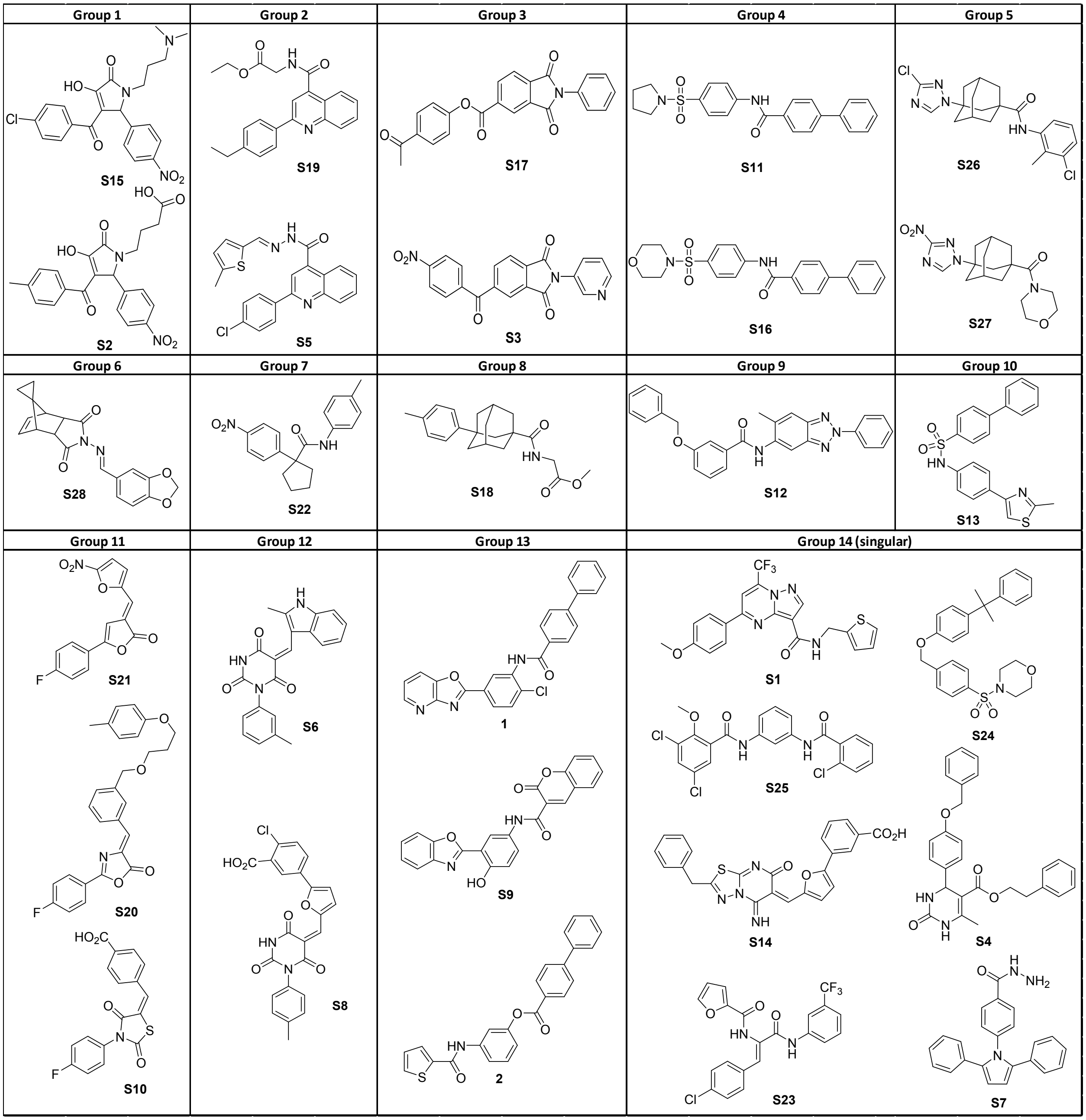

Table S1. The 30 hits selected for testing in the reptin-AGR2 peptide binding assay. Visual analysis of the top 100 hits identified the presence of 14 different groups. Groups 1-13 were classified based on a similar structural core. 1-3 analogues were purchased from each group for testing depending on the size and structural diversity of the group. Group 14 contained the remaining analogues that did not contain a common structural core. 7 analogues from this group were purchased for testing.

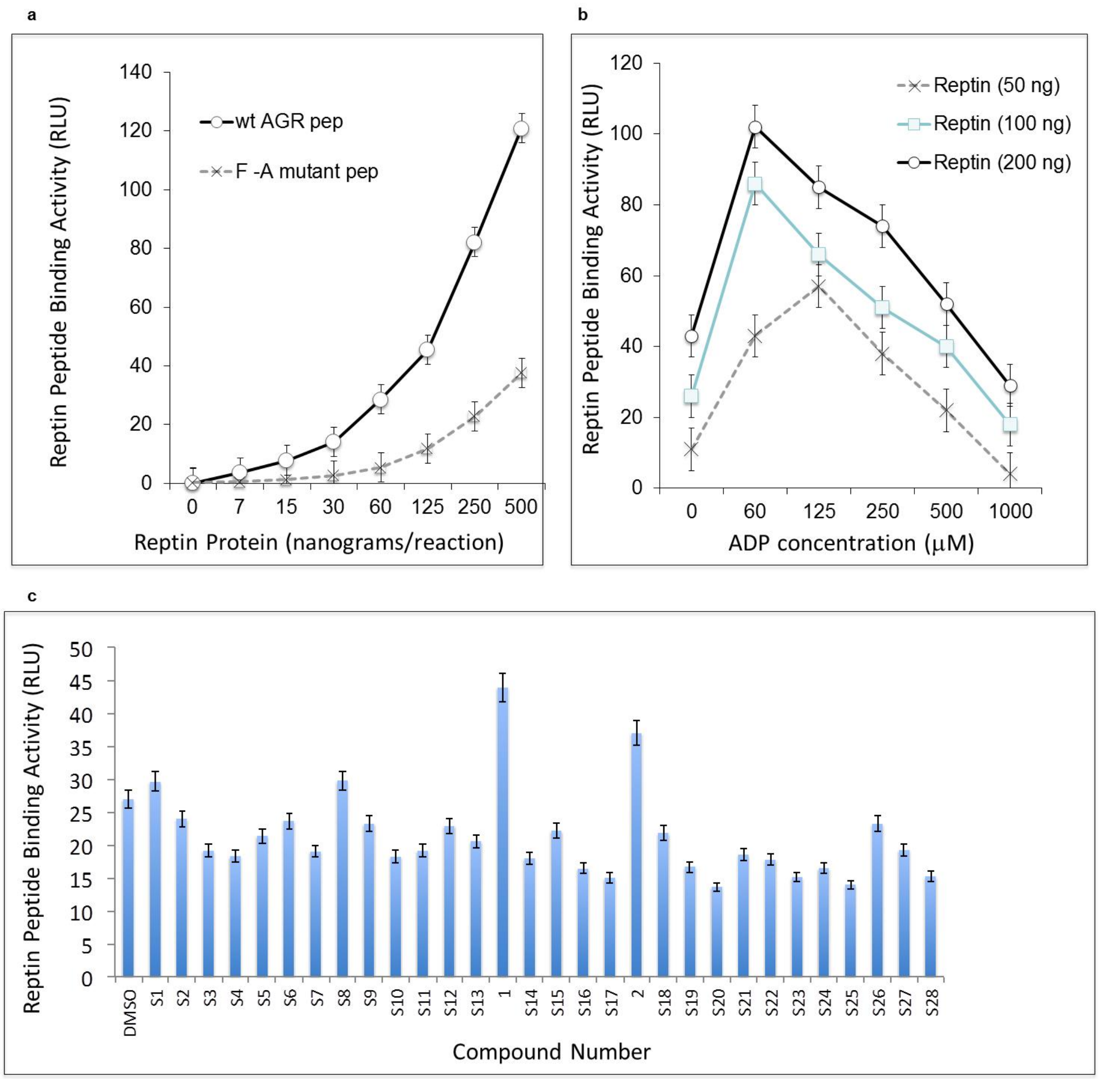

Fig. S2. The effect of small molecules on the reptin-AGR2 peptide binding activity. (**a** and **b**) Development of a protein-protein interaction assay that measures the effects of ligands on the biochemical function of reptin. (**a**) A biotinylated version of the peptide binding motif from wild-type or F104A mutant AGR2 (104-FVLLNLVY-111) was bound onto streptavidin coated wells and reptin was titrated followed by a primary antibody to reptin and a peroxidase linked secondary antibody to measure binding stability of reptin to the AGR2 peptide. The data are plotted as reptin binding activity (in RLU) as a function of reptin protein levels. (**b**) Differing concentrations of reptin (50, 100, or 200 ng) were incubated with increasing concentrations of ADP. Low concentrations of ADP stimulating the AGR2-peptide binding function of reptin, in contrast with higher concentrations which attenuates its binding activity. (**c**) Reptin-AGR2 peptide binding ELISA. The top 30 hits from the *in silico* screen were evaluated in the peptide binding assay at 100 μM. Compounds **1** and **2** (**Table S1**) significantly stimulated reptin's binding activity to the AGR2 peptide relative to the DMSO control.

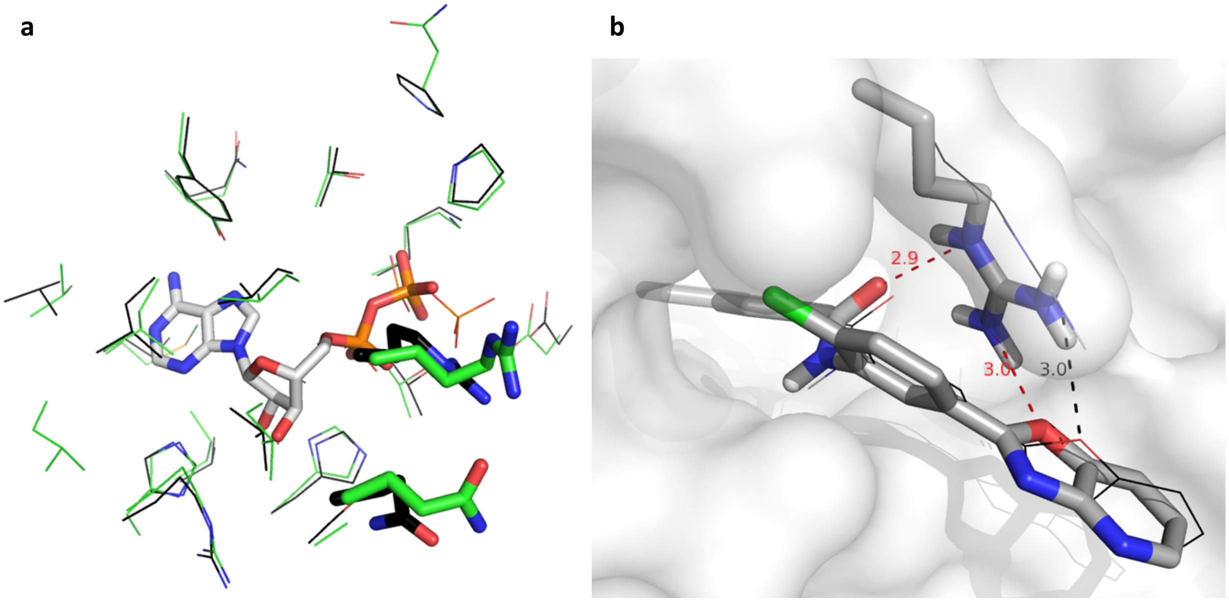

Fig. S3. Computational docking of Liddean into the ATP pocket of Pontin/Reptin. (a) Figure showing an overlay of the residues of the reptin (taken from PDB 2XSZ) and pontin (taken from PDB 2C9O) ATP pockets, highlighting the difference in position of two key side chains. Reptin is colored green, pontin black. The ADP molecule from the pontin structure (PDB 2C9O) is shown as sticks, the ATP from the superposed reptin structure (PDB 2XSZ) as lines. (**b**) Original and optimized Autodock predictions of the **1** binding conformation. The predicted mode from the virtual high-throughput screen is shown as black lines and the hydrogen bond it makes with Arg404 are shown as a black dashed line with its length in Å. The result of the docking with maximal parameters and flexible Arg404 activated is shown as grey sticks. Two hydrogen bonds in orange can be seen between the optimized **1** docking pose and the side chain. The calculated angle associated with the NH-(O=C) hydrogen bond is 132.1°.

2.2 Chemical Synthesis and SAR Study

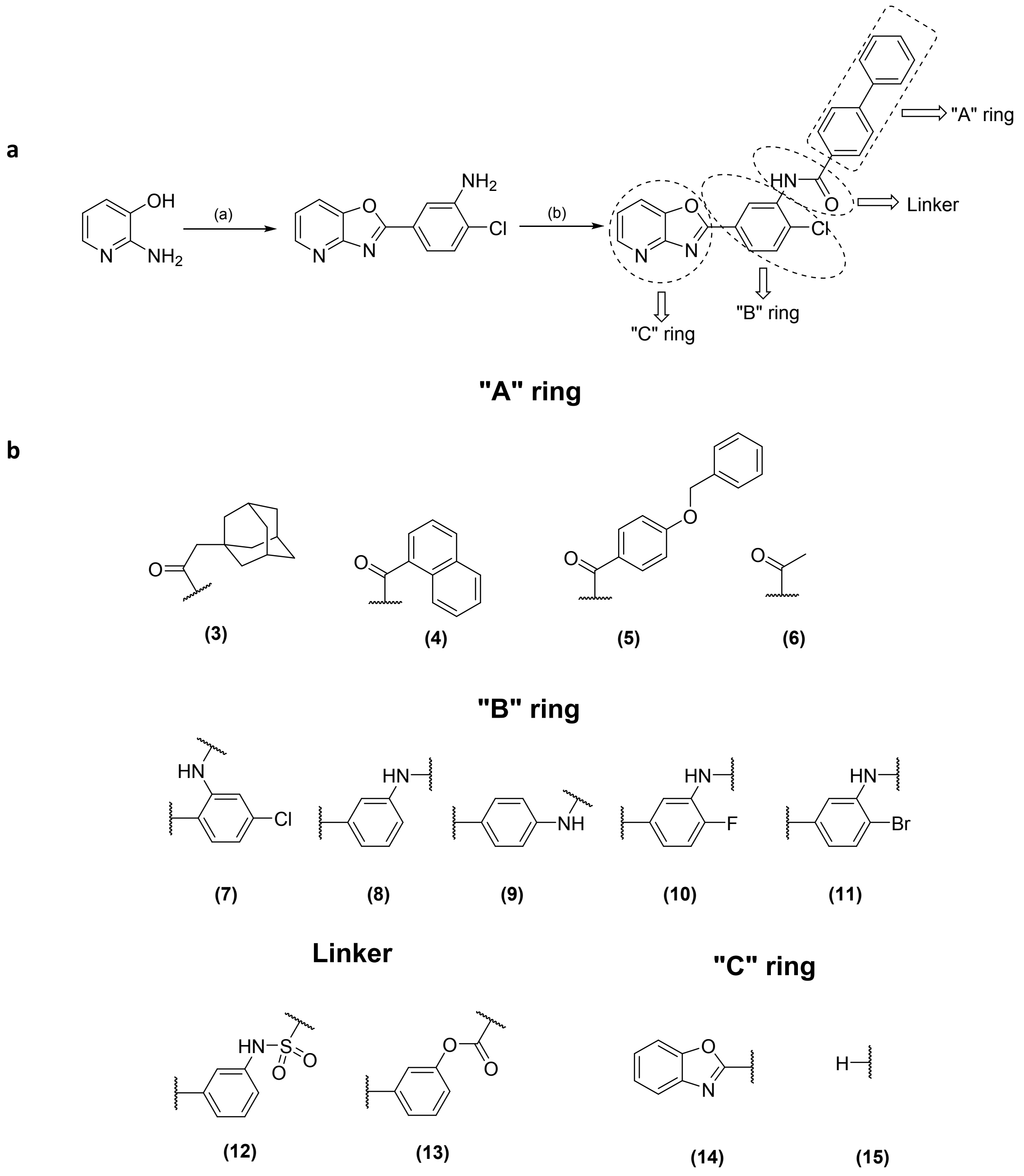

Scheme S1. Synthetic modification of Compound 1 to optimize its bioactivity towards reptin. (**a**) Synthesis of **1**. (i) 3- amino-4-chlorobenzoic acid, PPA, 200 °C, 4 h. (ii) H_2O, $NaHCO_3$, 55%. (b) biphenyl-4-carbonyl chloride, DIPEA, DCM, r.t., 24 h, 57%. (**b**) A selection of the analogues tested in the SAR study. Compounds (**3** & **4**) were purchased from Chembridge. Compounds (**5** & **15**) was synthesized in an analogous manner to **1**. See **Fig. S5** for the ELISA results of analogs **7**,**8**,**10**,**11**,**12** and **13** and SAR analysis.

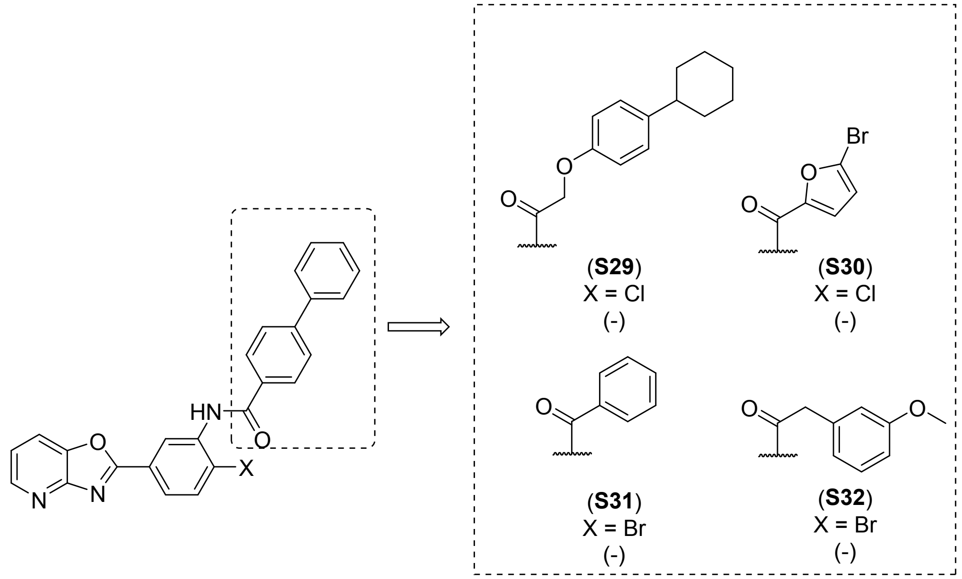

Fig. S4a. Additional “A” ring analogs tested in the SAR study. Compounds (**S29** & **S30**) were purchased from Chembridge. Compound (**S31** & **S32**) was synthesized in an analogous manner to **1** (**Scheme S1**). These analogs were inactive in the reptin-AGR2 peptide binding ELISA.

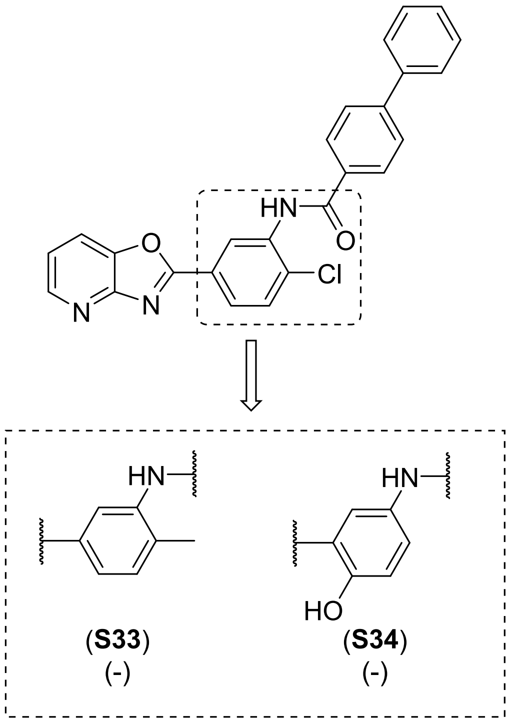

Fig. S4b. Additional B ring analogs tested in the SAR study. Compounds **S33** and **S34** were synthesized in an analogous manner to **1** (**Scheme S1**). These analogs were inactive in the reptin-AGR2 peptide binding ELISA.

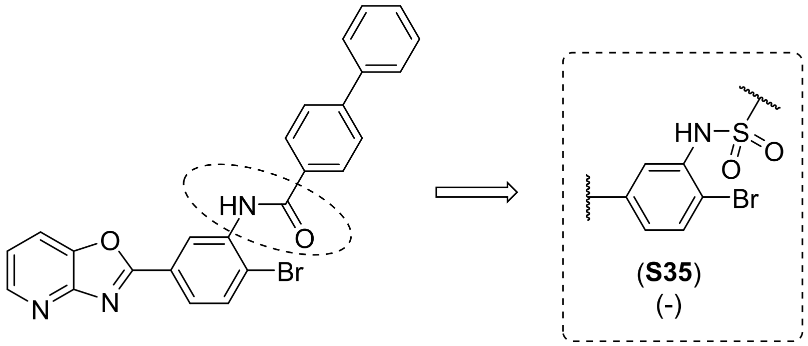

Fig. S4c. Additional linker unit analogs tested in the SAR study. Compound **S35** was synthesized in an analogous manner to **1** (**Scheme S1**). **S35** was inactive in the reptin-AGR2 peptide binding ELISA.

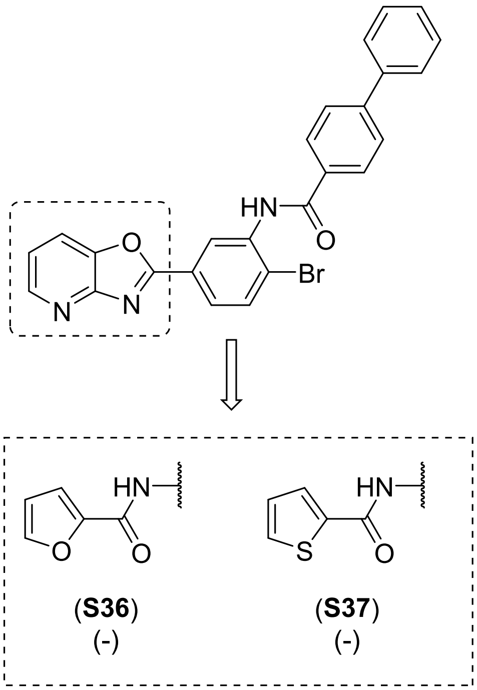

Fig. S4d. Additional B ring analogs tested in the SAR study. Compounds **S36** and **S37** were synthesized as shown in **Scheme S2**. These analogs were inactive in the reptin-AGR2 peptide binding ELISA.

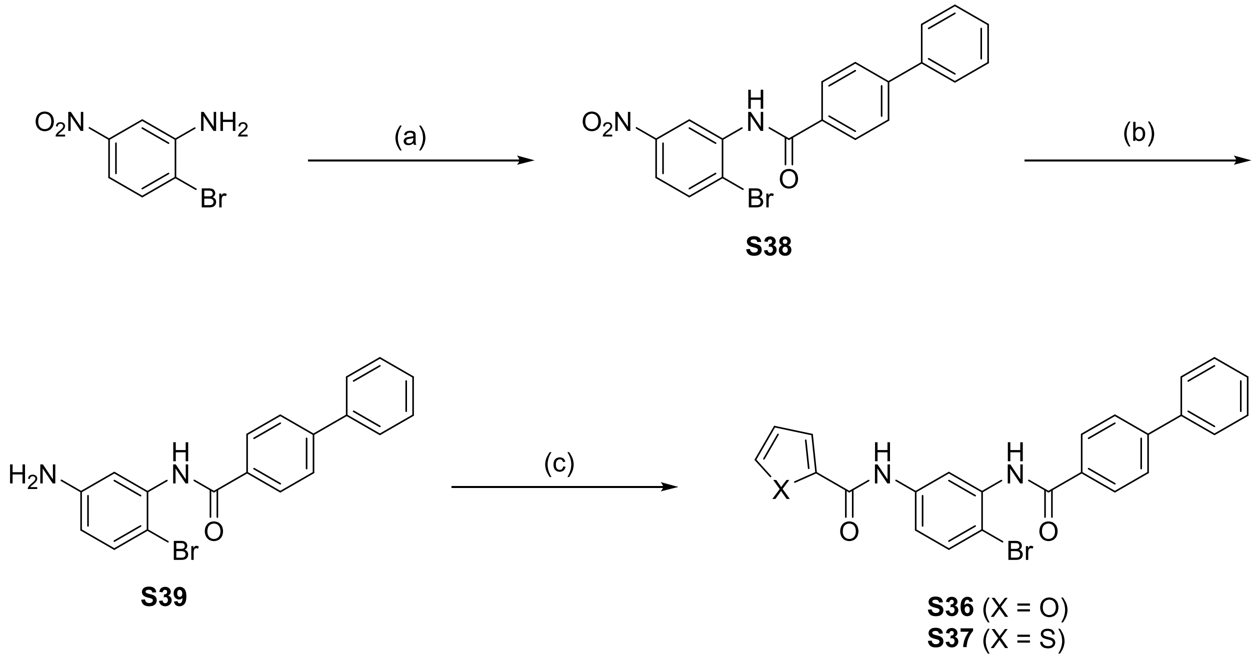

Scheme S2. Synthesis of C ring analogs (S36 and S37). (a) Biphenyl-4-carbonyl chloride, pyridine, DCM, r.t., 18 h, 53%. (b) Fe, NH_4Cl, MeOH, H_2O, 100 °C, 3 h, 90%. (c) 2-Furoyl chloride (**S36**) or 2-thiophene chloride (**S37**), pyridine, DCM, r.t., 18 h, **S36** = 72%, **S37** = 85%.

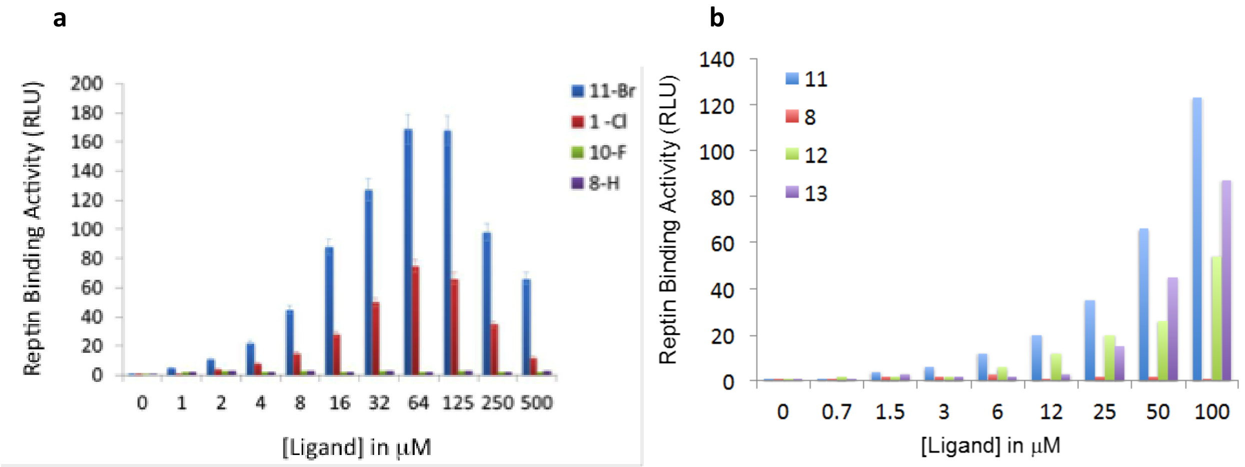

Fig. S5. Screening of the small molecule series in a reptin-AGR2 peptide interaction ELISA. A biotinylated version of the peptide binding motif in AGR2 (F104A) was bound onto streptavidin coated wells and reptin (see Figure S13 for production of the recombinant enzyme) was titrated followed by addition of a primary antibody to reptin and a peroxidase linked secondary antibody to measure binding of reptin to the AGR2 peptide. The data are plotted as reptin binding activity (in RLU) as a function of ligand concentration. (**a**) The effects of a halogen substitution in *ring B* on the binding activity of reptin (see Scheme S1a for a description of the nomenclature used in this figure legend and Scheme 1b for the structures of compounds **8**, **10** and **11**; the structure of compound **1** is shown in Figure 1 of the main manuscript; for the synthesis of compounds **1**, **8**, **10** and **11** see chemistry experimental section below). (**b**) The effects of *linker* modification on the binding activity of reptin. (see Scheme S1b for the structures of compounds **8**, **11**, **12** and **13**; for the synthesis of compounds **8**, **11**, **12** and **13** see chemistry experimental section below)

Brief discussion of the SAR study.

(A) Testing of compounds **3**-**6** (Scheme 1b) and **S29**-**S30** (Figure S4a) in this assay showed that they were inactive (**data not shown**) indicating that activity was very sensitive to changes in the A region. This observation led us to retain the A-ring from the initial hit **1**.

(B) B-ring analogs **7-11** (Scheme 1b) and **S33/S34** (Figure S4b) explored two issues: i) the relative positions of the A and C rings and ii) the role of the halogen. Changing the position of the amide group relative to the chlorine from the *meta*- to the *ortho*-position (c.f. **1** vs **7**) was not tolerated (**data not shown**) as expected based on the predicted binding mode. Replacement of the chlorine in **1** with a hydrogen resulted in loss of activity (**1** vs **8; Fig. S5a**) and this could not be rescued by repositioning of the amide group *para*- to the pyridine-oxazolo ring (in **9**; **data not shown**). Variation of the halogen showed that an increase in atomic radius led to increased activity (**10**(F) < **1**(Cl) < **11**(Br)) with bromine analog **11** being the most active (**Fig. S5a**). A dose titration of the halogen series confirmed a concentration effect on the stimulation and attenuation of the specific reptin-AGR2 peptide binding activity, with as little as 1 μM of **11** showing stimulation (**Fig. S5a**).

(C) Assessment of analogs **12**, **13** (Scheme S1b) and **S35** (Scheme S4c) that contained modified linkers revealed that the activity increased with linker flexibility**.** The ester analog **13** was more active than sulfonamide **12**, whilst amide **8** was inactive (**Fig. S5b**).

(**D)** Studies on the "C" ring found that changes were not tolerated (analogs **14**, **15** (Scheme S1a) and **S36**, **S37** (Figure S4d); **data not shown**), consistent with a requirement for hydrogen bonding of this heterocycle to Arg400 (Fig S3b). This SAR study identified **11**, now called Liddean, as the most active analogue and provided support for the proposed binding mode.

2.3 Analysis of Liddean-induced changes in the conformation and oligomeric state of reptin

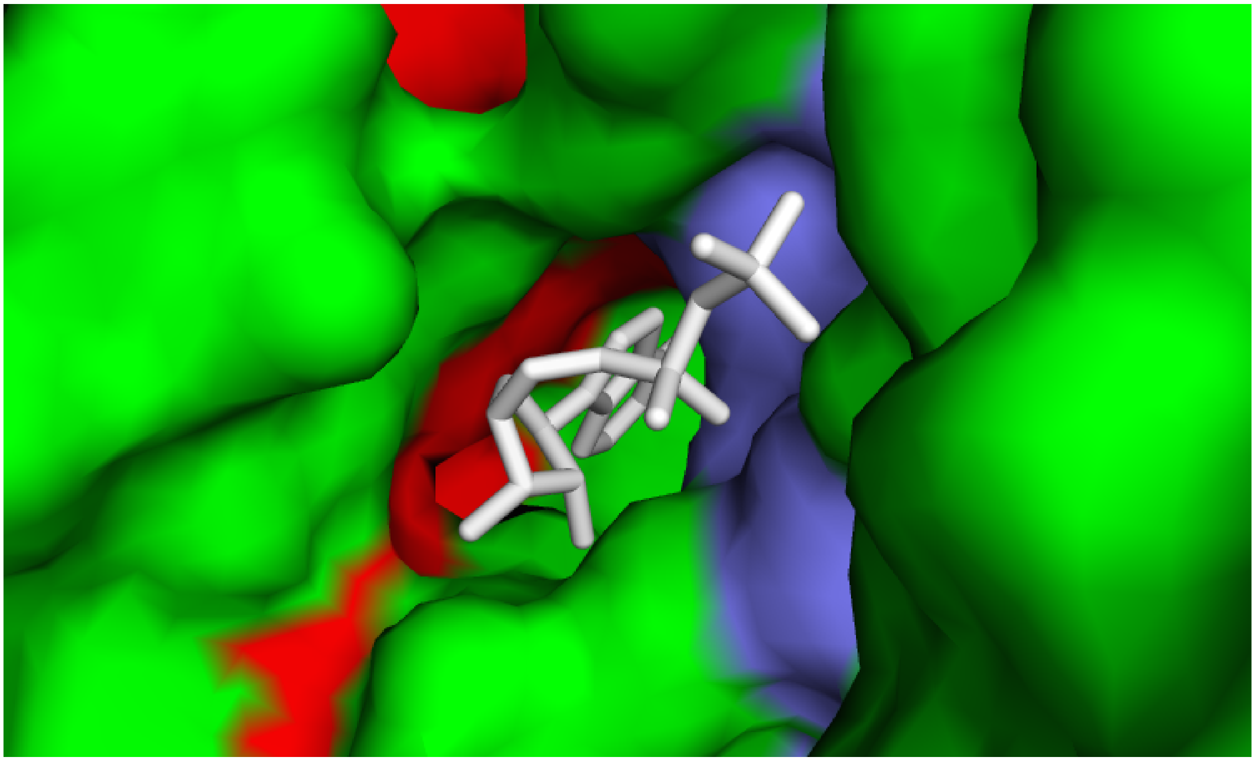

Fig. S6. A more detailed view of changes in the rate of hydrogen/deuterium exchange around the nucleotide pocket. Reptin was incubated with excess Liddean (or DMSO control), diluted with D_2O and incubated over a time course. Acidification followed by pepsin treatment enabled changes in the rate of peptide deuteration to be measured as a function of ligand binding (**Table S4**). The observed suppression of deuterium exchange by Liddean was most pronounced for amino acids 362-375 adjacent to the Walker A site (PDB 3UK6) and for amino acids 428-439 (at the dimer interface) (**Fig. 1d** and **Fig. S6**). Liddean increased the rate of deuterium exchange over many peptic fragments with the most

dominant increase being along the length of the α-helix (81-95) that contacts to the Walker A site (**Fig. 1d** and here). These data were consistent with Liddean binding within the Walker A site. The ATP pocket of reptin (PDB 3UK6) is shown as a green surface. Regions with suppressed deuterium exchange are shown in red and regions with increased deuterium exchange are shown in blue. The ADP molecule bound to reptin is shown as white sticks. PDB 3UK6 was used in the HDX as this part of the project was carried out much later when this structure was available whereas PDB 2SXZ was used for the in silico screening.

2.4 H/D Exchange Raw Data

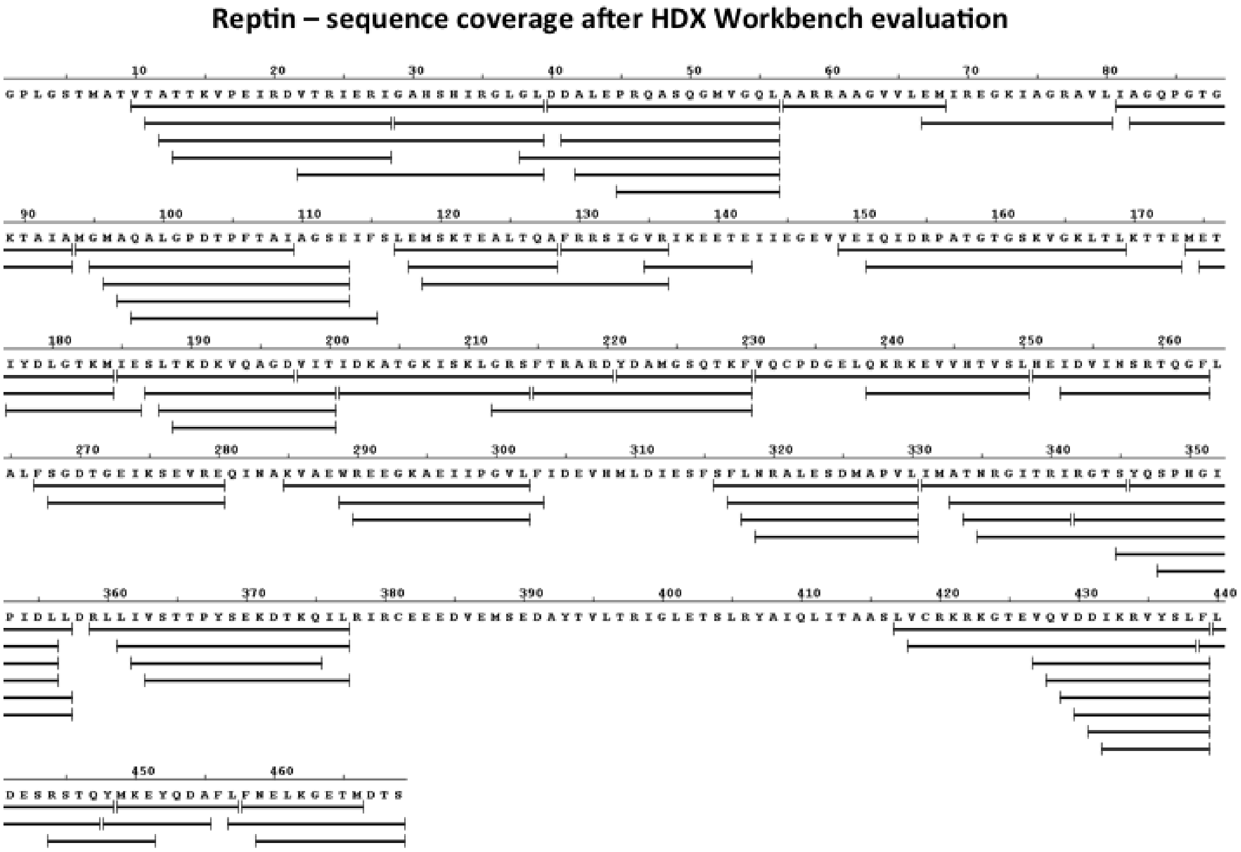

Fig. S7a. Sequence coverage after HDX Workbench evaluation

Apo-Reptin peptides deuteration –30 min in D_2O

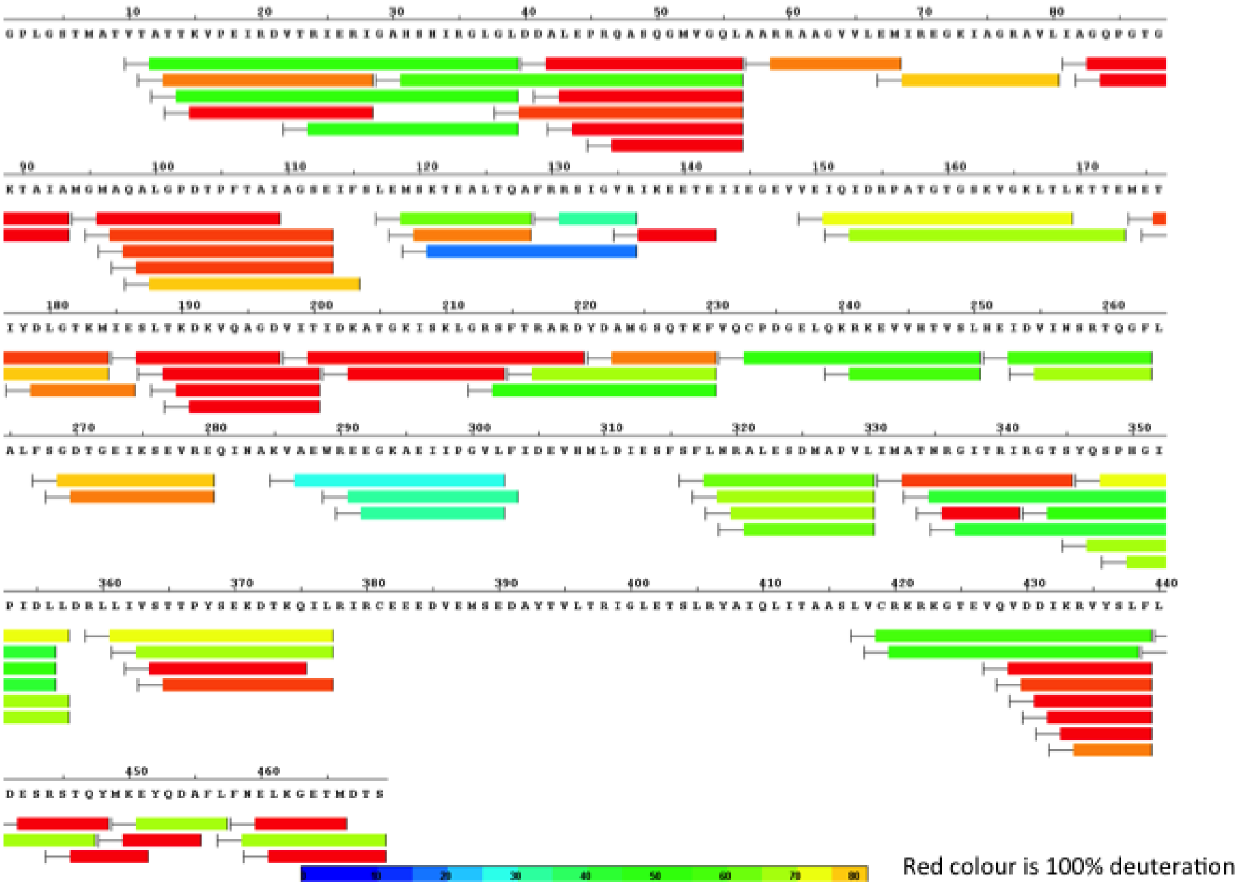

Fig. S7b. Deuteration of reptin peptides after 30 minutes in the absence of Liddean.

Table S2. Output data from hydrogen-deuterium Exchange at the 1 and 5 minute time point. The data is tabulated in columns as: (1) "from to" pinpointing the amino acids with a peptic fragments; (2) time in seconds, with zero the background control reptin along side the % deuteration in the absence or presence of Liddean. The red and the green highlights the most pronounced increases or decreases in deuteration after ligand binding, respectively.

From	To	Time (sec)	Reptin	Reptin + Liddean
10	21	0s	-0.029761905	
10	21	60s	105.3472222	102.6845238
10	21	300s	105.2003968	105.4146825
11	28	0s	0.030357143	
11	28	60s	79.93392857	80.60535714
11	28	300s	80.04821429	80.26964286
12	39	0s	0.054642857	
12	39	60s	48.81107143	46.99821429
12	39	300s	49.2225	45.61178571
22	41	0s	-0.011904762	
22	41	60s	32.6984127	29.83730159
22	41	300s	32.78968254	31.60714286
29	39	0s	0.063492063	
29	39	60s	47.67857143	48.42063492
29	39	300s	46.59920635	49.11904762
32	39	0s	-0.202380952	
32	39	60s	67.72619048	68.50595238
32	39	300s	67.39285714	69.2202381
38	56	0s	-0.034598214	
38	56	60s	65.7734375	71.97879464
38	56	300s	66.18638393	69.67075893
40	56	0s	0.052295918	
40	56	60s	88.24617347	86.69005102
40	56	300s	91.86607143	89.58545918
41	57	0s	-0.012755102	
41	57	60s	108.5178571	108.2142857
41	57	300s	108.4515306	107.5841837
42	56	0s	0.001488095	
42	56	60s	95.91815476	96.99553571
42	56	300s	101.3110119	99.06994048
57	68	0s	-0.101785714	
57	68	60s	12.27321429	21.225
57	68	300s	13.53214286	25.03928571
67	80	0s	-0.035714286	
67	80	60s	46.22321429	49.59821429

67	80	300s	53.81547619	54.85714286
69	80	0s	-0.008035714	
69	80	60s	52.30982143	56.45089286
69	80	300s	55.00446429	60.12053571
81	94	0s	-0.108766234	
81	94	60s	44.41071429	65.43344156
81	94	300s	51.24188312	68.36850649
81	95	0s	0.092261905	
81	95	60s	41.32738095	69.76785714
81	95	300s	46.87202381	72.50892857
85	101	0s	-0.085714286	
85	101	60s	10.08095238	33.65714286
85	101	300s	12.02619048	30.14047619
94	113	0s	0.001116071	
94	113	60s	5.280133929	12.70424107
94	113	300s	5.202008929	10.21986607
95	113	0s	-0.235714286	
95	113	60s	7.723809524	18.57619048
95	113	300s	6.711904762	14.98571429
96	109	0s	-4.728571429	
96	109	60s	90.72142857	89.99285714
96	109	300s	92.92857143	89.25357143
96	113	0s	-0.038265306	
96	113	60s	5.892857143	12.0127551
96	113	300s	5.505102041	9.887755102
97	113	0s	-0.048076923	
97	113	60s	7.229395604	12.61126374
97	113	300s	6.070054945	12.01510989
98	108	0s	0.020408163	
98	108	60s	37.30102041	36.96428571
98	108	300s	51.8877551	57.74489796
98	115	0s	-0.028061224	
98	115	60s	7.081632653	9.658163265
98	115	300s	8.033163265	9.839285714
117	129	0s	-0.173701299	
117	129	60s	16.80681818	21.86850649
117	129	300s	19.03409091	21.82305195
120	128	0s	-0.089285714	
120	128	60s	42.56377551	57.69132653
120	128	300s	41.81377551	65.94642857
129	136	0s	0.026785714	
129	136	60s	29.70535714	32.89583333
129	136	300s	29.77678571	36.7172619
149	174	0s	-0.097826087	

149	174	60s	11.43944099	22.48602484
149	174	300s	10.78416149	19.60093168
151	167	0s	-0.1875	
151	167	60s	53.59821429	50.67091837
151	167	300s	55.56122449	54.32142857
151	173	0s	-0.001339286	
151	173	60s	29.40401786	41.39330357
151	173	300s	33.20223214	44.75491071
174	184	0s	-0.047619048	
174	184	60s	19.76587302	46.82142857
174	184	300s	19.84920635	50.82936508
176	184	0s	0.045918367	
176	184	60s	56.10204082	55.51530612
176	184	300s	59.97959184	62.33163265
177	184	0s	-0.029761905	
177	184	60s	51.33333333	53.20833333
177	184	300s	57.14285714	57.67857143
185	197	0s	-0.079545455	
185	197	60s	61.68668831	62.65097403
185	197	300s	70.35227273	73.60227273
188	200	0s	-0.061688312	
188	200	60s	51.30844156	46.90584416
188	200	300s	61.94805195	62.43831169
189	200	0s	-0.003571429	
189	200	60s	49.42857143	49.03928571
189	200	300s	68.90714286	67.72142857
198	220	0s	0.015306122	
198	220	60s	46.1547619	49.71938776
198	220	300s	53.34183673	52.67517007
201	214	0s	-0.025297619	
201	214	60s	63.90922619	67.96875
201	214	300s	66.11160714	72.01339286
201	218	0s	-0.006696429	
201	218	60s	56.14955357	61.65401786
201	218	300s	54.50223214	61.515625
212	230	0s	-0.008403361	
212	230	60s	35.58403361	35.26470588
212	230	300s	36.33193277	33.71848739
215	230	0s	0.058673469	
215	230	60s	47.35204082	45.3877551
215	230	300s	49.45663265	44.67346939
231	250	0s	-0.023109244	
231	250	60s	30.29831933	34.21218487
231	250	300s	33.28781513	37.04411765

239	250	0s	0.051785714	
239	250	60s	37.38035714	35.52678571
239	250	300s	41.32321429	41.31607143
251	263	0s	-0.008116883	
251	263	60s	35.13149351	36.10551948
251	263	300s	37.76136364	38.35227273
251	266	0s	0.151147959	
251	266	60s	8.59247449	13.8692602
251	266	300s	7.59375	12.58737245
253	263	0s	-0.025793651	
253	263	60s	47.0734127	47.56150794
253	263	300s	50.2281746	49.24801587
267	277	0s	0.001984127	
267	277	60s	73.83531746	66.03769841
267	277	300s	73.6765873	68.88293651
267	284	0s	0.044642857	
267	284	60s	41.10044643	40.28348214
267	284	300s	52.38616071	48.55803571
279	288	0s	0	
279	288	60s	52.02232143	39.41517857
279	288	300s	61.875	59.28571429
285	302	0s	-0.042857143	
285	302	60s	4.382142857	10.35357143
285	302	300s	4.507142857	11.45
289	302	0s	-0.012175325	
289	302	60s	4.492694805	11.78814935
289	302	300s	5.320616883	13.38068182
290	302	0s	-0.064285714	
290	302	60s	10.57142857	13.71428571
290	302	300s	13.33214286	15.78214286
292	302	0s	-0.073660714	
292	302	60s	7.854910714	14.40401786
292	302	300s	11.41294643	16.71651786
303	309	0s	-0.914285714	
303	309	60s	4.892857143	7.7
303	309	300s	5.328571429	8.178571429
304	311	0s	0.101190476	
304	311	60s	13.7202381	13.625
304	311	300s	17.39285714	16.9702381
316	330	0s	0.058035714	
316	330	60s	6.180059524	12.98363095
316	330	300s	6.441964286	13.69494048
317	330	0s	-0.006493506	
317	330	60s	16.45779221	26.17857143

317	330	300s	16.47402597	27.09415584
319	327	0s	0.168367347	
319	327	60s	64.80102041	54.48469388
319	327	300s	70.8877551	67.20408163
331	345	0s	0.057692308	
331	345	60s	74.32417582	73.0260989
331	345	300s	78.93131868	77.43131868
331	357	0s	-0.053571429	
331	357	60s	8.718167702	23.25232919
331	357	300s	9.666149068	23.83462733
333	345	0s	-0.073051948	
333	345	60s	6.76461039	77.625
333	345	300s	16.08116883	83.18668831
333	356	0s	-0.005357143	
333	356	60s	21.17678571	27.95357143
333	356	300s	27.64017857	29.09196429
335	356	0s	-0.063492063	
335	356	60s	29.66269841	30.55555556
335	356	300s	32.12301587	32.87698413
342	356	0s	-0.185064935	
342	356	60s	50.11688312	51.01298701
342	356	300s	50.94480519	50.50649351
344	357	0s	-0.108928571	
344	357	60s	28.4625	42.07321429
344	357	300s	34.6875	45.15178571
345	356	0s	-0.058035714	
345	356	60s	62.27678571	61.90178571
345	356	300s	60.12946429	60.94196429
346	357	0s	-0.073660714	
346	357	60s	35.35491071	43.41294643
346	357	300s	41.91294643	48.05133929
348	357	0s	0.028061224	
348	357	60s	66.50255102	65.83418367
348	357	300s	65.38520408	65.67602041
359	377	0s	-0.127232143	
359	377	60s	32.81919643	48.96428571
359	377	300s	35.91964286	44.12276786
361	375	0s	0.005952381	
361	375	60s	44.47916667	47.9375
361	375	300s	49.93154762	52.78571429
**362**	**375**	**0s**	**0.002435065**	
**362**	**375**	**60s**	**80.56899351**	**44.98295455**
**362**	**375**	**300s**	**73.79464286**	**56.04788961**
362	377	0s	0.059065934	

362	377	60s	18.84752747	16.90521978
362	377	300s	20.26236264	21.9739011
363	377	0s	0.022321429	
363	377	60s	45.13839286	46.75446429
363	377	300s	50.49107143	52.63392857
397	405	0s	0.051020408	
397	405	60s	41.87755102	47.60204082
397	405	300s	52.20408163	54.83163265
404	410	0s	0	
404	410	60s	89.28571429	83.01428571
404	410	300s	98.72142857	95.30714286
418	438	0s	-0.028195489	
418	438	60s	30.59210526	33.56109023
418	438	300s	41.10902256	39.73308271
418	439	0s	-0.010714286	
418	439	60s	10.84642857	13.78571429
418	439	300s	10.73571429	16.97142857
427	438	0s	0.05	
427	438	60s	33.82142857	24.52142857
427	438	300s	32.09285714	31.425
427	439	0s	-0.00974026	
427	439	60s	14.81168831	18.99025974
427	439	300s	12.72402597	24.80844156
**428**	**439**	**0s**	**0.073214286**	
**428**	**439**	**60s**	**115.5303571**	**69.20535714**
**428**	**439**	**300s**	**102.1267857**	**81.7875**
429	439	0s	-0.128968254	
429	439	60s	20.91865079	20.72420635
429	439	300s	18.74007937	28.54960317
430	438	0s	0.056122449	
430	438	60s	35.09693878	32.38265306
430	438	300s	39.61734694	38.1377551
431	439	0s	-0.045918367	
431	439	60s	22.19387755	29.23469388
431	439	300s	19.41326531	35.06632653
432	439	0s	-0.047619048	
432	439	60s	23.48214286	30.98214286
432	439	300s	25.33333333	39.47619048
439	448	0s	0.060267857	
439	448	60s	33.61383929	42.06026786
439	448	300s	35.87276786	41.01116071
444	451	0s	0.232142857	
444	451	60s	186.0952381	114.8690476
444	451	300s	186.3214286	115.8571429

448	455	0s	0.151785714	
448	455	60s	58.66369048	75.52678571
448	455	300s	60.0327381	70.0625
457	469	0s	-0.076298701	
457	469	60s	42.29383117	49.69318182
457	469	300s	42.15097403	44.49188312
458	466	0s	-0.040816327	
458	466	60s	76.9744898	78.3622449
458	466	300s	78.70408163	77.95408163
458	467	0s	0.129464286	
458	467	60s	51.42857143	57.51785714
458	467	300s	52.71875	56.58035714

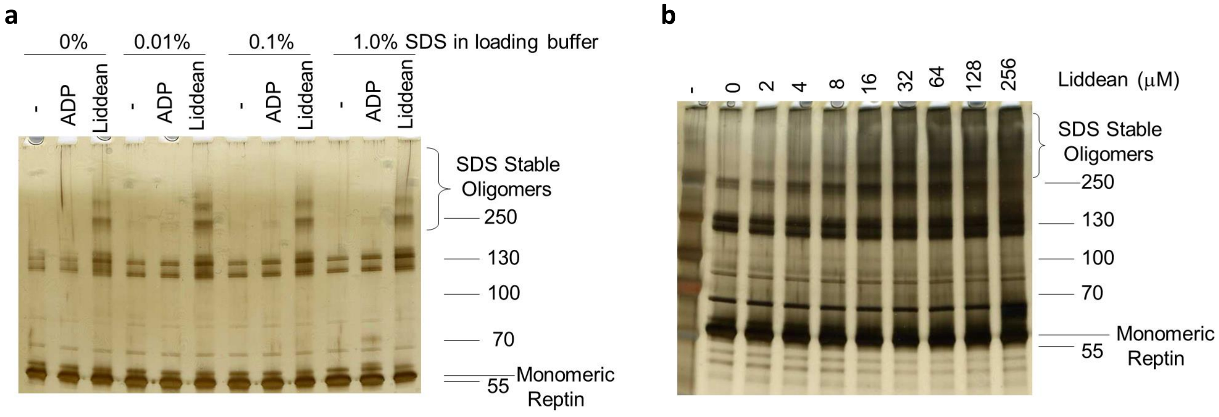

Fig. S8. The effects of Liddean on the oligomerization dynamics of reptin. (**a**). Reptin (1 μg) was subjected to denaturing SDS (0.1%) gel electrophoresis in ADP binding buffer without or with ADP (100 μM) or Liddean (100 μM), as indicated. After 30 minutes of incubation at room temperature, gel loading buffer was added (with the indicated SDS concentrations from 0 to 1%), and electrophoresis was then carried out. Reptin protein was visualized by silver staining. (**b**). Reptin (1 μg) was subjected to denaturing SDS (0.1%) gel electrophoresis in ADP binding buffer with increasing concentrations of Liddean, as indicated. After 30 minutes of incubation at room temperature, gel loading buffer (0% SDS) was added and electrophoresis was carried out. Reptin protein was visualized by silver staining. This formation of SDS-resistant oligomers correlates with the specific reptin-AGR2 peptide binding activity induced by Liddean (**Fig. S5a**, compound **11**).

2.5 Discovering new Liddean-Stimulated Peptide Docking Motifs on Reptin

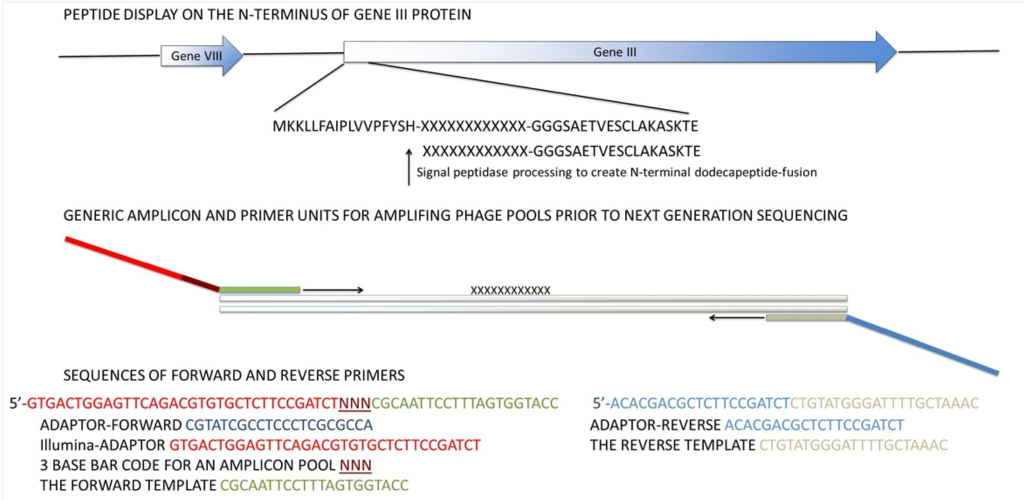

Fig. S9. Discovery of new Liddean-dependent interaction motifs for reptin. Next generation sequencing of peptide-phage pool obtained from a reptin screen in the *apo* and ligand bound state. Reptin without or with ligand was adsorbed onto the solid phase without or with ligands ADP or Liddean. After selection of the peptide library on reptin protein, elution and propagation in bacteria, the phage DNA was amplified using PCR primer sets that capture the sequences flanking the peptide insert (as shown above). Pooling of all phage into deep sequencing reactions can be done with subsequent deconvolution using the “bar code” whose position in the primer is indicated.

Please note: **The raw phage display data is available from the authors on request.**

Primer	Sequence 5'-3'
Forward 00	GTGACTGGAGTTCAGACGTGTGCTCTTCCGATCTAAACGCAATTCCTTTAGTGGTACC
Forward 01	GTGACTGGAGTTCAGACGTGTGCTCTTCCGATCTAATCGCAATTCCTTTAGTGGTACC
Forward 02	GTGACTGGAGTTCAGACGTGTGCTCTTCCGATCTAAGCGCAATTCCTTTAGTGGTACC
Forward 03	GTGACTGGAGTTCAGACGTGTGCTCTTCCGATCTAACCGCAATTCCTTTAGTGGTACC
Forward 04	GTGACTGGAGTTCAGACGTGTGCTCTTCCGATCTATACGCAATTCCTTTAGTGGTACC
Forward 05	GTGACTGGAGTTCAGACGTGTGCTCTTCCGATCTATTCGCAATTCCTTTAGTGGTACC
Forward 06	GTGACTGGAGTTCAGACGTGTGCTCTTCCGATCTATGCGCAATTCCTTTAGTGGTACC
Forward 07	GTGACTGGAGTTCAGACGTGTGCTCTTCCGATCTATCCGCAATTCCTTTAGTGGTACC
Forward 08	GTGACTGGAGTTCAGACGTGTGCTCTTCCGATCTAGACGCAATTCCTTTAGTGGTACC
Forward 09	GTGACTGGAGTTCAGACGTGTGCTCTTCCGATCTAGTCGCAATTCCTTTAGTGGTACC
Forward 10	GTGACTGGAGTTCAGACGTGTGCTCTTCCGATCTAGGCGCAATTCCTTTAGTGGTACC
Forward 11	GTGACTGGAGTTCAGACGTGTGCTCTTCCGATCTAGCCGCAATTCCTTTAGTGGTACC
Forward 12	GTGACTGGAGTTCAGACGTGTGCTCTTCCGATCTACACGCAATTCCTTTAGTGGTACC
Forward 13	GTGACTGGAGTTCAGACGTGTGCTCTTCCGATCTACTCGCAATTCCTTTAGTGGTACC
Forward 14	GTGACTGGAGTTCAGACGTGTGCTCTTCCGATCTACGCGCAATTCCTTTAGTGGTACC
Forward 15	GTGACTGGAGTTCAGACGTGTGCTCTTCCGATCTACCCGCAATTCCTTTAGTGGTACC
Forward 16	GTGACTGGAGTTCAGACGTGTGCTCTTCCGATCTTAACGCAATTCCTTTAGTGGTACC
Reverse	ACACGACGCTCTTCCGATCTCTGTATGGGATTTTGCTAAAC

Table S3. PCR Primer bar codes. PCR was used to amplify phage DNA from each round of screening using the primer bar codes shown here that have an Illumina adaptor sequence and a 3 letter bar code

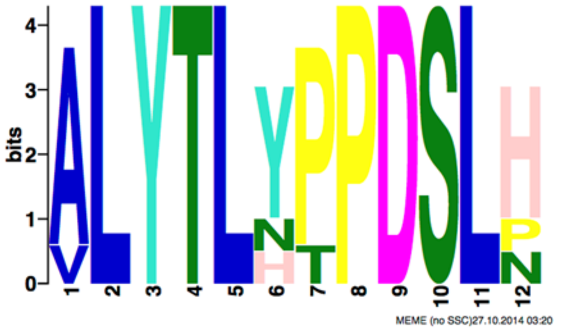

Ref XP 005256701.1 **lethal (2) giant Larvae protein homolog 1**; *Lethal giant larvae 2 regulates development of the ciliated organ Kupffer's vescile*. Development 2013; 140 1550.

```
Query   4      TLHPPDSL
               TLHP DSL
Sbjct 637      TLHPNDSL
```

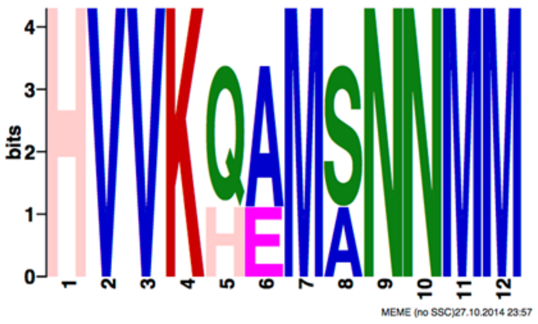

Ref XP 859065.2 **LEBERCILIN** *Mutations in LCA5 encoding the Ciliary protein lebercilin Cause Leber congenital amaurosis*. Nat Genetics 2007; 39 889.

```
Query 1   HVVKQE 6
          HVVKQE
Sbjct 399 HVVKQE 404
```

Fig. S10. Additional ciliopathy proteins present in the list of human proteins which contain consensus sites identified by our Liddean-bound reptin screen. Processing the top 500 peptides from the *apo* and Liddean bound reptin using MEME to identify the top 10 consensus motifs (http://meme.nbcr.net/meme/cgi-bin/meme.cgi) highlights the distinct sets of motifs acquired in the *apo* and ligand bound form. The motifs were processed using MAST or *blastp* to identify targets in the human proteome that have matches to these motifs, some of which are listed as potential ciliopathy targets (**above**).

2.6 Proximity Ligation Assay

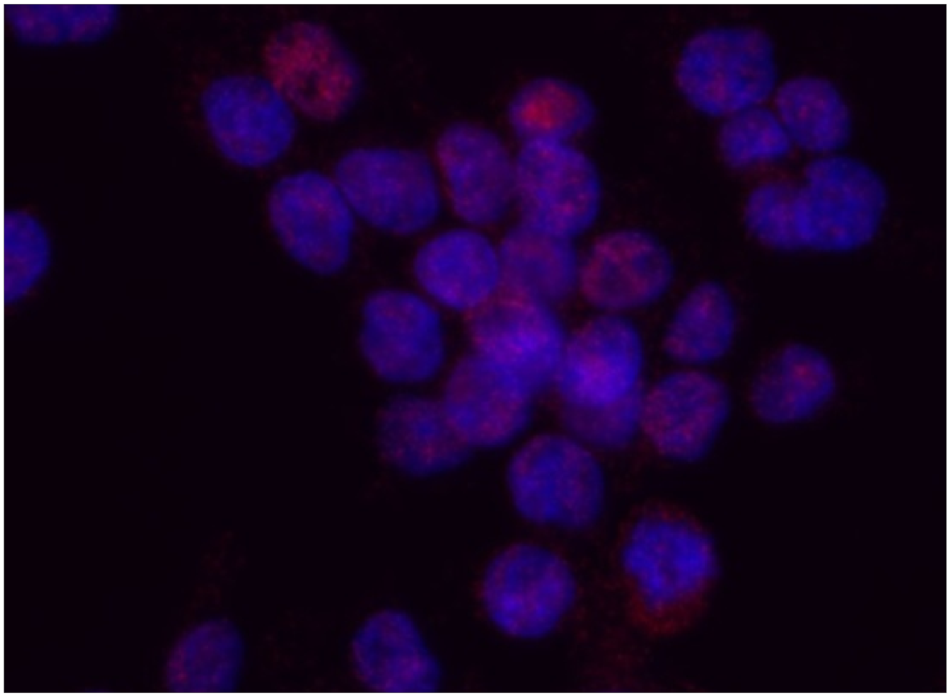

Fig. S11. The effects of Liddean on reptin-pontin protein interactions in HCT116 p53+/+ cells at 2μM. HCT116 cells were processed using the proximity ligation method to identify whether reptin forms a protein-protein interaction in cells and the images are superimposed using DAPI to highlight the nuclear (blue) or cytosolic foci location. The data highlight the foci of reptin-pontin complexes with 2 μM Liddean. The incubation of cells with 2 μM Liddean induced more apparent aggregates and less individual foci, possibly as increased concentrations of Liddean induce the formation of higher-order reptin and pontin complexes in cells.

a

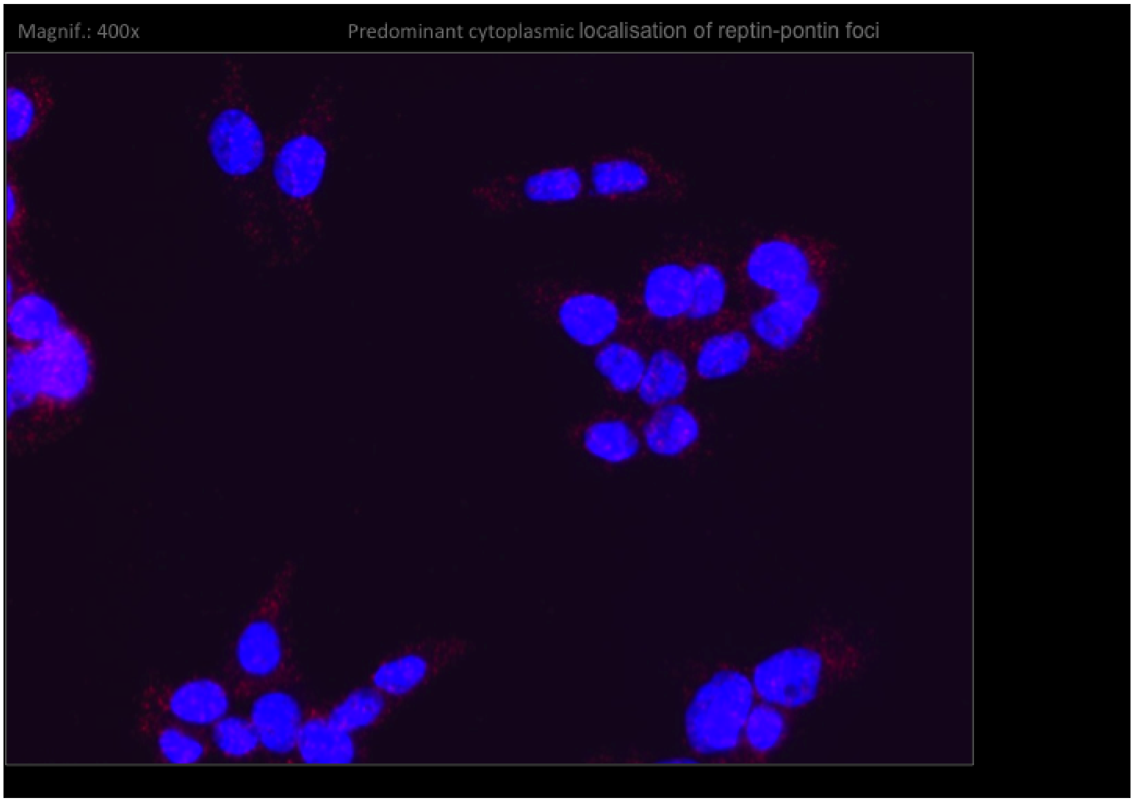
Magnif.: 400x
Predominant cytoplasmic localisation of reptin-pontin foci

b

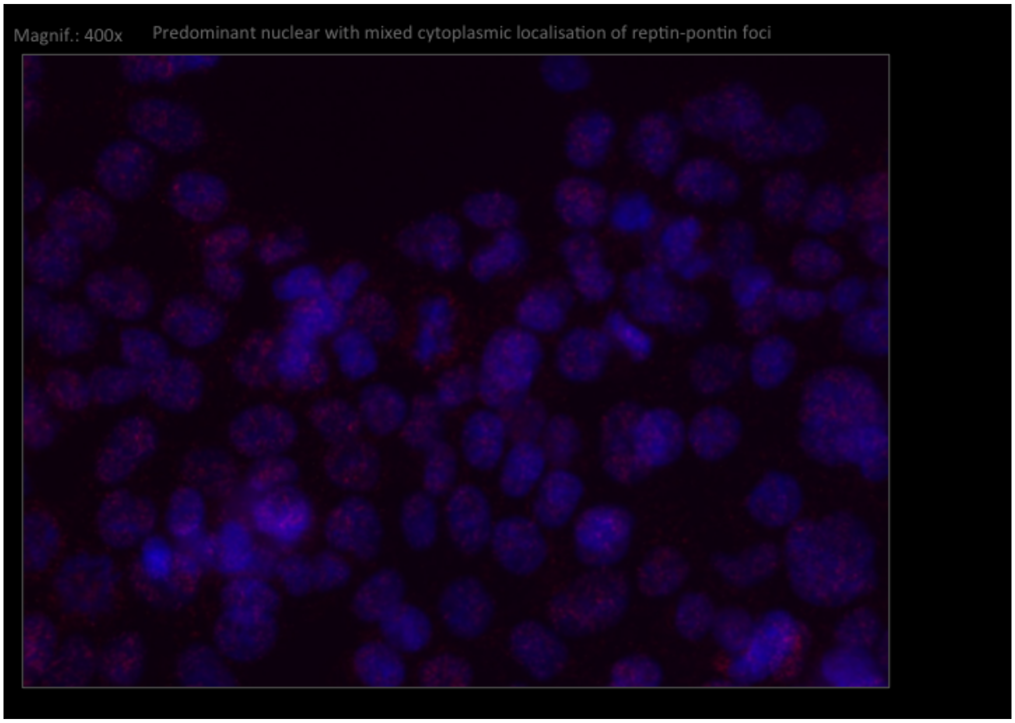
Magnif.: 400x
Predominant nuclear with mixed cytoplasmic localisation of reptin-pontin foci

c

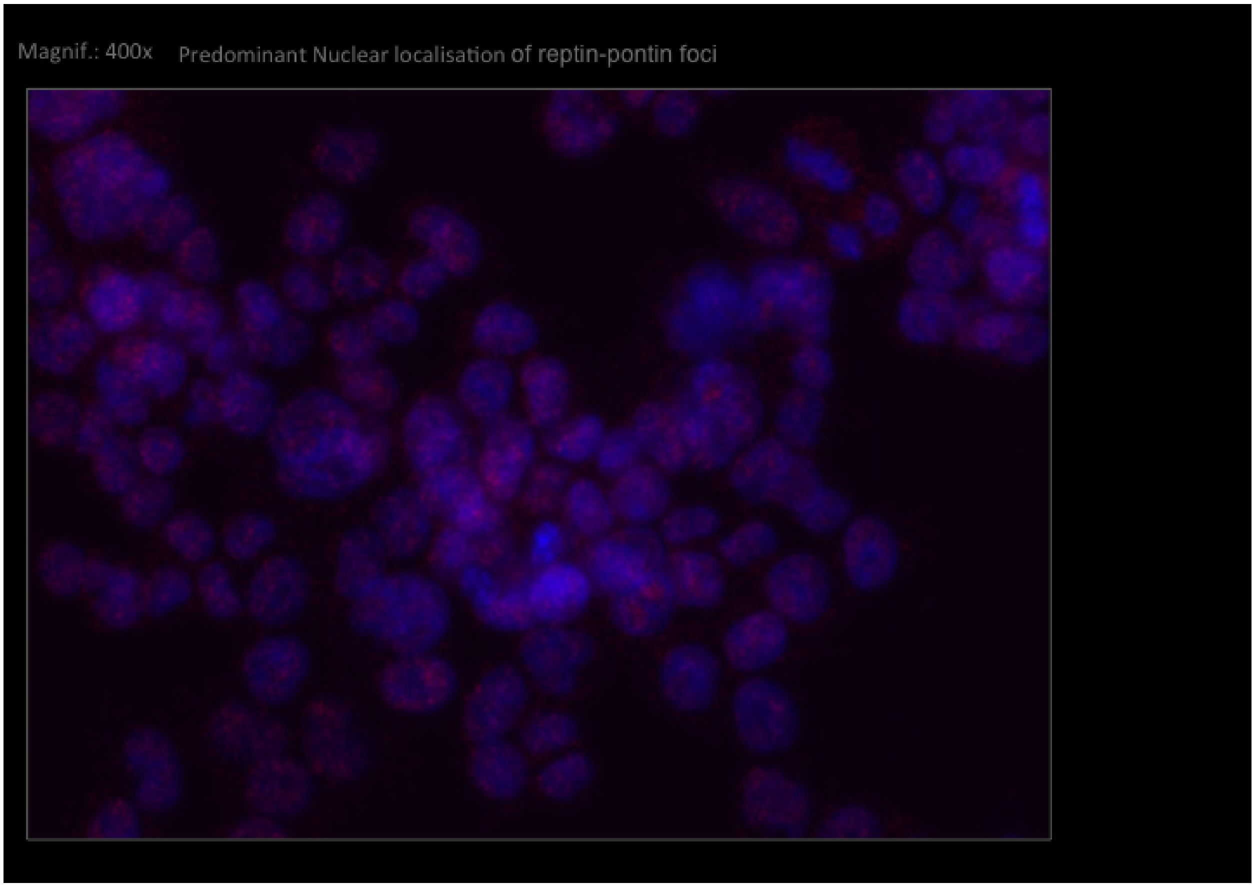

Magnif.: 400x
Predominant Nuclear localisation of reptin-pontin foci

d. Reptin IF without Liddean

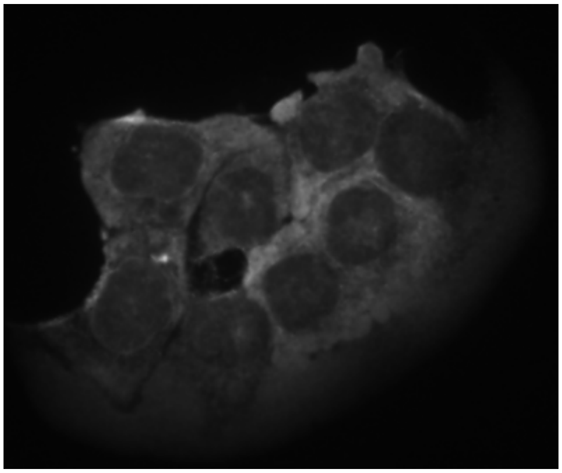

e. Pontin IF without Liddean

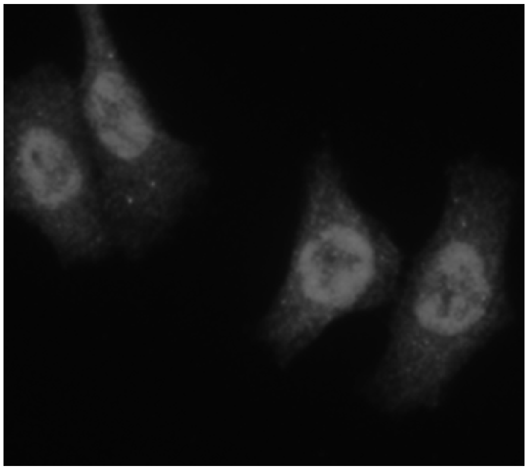

f. Reptin IF with Liddean

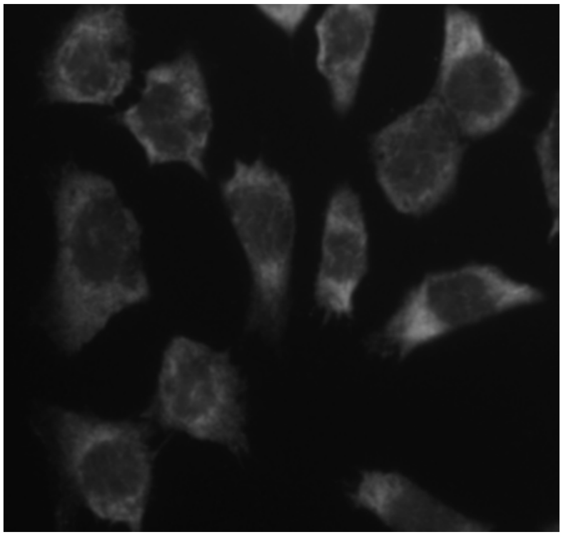

g. Pontin IF with Liddean

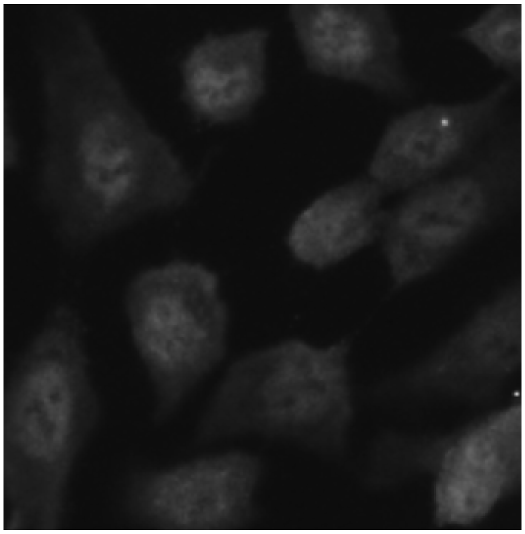

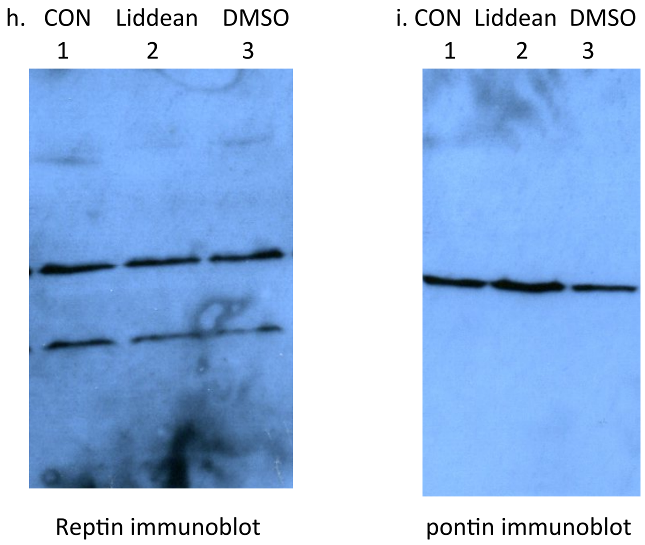

Figure S12. The effects of Liddean on reptin-pontin protein interactions in HCT116 p53-/- cell models using the proximity ligation assay, immunofluorescence and western blotting. HCT116 cells (functionally p53-null) were processed using the proximity ligation method to identify whether reptin forms a protein-protein interaction in cells and the images are superimposed using DAPI to highlight the nuclear (blue) or cytosolic foci location. The data highlight the foci of: (**a**) reptin-pontin complexes in DMSO treated cells; (**b**) reptin-pontin complexes in cells treated with 1 μM Liddean; and (**c**) reptin-pontin complexes with 2 μM Liddean; (d)-(g) Immunofluorescence studies to assess the localization of pontin and reptin in HCT116 cells in the presence or absence of Liddean. In the absence of Liddean, (d) reptin is largely cytosolic and (e) pontin is mixed cytosolic/nuclear; in the presence of Liddean, (f) the majority of reptin is cytosolic and (g) pontin is mixed cytosolic/nuclear. These results are consistent with a situation in which the majority of reptin and pontin are not changing localization on addition of Liddean; (h)-(i) western blot without fractionation from urea based lysis buffers.

2.7 Purification and thermal shift analysis of the binding of ADP to wt-reptin.

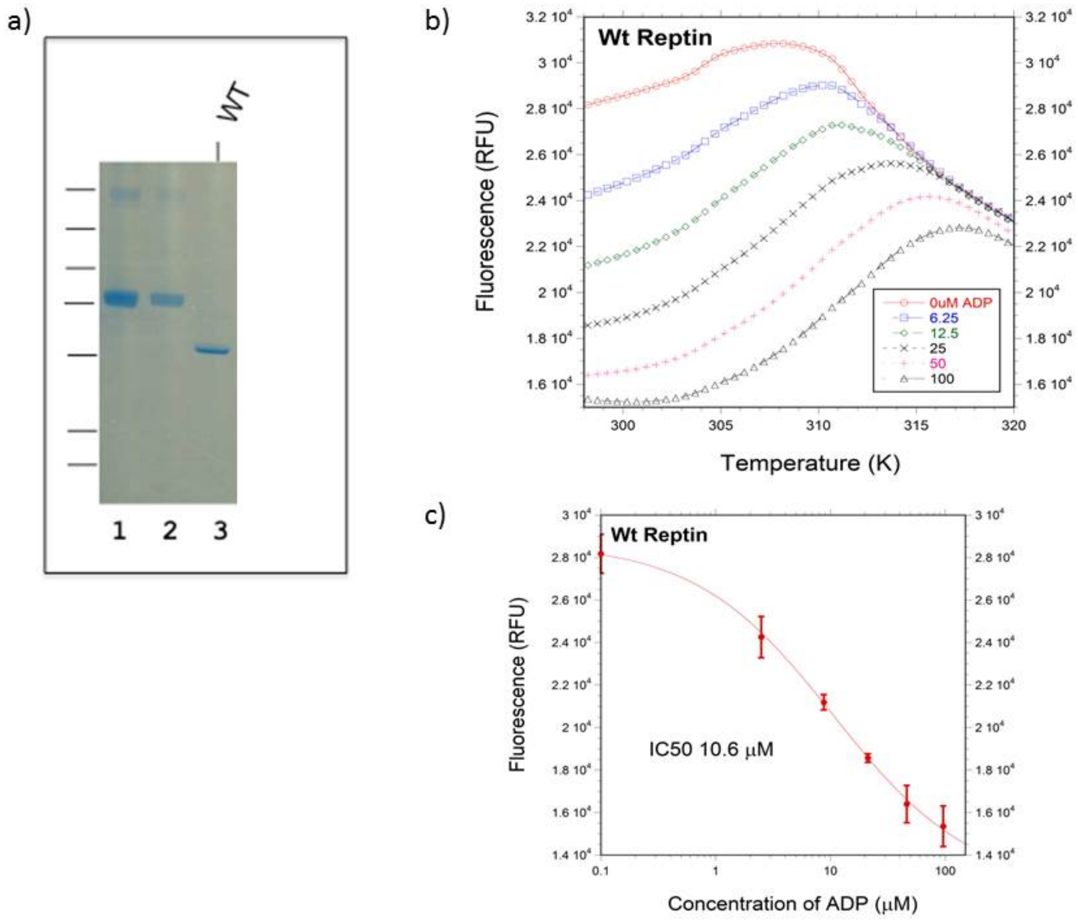

Fig. S13. Purification and thermal shift analysis of the binding of ADP to wt-reptin. (**a**) Purification of biochemically active Reptin. Coomassie blue stained SDS-acrylamide gel of wt-reptin; lanes 1 and 2 are GST fusions of the glutathione-affinity purified recombinant protein; lane 3 is the precision protease eluted forms of reptin protein with the GST tag removed. (**b-c**) The effects of ADP on the thermostability of reptin. WT reptin was incubated without or with increasing concentrations of ligand (from 0-100 µM) and subjected to thermal denaturation in the presence of SyproB to measure unfolding transitions, as published previously. (**b** & **c**) ADP binds wt-reptin with low micromolar affinity. ADP binds to wt-reptin and increases thermal stability. The displacement of SYPRO orange from the protein by ADP is represented by the decrease in background fluorescence prior to the onset of the protein melting. (**b**) Background fluorescence at 298K as a function of ADP concentration (25°C). 7.5 µM wt-reptin was titrated against 0 to 100 µM ADP in 50 mM Tris–HCl, pH 8.0; 120 mM NaCl; 1 mM EDTA and 1 mM DTT. The solid line represents the best fit of the data to F= (Fmax-Fmin)/(1+10^(logIC50-[ADP])*Hillslope). Experiments were repeated in triplicate, mean values are reported and error bars show standard deviations. The melting curve of hexameric wt-reptin does not approximate to a two-state unfolding model when in the *apo* form. There is a very clear trend to increased thermal stability with increasing

concentrations of ADP. The end-point of melting for 7.5 μM changes increases from 34.5 to 44 °C with addition of 100 μM ADP. It was not possible to assign a single mid-point melting temperature from the first derivative of the melting curve for the apo protein and for data collected with low concentrations of ADP or fit a two-state unfolding model. At the highest concentration of ADP (100 μM) the melting curve is a much better approximation to a two-state unfolding model. It is very likely that both the total enthalpy of unfolding, ΔH_u, and change in specific heat capacity on unfolding, ΔC_p, is highly dependent on ADP occupancy across wt-reptin monomers. Unfolding is likely to be more complex and less cooperative when there is less than full occupancy. Sypro orange is an environmentally sensitive dye that associates with hydrophobic pockets of folded proteins. In our data there is a good correlation between background fluorescence before the onset of melting and the end of melting temperature at the different ADP concentrations. This can be explained by SYPRO orange associated with the native protein being displaced by ADP binding. SYPRO orange therefore acts as a fluorescent reporter ligand with low affinity for wt-reptin. (**c**) By plotting background fluorescence before the start of the melt (298K) against concentration of ADP we were able to derive an IC_{50} (Kaleidagraph V4.5.0). The IC_{50} of ADP for Wt-Reptin is 10.6±5.6 μM.

3 Virtual Screening Materials and Methods

3.1 Virtual Library Collation

The screening compound stock lists in SDF format of ChemBridge, Asinex, Maybridge, Enamine, LifeChemicals, Specs, InterBioScreen, ChemDiv and KeyOrganics were merged. Salts were stripped out using Sieve 3.1.0[3], and duplicates removed using canonical SMILES string comparison via Open Babel 2.3.1. The supplied 2D coordinates were converted into 3D using Concord 4.08[4]. The virtual library was then filtered according to Oprea lead-like rules (H-bond acceptors ≤ 9; H-bond donors ≤ 5; MW ≤460; cLogP ≥ -4.6 ≤ 4.2; cLogS ≥ -5; Number of rings ≤ 4; Number of rotatable bonds ≤ 9[5]). This left 1,137,587 molecules. A multiconformer version of this virtual library was produced using Multiconf-DOCK[6]; an average of approximately 4.25 conformers per compound were generated depending on flexibility; this resulted in a virtual library containing a total of 4,840,093 conformers.

3.2 Virtual screening workflow

The rigid-body docking program LIDAEUS[1] was used to dock the conformer virtual library of 4,480,093 molecules into the active site pocket of the pontin/reptin crystal structures. The results were ranked according to LIDAEUS score, the top 49,971 compounds from this list were then docked using Vina. Docked poses were scored using both Vina's internal scoring algorithm and X-Score 1.2[7]; these scores were used via a "rank-by-rank" consensus scheme[8] to create a ranked list. The top 4,974 compounds

were then docked using Autodock. The binding poses predicted by Autodock and Vina were compared via RMSD (using a previously described calculation method that is able to correctly compare symmetrical moieties[9]). Predicted binding poses that were similar (RMSD ≤ 2.0 Å) were also scored using DrugScore 1.2[10]. A final ranked list was prepared via a rank-by-rank scheme, taking the Vina, Autodock, X-Score and DrugScore rank positions into account. The binding mode of the top 100 hits were analyzed by visual inspection and subdivided into different classes based on structural similarity and representative examples of each class were purchased for testing (30 compounds in total).

The Multiconformer input parameters used in the virtual screening are shown below:

limit_max_ligands	no
write_conformations	yes
initial_skip	0
max_conformations	10
calculate_rmsd	yes
use_rmsd_reference_mol	no
rmsd_window	1.15
energy_window	25.0
min_anchor_size	4
number_confs_for_next_growth	1
use_internal_energy	yes
internal_energy_att_exp	6
internal_energy_rep_exp	12
internal_energy_dielectric	4
atom_model	all
vdw_defn_file	Multiconf-DOCK/parameters/vdw_AMBER_parm99.defn
flex_defn_file	Multiconf-DOCK/parameters/flex_Multiconf-DOCK.defn
flex_drive_file	Multiconf-DOCK/parameters/flex_drive_Multiconf-DOCK.tbl
rmsd_reference_filename	na
max_ligands	9999999999999

3.3 Redocking Control Experiments

The initial receptor structure used in redocking experiments was PDB 2XSZ. This structure was chosen because it was the highest resolution ligand-containing structure of Reptin available. The same protonation state was used throughout the whole process. The ADP ligand present in the protein crystal structure was redocked to verify that the docking programs to be used for the virtual screening were successfully able to correctly predict its binding conformation. Water molecules and other hetero atoms were removed and the program PDB2PQR 1.8[11] was used to assign position-optimised hydrogen atoms, utilising the additional PropKa[12] algorithm with a pH of 7.4 to predict protonation states. The MGLTools

1.5.4 utility prepare_receptor4.py was used to assign Gasteiger charges to atoms. Hydrogen atoms were assigned to ligand structures using OpenBabel 2.3.1[13], utilising the -p option to predict the protonation states of functional groups at pH 7.4. The MGLTools utility prepare_ligand4.py was used to assign Gasteiger charges and rotatable bonds. As Vina 1.1.2[14] and Autodock 4.2.3[15] both use the same pdbqt format for their input, the same prepared files could be used for each. A grid box that encompassed the maximum dimensions of the ligand plus 12 Å in each direction was used. The starting translation and orientation of the ligand and the torsion angles of all rotatable bonds were set to random. The Autogrid grid point spacing was set at 0.2 Å. The Autodock parameter file specified 10 Lamarckian genetic algorithm runs, 2,000,000 energy evaluations and a population size of 300. Each docking program was used to automatically dock the ligand back into the pontin binding pocket and the results compared with the crystallographically determined binding mode via RMSD.

4 Synthesis and Characterization of Compounds

4.1 General Considerations

Chemicals and reagents were obtained from either Aldrich or Alfa-Aesar and were used as received unless otherwise stated. All reactions involving moisture sensitive reagents were performed in oven dried glassware under positive pressure of nitrogen. Dichloromethane (DCM) was obtained dry from a solvent purification system (MBraun, SPS-800).

Thin-layer chromatography was performed using glass plates coated with silica gel (with fluorescent indicator UV_{254}) (Aldrich). Developed plates were air dried and analyzed under a UV lamp, Model UVGL-58 (Mineralight LAMP, Multiband $UV_{254/365}$ nm) and where necessary stained with potassium permanganate to aid identification.

Melting points were recorded in open capillaries using an Electrothermal 9100 melting point apparatus. Values are quoted to the nearest 1 °C and are uncorrected.

^{1}H-NMR spectra were measured at room temperature (298 K) on a Bruker DPX 400 (^{1}H = 400 MHz), Bruker Avance 300 (^{1}H = 300.1 MHz) and Bruker Avance 500 (^{1}H = 500 MHz) instruments. Deuterated solvents were used and ^{1}H NMR chemical shifts were internally referred to $CHCl_3$ (7.26 ppm) in chloroform-d_1 solution and to $CD_2HSO_2CD_3$ (2.50 ppm) in dimethylsulfoxide-d_6 solution. Chemical shifts are expressed as δ in unit of ppm.

^{13}C-NMR spectra were recorded in the same conditions and in the same solvents using the DEPTQ pulse sequence mode on a Bruker DPX 400 (^{13}C = 101 MHz), Bruker Advance 300 (^{13}C = 75 MHz) and a Bruker Advance 500 (^{13}C = 126 MHz). Data processing was carried out using MestReNova NMR (Mestrelab). In ^{1}H NMR, the multiplicity used for assignment is indicated by the following abbreviations: s = singlet, d = doublet, dd = doublet of doublets, dt = doublet of triplets, t = triplet, q = quadruplet, m = multiplet, br = broad. Signals of protons and carbons were assigned, as far as possible, by using the following two-dimensional NMR spectroscopy techniques: [^{1}H, ^{1}H] COSY, [^{1}H, ^{13}C] COSY (HSQC: Heteronuclear Single Quantum Coherence) and long range [^{1}H, ^{13}C] COSY (HMBC: Heteronuclear Multiple Bond Connectivity). ^{1}H NMR were supplied by Chembridge for the selected purchased analogues.

Mass spectrometry (electrospray mode, ES) were recorded on a high performance orthogonal acceleration reflecting TOF mass spectrometer operating in positive and negative mode, coupled to a Waters 2975 HPLC.

Fourier Transform infra-red spectra (FT IR) were acquired on a Perkin Elmer paragon 1000 FT spectrophometer or a Shimadzu IRAffinity-1 FT spectrophotometer with a Pike MIRacle™. Absorption maxima are reported in wavenumbers (cm^{-1}).

4.2 General Procedures

General Method A

Synthesis of the substituted oxazole system:

To polyphosphoric acid (~20 eq.) at 120 °C were added simultaneously the appropriate aminophenol (1 eq.) and a range of substituted benzoic acids (1 eq.). The resulting mixture was heated to 200 °C for 4 h. After cooling slightly, ice water was added to the reaction. The solution was neutralized by the slow addition of a saturated sodium hydrogen carbonate solution and the resulting precipitate was collected by filtration. Drying in a vacuum oven yielded the pure product.

General Method B

Formation of the amide bond:

To a solution of the appropriate aniline (1 eq.) in DCM (1 mL/mmol) was added a range of carbonyl chlorides (2.5 eq.). The mixture was stirred for 10 minutes before addition of DIPEA (1.5 eq.) dropwise. The reaction was stirred at room temperature for 24-72 hours. The resulting precipitate was filtered and dried to yield the desired compound. Recrystallization from DCM afforded the product in high purity.

4.3 Experimental procedures

2-Chloro-5-(oxazolo[4,5-b]pyridin-2-yl)aniline (S40)

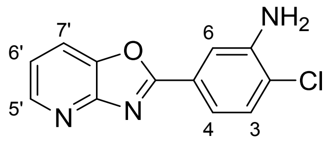

Prepared from 2-amino-3-hydroxypyridine (4.0 g, 36.3 mmol) using general method A. **S40** was recovered as a white solid (4.9 g, 55%). **mp** 205-208 °C; **IR** (solid) ν $_{max}$: 3453 (NH), 3345 (NH), 1614, 1406, 1261, 781 (C-Cl); **^{1}H NMR** (500 MHz, Chloroform-*d*) δ 8.57 (dd, *J* = 4.9, 1.5 Hz, 1H, C5'-H), 7.84 (dd, *J* = 8.1, 1.5 Hz, 1H, C7'-H), 7.73 (d, *J* = 2.0 Hz, 1H, C6-H), 7.61 (dd, *J* = 8.3, 2.0 Hz, 1H, C4-H), 7.40 (d, *J* = 8.3 Hz, 1H, C3-H), 7.29 (dd, *J* = 8.1, 4.9 Hz, 1H, C6'-H), 4.30 (br s, 2H, NH_2); **^{13}C NMR** (126 MHz, Chloroform-*d*) δ 165.3 (C2'), 156.4 (C4b), 146.9 (C5'), 143.6 (C1), 143.2 (C5b), 130.2 (C3), 125.9 (C5), 123.6 (C2), 120.3 (C6'), 118.4 (C4), 118.3 (C7'), 114.8 (C6); ***m/z*** (ES$^+$) 246.04 ([M+H]$^+$, 100 %); **HRMS** (ES$^+$) Calcd for $C_{12}H_9ON_3Cl$ [M+H]$^+$: 246.0429, found 246.0431. Data is in accordance with the literature[16].

5-Chloro-2-(oxazolo[4,5-b]pyridin-2-yl)aniline (S41)

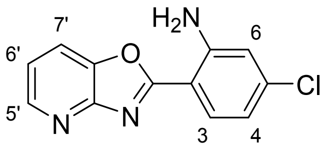

Prepared from 2-amino-3-hydroxypyridine (600 mg, 5.45 mmol) using general method A. **S41** was recovered as white solid (690 mg, 52%). **mp** 256-259 °C; **IR** (solid) ν $_{max}$: 3402 (NH), 3310 (NH), 3201, 1612, 1543, 1481, 1404, 772 (C-Cl); **^{1}H NMR** (500 MHz, DMSO-*d*$_6$) δ 8.51 (d, *J* = 5.2 Hz, 1H, C5'-H), 8.19 (d, *J* = 8.2 Hz, 1H, C7'-H), 7.94 (d, *J* = 8.3 Hz, 1H, C3-H), 7.50 – 7.35 (m, 3H, C6'-H, NH_2), 7.01 (d, *J* = 6.8 Hz, 1H, C6-H), 6.73 (d, *J* = 8.3 Hz, 1H, C4-H); **^{13}C NMR** (126 MHz, DMSO-*d*$_6$) δ 164.2 (C2'), 155.2 (C4b), 150.3 (C1), 146.2 (C5'), 141.1 (C5b), 138.1 (C5), 129.9 (C3), 120.5 (C6'), 118.5 (C7'), 115.6 (C4), 115.1 (C6), 104.6 (C2); ***m/z*** (ES$^+$) 246.04 ([M+H]$^+$, 100%); **HRMS** (ES$^+$) Calcd for $C_{12}H_9ON_3Cl$ [M+H]$^+$: 246.0429, found 246.0430.

3-(Oxazolo[4,5-b]pyridin-2-yl)aniline (S42)

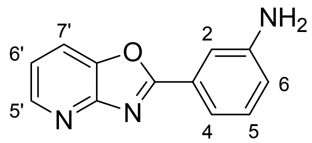

Prepared from 2-amino-3-hydroxypyridine (300 mg, 2.70 mmol) using general method A. **S42** was recovered as an off-white solid (500 mg, 88%). **mp** 186-189 °C; **^{1}H NMR** (300 MHz, DMSO-d$_6$) δ 8.52 (dd, *J* = 4.9, 1.5 Hz, 1H, C5'-H), 8.21 (dd, *J* = 8.1, 1.5 Hz, 1H, C7'-H), 7.48 (t, *J* = 2.0, 1H, C2-H), 7.44 (dd, *J* = 8.1, 4.9 Hz, 1H, C6'-H), 7.38 (ddd, *J* = 7.6, 1.7, 1.1 Hz, 1H, C4-H), 7.26 (t, *J* = 7.8 Hz, 1H, C5-H), 6.84 (ddd, *J* = 8.0, 2.4, 1.1 Hz, 1H, C6-H), 5.55 (br s, 2H, NH_2); **^{13}C NMR** (75 MHz, DMSO-d$_6$) δ 165.6 (C2'), 155.6 (C4b), 149.5 (C1), 146.4 (C5'), 142.6 (C5b), 129.9 (C5), 126.3 (C3), 120.5 (C6'), 118.9 (C7'), 118.1 (C6), 114.9

(C4), 112.3 (C2); ***m/z*** (ES^+) 218.08 ($[M+H]^+$, 100 %); **HRMS** (ES^+) Calcd for $C_{12}H_{10}ON_3$ $[M+H]^+$: 212.0818, found 212.0818. Data is in accordance with the literature[16].

4-(Oxazolo[4,5-b]pyridin-2-yl)aniline (S43)

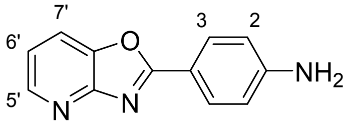

Prepared from 2-amino-3-hydroxypyridine (600 mg, 5.45 mmol) using general method A. **S43** was recovered as pale yellow solid (906 mg, 79%). **mp** 257-259 °C (lit. 252-254 °C[17]); **IR** (solid) ν_{max}: 3395 (NH), 3310 (NH), 3202, 1605, 1489, 1404; **^{1}H NMR** (500 MHz, DMSO-d_6) δ 8.41 (dd, *J* = 4.9, 1.3 Hz, 1H, C5'-H), 8.07 (dd, *J* = 8.1, 1.3 Hz, 1H, C7'-H), 7.91 (d, *J* = 8.6 Hz, 2H, C3-H), 7.31 (dd, *J* = 8.1, 4.9 Hz, 1H, C6'-H), 6.71 (d, *J* = 8.6 Hz, 2H, C2-H), 6.17 (br s, 2H, N*H*$_2$); **^{13}C NMR** (126 MHz, DMSO-d_6) δ 166.0 (C2'), 156.4 (C4b), 153.4 (C1), 145.7 (C5'), 142.3 (C5b), 129.6 (C3), 119.3 (C6'), 117.8 (C7'), 113.5 (C3), 111.7 (C4); ***m/z*** (ES^+) 212.0 ($[M+H]^+$, 100%); **HRMS** (ES^+) Calcd for $C_{12}H_{10}ON_3$ $[M+H]^+$: 212.0818, found 212.0818. Data is in accordance with the literature[17].

2-Fluoro-5-(oxazolo[4,5-b]pyridin-2-yl)aniline (S44)

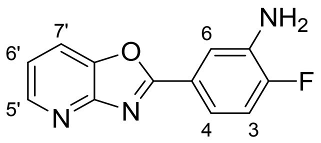

Prepared from 2-amino-3-hydroxypyridine (75 mg, 0.68 mmol) using general method A. **S44** was recovered as an off-white solid (65 mg, 50%). **mp** 214-216 °C; **IR** (solid) ν_{max}: 3464 (NH), 3318 (NH), 3194, 3039, 1613, 1497, 1404, 1173 (C-F); **^{1}H NMR** (400 MHz, DMSO-d_6) δ 8.52 (dd, *J* = 4.9, 1.4 Hz, 1H, C5'-H), 8.21 (dd, *J* = 8.2, 1.4 Hz, 1H, C7'-H), 7.69 (dd, *J* = 8.6, 2.2 Hz, 1H, C6-H), 7.51 – 7.35 (m, 2H, C6'-H, C4-H), 7.25 (dd, *J* = 11.3, 8.4 Hz, 1H, C3-H), 5.65 (br s, 2H, N*H*$_2$); **^{13}C NMR** (101 MHz, DMSO-d_6) δ 164.8 (C2'), 155.6 (C4b), 153.1 (d, *J* = 245.3 Hz, C2), 146.4 (C5'), 142.7 (C5b), 137.5 (d, *J* = 13.8 Hz, C1), 122.4 (d, *J* = 2.7 Hz, C5), 120.53 (C6'), 118.9 (C7'), 116.0 (d, *J* = 19.8 Hz, C3), 115.8 (d, *J* = 7.4 Hz, C4), 115.0 (d, *J* = 6.3 Hz, C6); ***m/z*** (ES^+) 230.07 ($[M+H]^+$, 100 %); **HRMS** (ES^+) Calcd for $C_{12}H_9ON_3F$ $[M+H]^+$: 230.0724, found 230.0725.

2-Bromo-5-(oxazolo[4,5-b]pyridin-2-yl)aniline (S45)

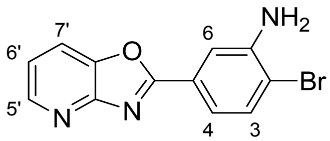

Prepared from 2-amino-3-hydroxypyridine (600 mg, 5.45 mmol) using general method A. **S45** was recovered as a pale brown solid (1.38 g, 87%). **mp** 211-212 °C; **IR** (solid) ν $_{max}$: 3418 (NH), 3287 (NH), 3171, 1736, 1620, 1597, 1543, 1404; **^{1}H NMR** (300 MHz, DMSO-d$_6$) δ 8.54 (dd, *J* = 4.9, 1.4 Hz, 1H, C5'-H), 8.23 (dd, *J* = 8.2, 1.4 Hz, 1H, C7'-H), 7.70 (d, *J* = 2.1 Hz, 1H, C6-H), 7.61 (d, *J* = 8.2 Hz, 1H, C3-H), 7.46 (dd, *J* = 8.2, 4.9 Hz, 1H, C6'-H), 7.31 (dd, *J* = 8.2, 2.1 Hz, 1H, C4-H), 5.79 (br s, 2H, N*H*$_2$); **^{13}C NMR** (75 MHz, DMSO-d$_6$) δ 164.7 (C2'), 155.5 (C4b), 146.7 (C1), 146.5 (C5'), 142.7 (C5b), 133.3 (C3), 125.8 (C5), 120.7 (C6'), 119.0 (C7'), 115.9 (C4), 113.5 (C6), 111.8 (C2); ***m/z*** (ES$^+$) 289.99 ([^{79}BrM+H]$^+$, 100 %), 291.99 ([^{81}BrM+H]$^+$, 100%); **HRMS** (ES$^+$) Calcd for $C_{12}H_9ON_3Br$ [M+H]$^+$: 289.9924, found 289.9927.

3-(oxazolo[4,5-b]pyridin-2-yl)phenol (S46)

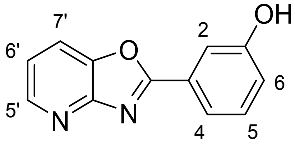

Prepared from 2-amino-3-hydroxypyridine (55 mg, 0.5 mmol) using general method A. **S46** was recovered as an white solid (75 mg, 71%). **mp** 198-201 °C (lit. 203-204 °C[18]); **IR** (solid) ν $_{max}$: 3030 (OH), 2922 (OH), 2603, 1601, 1551, 1449, 1294, 887, 781; **^{1}H NMR** (500 MHz, DMSO-*d*$_6$) δ 10.06 (br s, 1H, O*H*), 8.54 (dd, *J* = 4.9, 1.4 Hz, 1H, C5'-H), 8.24 (dd, *J* = 8.1, 1.4 Hz, 1H, C7'-H), 7.69 (d, *J* = 7.9 Hz, 1H, C4-H), 7.63 (dd, *J* = 2.5, 1.6 Hz, 1H, C2-H), 7.50 – 7.40 (m, 2H, C6'-H, C5-H), 7.07 (ddd, *J* = 8.2, 2.5, 1.0 Hz, 1H, C6-H); **^{13}C NMR** (126 MHz, DMSO-*d*$_6$) δ 164.9 (C2'), 158.0 (C1), 155.5 (C4b), 146.6 (C5'), 142.8 (C5b), 130.7 (C5), 127.0 (C3), 120.8 (C6'), 120.0 (C6), 119.1 (C7'), 118.5 (C4), 114.0 (C2); ***m/z*** (ES$^+$) 213.06 ([M+H]$^+$, 100 %); **HRMS** (ES$^+$) Calcd for $C_{12}H_9O_2N_2$ [M+H]$^+$: 213.0659, found 213.0659. Data is in accordance with the literature[18].

2-Methyl-5-(oxazolo[4,5-b]pyridin-2-yl)aniline (S47)

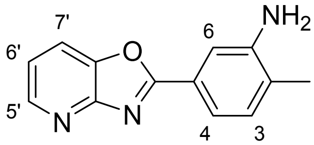

Prepared from 2-amino-3-hydroxypyridine (500 mg, 4.54 mmol) using general method A. **S47** was recovered as an pale brown solid (716 mg, 71%). **mp** 171-173 °C; **IR** (solid) ν $_{max}$: 3426 (NH), 3325 (NH), 3055, 1736, 1620, 1551, 1497, 1404, 1234, 779; **^{1}H NMR** (500 MHz, DMSO-d_6) δ 8.51 (dd, J = 4.9, 1.4 Hz, 1H, C5’-H), 8.19 (dd, J = 8.1, 1.4 Hz, 1H, C7’-H), 7.53 (d, J = 1.8 Hz, 1H, C6-H), 7.42 (dd, J = 8.1, 4.9 Hz, 1H, C6’-H), 7.36 (dd, J = 7.6, 1.8 Hz, 1H, C4-H), 7.17 (d, J = 7.6 Hz, 1H, C3-H), 5.33 (br s, 2H, NH_2), 2.15 (s, 3H, CH_3); **^{13}C NMR** (126 MHz, DMSO-d_6) δ 165.8 (C2’), 155.8 (C4b), 147.5 (C1), 146.3 (C5’), 142.5 (C5b), 130.8 (C3), 126.6 (C2), 124.0 (C5), 120.3 (C6’), 118.7 (C7’), 115.3 (C4), 112.3 (C6), 17.7 (CH_3); ***m/z*** (ES$^+$) 226.09 ([M+H]$^+$, 100 %); **HRMS** (ES$^+$) Calcd for $C_{12}H_{12}ON_3$ [M+H]$^+$: 226.0975, found 226.0974. ^{1}H & ^{13}C NMR not in accordance with literature[16]

4-Amino-2-(oxazolo[4,5-b]pyridin-2-yl)phenol (S48)

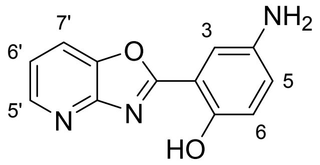

Prepared from 2-amino-3-hydroxypyridine (600 mg, 5.45 mmol) using general method A. **S48** was recovered as dark yellow solid (690 mg, 49%). **mp** 191-193 °C (lit. 181-183 °C[19]); **IR** (solid) ν $_{max}$: 3426 (NH), 3310 (NH), 3202 (OH), 1620, 1535, 1497, 1404, 779; **^{1}H NMR** (500 MHz, DMSO-d_6) δ 10.19 (br s, 1H, OH), 8.55 (dd, J = 4.9, 1.4 Hz, 1H, C5’-H), 8.27 (dd, J = 8.1, 1.4 Hz, 1H, C7’-H), 7.48 (dd, J = 8.1, 4.9 Hz, 1H, C6’-H), 7.27 (d, J = 2.3 Hz, 1H, C3-H), 6.88 (s, 2H, C5-H, C6-H), 5.02 (br s, 2H, NH_2); **^{13}C NMR** (126 MHz, DMSO-d_6) δ 165.2 (C2’), 153.9 (C4b), 149.7 (C1), 146.6 (C5’), 141.8 (C4), 141.4 (C5b), 122.3 (C5), 120.8 (C6’), 119.0 (C7’), 117.9 (C6), 110.4 (C3), 109.5 (C2); ***m/z*** (ES$^+$) 228.07 ([M+H]$^+$, 100%); **HRMS** (ES$^+$) Calcd for $C_{12}H_{10}O_2N_3$ [M+H]$^+$: 228.0768, found 228.0768.

5-(Benzo[d]oxazol-2-yl)-2-bromoaniline (S49)

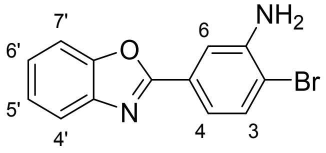

Prepared from 2-aminophenol (545 mg, 5.00 mmol) using general method A. **S49** was recovered as an off-white solid (653 mg, 45%). **mp** 198-200 °C; **IR** (solid) ν $_{max}$: 3449 (NH), 3310 (NH), 3179, 1612, 1543, 1435, 1242, 1065, 741; **^{1}H NMR** (500 MHz, DMSO-d_6) δ 7.81 – 7.75 (m, 2H, C4’-H, C7’-H), 7.66 (d, J = 2.1

Hz, 1H, C6-H), 7.57 (d, *J* = 8.2 Hz, 1H, C3-H), 7.46 – 7.37 (m, 2H, C5'-H, C6'-H), 7.27 (dd, *J* = 8.2, 2.1 Hz, 1H, C4-H), 5.75 (br s, 2H, N*H*$_2$); **^{13}C NMR** (126 MHz, DMSO-*d*$_6$) δ 162.2 (C2'), 150.1 (C4b), 146.6 (C1), 141.5 (C5b), 133.2 (C3), 126.3 (C5), 125.5 (C5'), 124.9 (C6'), 119.8 (C4'), 115.7 (C4), 113.3 (C6), 111.0 (C2), 110.9 (C7'); ***m/z*** (ES$^+$) 288.99 ([^{79}BrM+H]$^+$, 100 %), 290.99 ([^{81}BrM+H]$^+$, 100%); **HRMS** (ES$^+$) Calcd for $C_{13}H_{10}ON_2Br$ [M+H]$^+$: 288.9971, found 288.9975.

***N*-(2-chloro-5-(oxazolo[4,5-b]pyridin-2-yl)phenyl)-[1,1'-biphenyl]-4-carboxamide (1)**

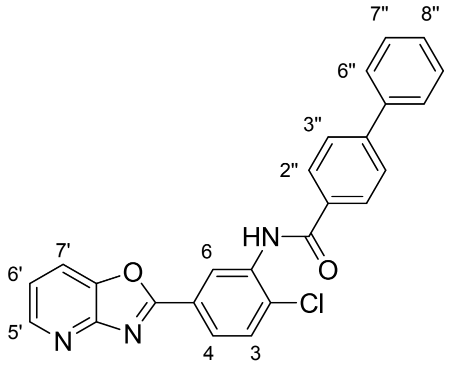

Prepared from **S40** (300 mg, 1.42 mmol) using general method B. **1** recovered after recrystallization (DCM) as a white solid (346 mg, 57%). **mp** 254-256 °C; **IR** (KBr) ν $_{max}$: 3416 (NH), 3275, 3059, 3033, 1653 (C=O); **^{1}H NMR** (400 MHz, DMSO-*d*$_6$) δ 10.39 (br s, 1H, N*H*), 8.61 – 8.53 (m, 2H, C5'-H, C6-H), 8.29 (dd, *J* = 8.2, 1.5 Hz, 1H, C7'-H), 8.19 – 8.11 (m, 3H, C4-H, 2 × C2''-H), 7.93 – 7.83 (m, 3H, C3-H, 2 × C3''-H), 7.82 – 7.75 (m, 2H, 2 × C6''-H), 7.57 – 7.46 (m, 3H, C6'-H, 2 × C7''-H), 7.49 – 7.39 (m, 1H, C8''-H); **^{13}C NMR** (101 MHz, DMSO-*d*$_6$) δ 165.3 (CO), 163.7 (C2'), 155.4 (C4b), 146.8 (C5'), 143.6 (C4''), 143.0 (C5b), 139.0 (C5''), 136.2 (C1), 133.4 (C2), 132.5 (C1''), 131.0 (C3), 129.1 (2 × C7''), 128.6 (2 × C2''), 128.3 (C8''), 127.0 (2 × C6''), 126.8 (2 × C3''), 126.6 (C6), 126.1 (C4), 125.2 (C5), 121.1 (C6'), 119.3 (C7'); ***m/z*** (ES$^-$) 423.82 ([M-H]$^-$, 100 %); **HRMS** (ES$^-$) Calcd for $C_{25}H_{15}ClN_3O_2$ [M-H]$^-$: 424.0839, found 424.0853.

***N*-(5-chloro-2-(oxazolo[4,5-b]pyridin-2-yl)phenyl)-[1,1'-biphenyl]-4-carboxamide (7)**

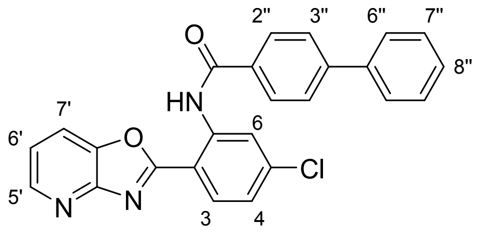

Prepared from **S41** (550 mg, 2.6 mmol) using the general method B. **7** recovered after recrystallization (DCM) as a pale yellow solid (185 mg, 17%). **mp** 254-256 °C **IR** (KBr) ν $_{max}$: 3408 (NH), 3072, 3032, 1684

(C=O), 1590; **^{1}H NMR** (500 MHz, Chloroform-*d*) δ 12.64 (br s, 1H, N*H*), 9.21 (d, *J* = 2.0 Hz, 1H, C6-H), 8.63 (dd, *J* = 4.9, 1.4 Hz, 1H, C5'-H), 8.36 (d, *J* = 8.3 Hz, 2H, 2 × C2''-H), 8.20 (d, *J* = 8.5 Hz, 1H, C3-H), 7.92 (dd, *J* = 8.1, 1.4 Hz, 1H, C7'-H), 7.86 (d, *J* = 8.3 Hz, 2H, 2 × C3''-H), 7.73 – 7.66 (m, 2H, 2 × C6''-H), 7.49 (dd, *J* = 8.3, 6.8 Hz, 2H, 2 × C7''-H), 7.44 – 7.39 (m, 1H, C8''-H), 7.38 (dd, *J* = 8.1, 4.9 Hz, 1H, C6'-H), 7.23 (dd, *J* = 8.5, 2.0 Hz, 1H, C4-H); **^{13}C NMR** (126 MHz, Chloroform-*d*) δ 166.1 (CO), 164.0 (C2'), 154.9 (C4b), 147.3 (C5'), 145.2 (C4''), 141.8 (C5b), 141.0 (C5), 140.6 (C1), 140.1 (C5''), 133.0 (C1''), 129.4 (C3), 129.0 (2 × C7''), 128.5 (2 × C2''), 128.2 (C8''), 127.8 (2 × C3''), 127.4 (2 × C6''), 123.6 (C4), 121.0 (C6'), 120.9 (C6), 118.6 (C7'), 110.8 (C2); ***m/z*** (ES$^+$) 447.86 [M+Na]$^+$; **HRMS** (ES$^+$) Calcd for $C_{25}H_{16}ClN_3O_2Na$ [M+Na]$^+$: 448.0829, found 448.0826.

***N*-(3-(oxazolo[4,5-b]pyridin-2-yl)phenyl)-[1,1'-biphenyl]-4-carboxamide (8)**

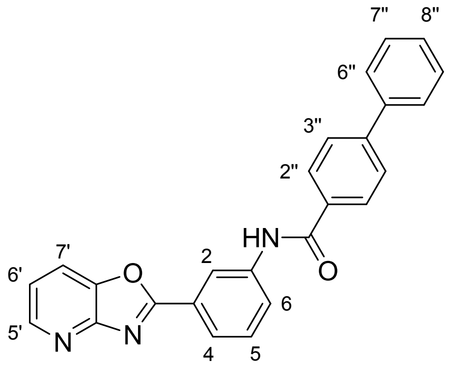

Prepared from **S42** (150 mg, 0.71 mmol) using the general method B. **8** recovered after recrystallization (DCM) as a white solid (33 mg, 12%). **mp** 261-264 °C; **IR** (KBr) ν $_{max}$: 3440 (NH), 2962, 2924, 1649 (C=O), 1527; **^{1}H NMR** (300 MHz, DMSO-d$_6$) δ 10.63 (br s, 1H, N*H*), 8.87 (t, *J* = 1.9 Hz, 1H, C2-H), 8.56 (dd, *J* = 4.9, 1.4 Hz, 1H, C5'-H), 8.27 (dd, *J* = 8.2, 1.4 Hz, 1H, C7'-H), 8.19 – 8.06 (m, 3H, C6-H, 2 × C2''-H), 8.04 – 7.93 (m, 1H, C4-H), 7.92 – 7.82 (m, 2H, 2 × C3''-H), 7.82 – 7.72 (m, 2H, 2 × C6''-H), 7.64 (t, *J* = 8.0 Hz, 1H, C5-H), 7.57 – 7.36 (m, 4H, C6'-H, C8''-H, 2 × C7''-H); **^{13}C NMR** (75 MHz, DMSO-d$_6$) δ 165.5 (CO), 164.8 (C2'), 155.5 (C4b), 146.6 (C5'), 143.4 (C4''), 142.8 (C5b), 140.2 (C1), 139.1 (C5''), 133.3 (C1''), 129.9 (C5), 129.1 (2 × C7''), 128.5 (2 × C2''), 128.2 (C8''), 127.0 (2 × C6''), 126.7 (2 × C3''), 126.3 (C3), 124.1 (C6), 122.8 (C4), 120.9 (C6'), 119.1 (CH), 119.0 (CH); ***m/z*** (ES$^-$) 389.84 ([M-H]$^-$, 100 %); **HRMS** (ES$^-$) Calcd for $C_{25}H_{16}N_3O_2$ [M-H]$^-$: 390.1245, found 390.1243.

***N*-(4-(oxazolo[4,5-b]pyridin-2-yl)phenyl)-[1,1'-biphenyl]-4-carboxamide (9)**

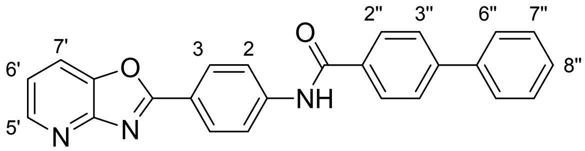

Prepared from **S43** (300 mg, 1.42 mmol) using general method B. **9** recovered after recrystallization (DCM) as an off-white solid (202 mg, 36%). **mp** 322-325 °C; **IR** (KBr) ν $_{max}$: 3358 (NH), 3067, 3056, 1672 (C=O), 1599; **^{1}H NMR** (400 MHz, DMSO-d$_6$) δ 10.70 (br s, 1H, N*H*), 8.54 (dd, *J* = 4.9, 1.4 Hz, 1H, C5'-H), 8.27 (d, *J* = 8.8 Hz, 2H, 2 × C3-H), 8.23 (dd, *J* = 8.1, 1.4 Hz, 1H, C7'-H), 8.17 – 8.06 (m, 4H, 2 × C2-H, 2 × C2''-H) 7.91 – 7.84 (m, 2H, 2 × C3''-H), 7.83 – 7.72 (m, 2H, 2 × C6''-H), 7.57 – 7.49 (m, 2H, 2 × C7''-H), 7.48 – 7.38 (m, 2H, C6'-H, C8''-H); **^{13}C NMR** (101 MHz, DMSO-d$_6$) δ 165.7 (CO), 164.8 (C2'), 155.8 (C4b), 146.4 (C5'), 143.5 (C), 143.4 (C), 142.7 (C5b), 139.0 (C5''), 133.3 (C1''), 129.1 (2 × C7''), 128.6 (2 × CH), 128.6 (2 × CH), 128.3 (C8''), 127.0 (2 × C6''), 126.7 (2 × C3''), 120.6 (C4), 120.5 (C6'), 120.3 (2 × C2), 118.8 (C7'); ***m/z*** (ES$^-$) 389.91 ([M-H]$^-$, 100 %); **HRMS** (ES$^-$) Calcd for $C_{25}H_{16}N_3O_2$ [M-H]$^-$: 390.1234, found 390.1243.

***N*-(2-fluoro-5-(oxazolo[4,5-b]pyridin-2-yl)phenyl)-[1,1'-biphenyl]-4-carboxamide (10)**

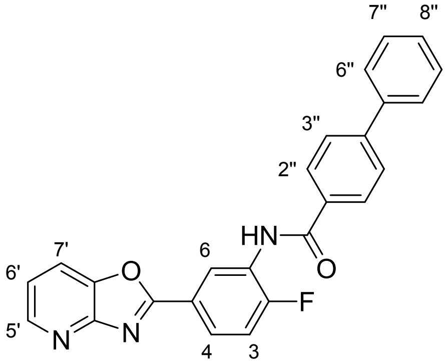

Prepared from **S44** (51 mg, 0.22 mmol) using general method B. **10** recovered after recrystallization (DCM) as a white solid (75 mg, 83%). **mp** 230-232 °C; **IR** (solid) ν $_{max}$: 3441 (NH), 3040, 1674 (C=O), 1520, 1404, 1250 (C-F); **^{1}H NMR** (400 MHz, DMSO-*d*$_6$) δ 10.47 (br s, 1H, N*H*), 8.65 (dd, *J* = 7.3, 2.3 Hz, 1H, C6-H), 8.57 (dd, *J* = 4.9, 1.4 Hz, 1H, C5'-H), 8.28 (dd, *J* = 8.2, 1.4 Hz, 1H, C7'-H), 8.20 – 8.15 (m, 1H, C4-H), 8.13 (d, *J* = 8.4 Hz, 2H, 2 × C2''-H), 7.94 – 7.83 (m, 2H, 2 × C3''-H), 7.83 – 7.74 (m, 2H, 2 × C6''-H), 7.62 (dd, *J* = 10.2, 8.7 Hz, 1H, C3-H), 7.58 – 7.37 (m, 4H, C6'-H, 2 × C7''-H, C8''-H); **^{13}C NMR** (101 MHz, DMSO-*d*$_6$) δ 165.3 (CO), 163.9 (C2'), 157.8 (d, *J* = 254.7 Hz, C2), 155.5 (C4b), 146.67 (C5'), 143.6 (C4''), 142.9 (C5b), 139.0 (C5''), 132.4 (C1''), 129.1 (2 × C7''), 128.7 (2 × C2''), 128.3 (C8''), 127.1 (C1), 127.0 (2 × C6''), 126.7 (2 × C3''), 126.3 (d, *J* = 9.1 Hz, C4), 125.7 (C6), 122.4 (d, *J* = 3.0 Hz, C5), 120.9 (C6'), 119.2 (C7'),

117.4 (d, J = 21.6 Hz, C3); ***m/z*** (ES^+) 410.12 ($[M+H]^+$, 100 %); **HRMS** (ES^+) Calcd for $C_{25}H_{17}FN_3O_2$ $[M+H]^+$: 410.1299, found 410.1299.

***N*-(2-bromo-5-(oxazolo[4,5-b]pyridin-2-yl)phenyl)-[1,1'-biphenyl]-4-carboxamide (11 or Liddean)**

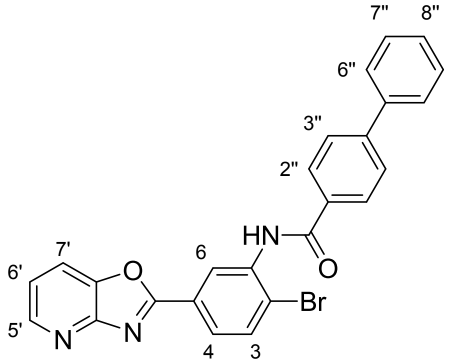

Prepared from **S45** (300 mg, 1.04 mmol) using general method B. **Liddean** recovered after recrystallization (DCM) as a white solid (200 mg, 41%). **mp** 237-240 °C; **IR** (KBr) ν $_{max}$: 3460 (NH), 3271, 2666, 1653 (C=O), 1521; **^{1}H NMR** (500 MHz, DMSO-d_6) δ 10.34 (br s, 1H, N*H*), 8.58 (dd, J = 4.8, 1.4 Hz, 1H, C5'-H), 8.49 (d, J = 2.0 Hz, 1H, C6-H), 8.29 (dd, J = 8.1, 1.4 Hz, 1H, C7'-H), 8.18 – 8.11 (m, 2H, 2 × C2''-H), 8.07 (dd, J = 8.4, 2.1 Hz, 1H, C4-H), 8.03 (d, J = 8.4 Hz, 1H, C3-H), 7.93 – 7.86 (m, 2H, 2 × C3''-H), 7.79 (m, 2H, 2 × C6''-H), 7.56 – 7.47 (m, 3H, C6'-H, 2 × C7''-H), 7.47 – 7.40 (m, 1H, C8''-H); **^{13}C NMR** (126 MHz, DMSO-d_6) δ 165.2 (CO), 163.8 (C2'), 155.4 (C4b), 146.9 (C5'), 143.7 (C4''), 143.0 (C5b), 139.1 (C5''), 137.6 (C1), 134.2 (C3), 132.5 (C1''), 129.1 (2 × C7''), 128.5 (2 × C2''), 128.3 (C8''), 127.0 (2 × C6''), 126.9 (C6), 126.9 (2 × C3''), 126.4 (C4), 125.8 (C5), 124.9 (C2), 121.2 (C6'), 119.4 (C7'); ***m/z*** (ES^-) 467.81 ($[^{79}BrM-H]^+$, 100 %), 469.81 ($[^{81}BrM-H]^-$, 100%); **HRMS** (ES^-) Calcd for $C_{25}H_{15}O_2N_3Br$ $[M-H]^-$: 468.0348, found 468.0351.

***N*-(3-(oxazolo[4,5-b]pyridin-2-yl)phenyl)-[1,1'-biphenyl]-4-sulfonamide (12)**

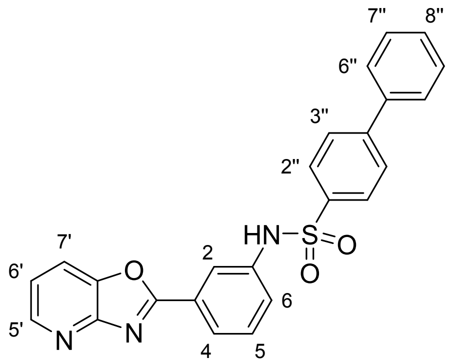

Prepared from **S42** (85 mg, 0.4 mmol) using the general method B. **12** recovered after recrystallization (DCM) as a white solid (120 mg, 70%). **mp** 193-195 °C; **IR** (KBr) ν $_{max}$: 3067 (NH), 2893, 1551, 1404, 1337

(S=O), 1157 (S=O), 945, 777, 762; **^{1}H NMR** (500 MHz, DMSO-d_6) δ 10.82 (br s, 1H, N*H*), 8.55 (dd, *J* = 4.8, 1.4 Hz, 1H, C5'-H), 8.26 (dd, *J* = 8.2, 1.4 Hz, 1H, C7'-H), 8.07 (t, *J* = 1.9 Hz, 1H, C2-H), 7.94 – 7.83 (m, 5H, C4-H, 2 × C2''-H, 2 × C3''-H), 7.71 – 7.64 (m, 2H, 2 × C6''-H), 7.54 (t, *J* = 8.0 Hz, 1H, C5-H), 7.49 – 7.43 (m, 4H, C6'-H, C6-H, 2 × C7''-H), 7.43 – 7.36 (m, 1H, C8''-H); **^{13}C NMR** (126 MHz, DMSO-d_6) δ 164.2 (C2'), 155.3 (C4b), 146.7 (C5'), 144.6 (C4''), 142.8 (C5b), 138.8 (C1), 138.1 (C), 138.0 (C), 130.6 (C5), 129.1 (2 × C7''), 128.7 (C8''), 127.6 (2 × CH), 127.4 (2 × CH), 127.1 (2 × C6''), 126.9 (C3), 123.5 (C6), 123.2 (C4), 121.0 (C6'), 119.3 (C7'), 118.2 (C2); ***m/z*** (ES$^+$) 428.10 ([M+H]$^+$, 100 %); **HRMS** (ES$^+$) Calcd for $C_{24}H_{18}O_3N_3S$ [M+H]$^+$: 428.1063, found 428.1063.

3-(Oxazolo[4,5-b]pyridin-2-yl)phenyl [1,1'-biphenyl]-4-carboxylate (13)

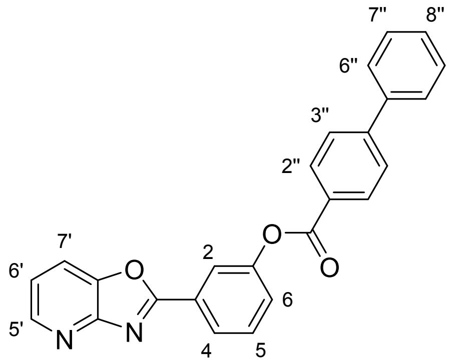

Prepared from **S46** (85 mg, 0.4 mmol) using the general method B. **13** recovered after recrystallization (DCM) as a white solid (95 mg, 61%). **mp** 185-187 °C; **IR** (solid) ν_{max}: 3063, 1728 (C=O), 1605, 1551, 1481, 1404; **^{1}H NMR** (500 MHz, Chloroform-*d*) δ 8.61 (dd, *J* = 4.8, 1.5 Hz, 1H, C5'-H), 8.30 (dd, *J* = 8.4, 1.8 Hz, 2H, 2 × C2''-H), 8.27 (dt, *J* = 7.9, 1.3 Hz, 1H, C4-H), 8.22 (t, *J* = 1.9 Hz, 1H, C2-H), 7.88 (dd, *J* = 8.2, 1.5 Hz, 1H, C7'-H), 7.77 (dd, *J* = 8.4, 1.8 Hz, 2H, 2 × C3''-H), 7.68 (dt, *J* = 6.0, 1.4 Hz, 2H, 2 × C6''-H), 7.64 (t, *J* = 7.9 Hz, 1H, C5-H), 7.54 – 7.47 (m, 3H, C6-H, 2 × C7''-H), 7.47 – 7.40 (m, 1H, C8''-H), 7.32 (dd, *J* = 8.2, 4.8 Hz, 1H, C6'-H); **^{13}C NMR** (126 MHz, Chloroform-*d*) δ 164.9 (C), 164.8 (C), 156.3 (C4b), 151.5 (C1), 147.1 (C5'), 146.8 (C4''), 143.3 (C5b), 139.9 (C5''), 130.9 (2 × C2''), 130.4 (C5), 129.2 (2 × C7''), 128.5 (C8''), 128.1

(C3), 127.9 (C1''), 127.5 (2 × C6'', 2 × C3''), 126.1 (C6), 125.8 (C4), 121.6 (C2), 120.5 (C6'), 118.5 (C7'); ***m/z*** (ES$^+$) 393.12 ([M+H]$^+$, 100 %); **HRMS** (ES$^+$) Calcd for $C_{25}H_{17}O_3N_2$ [M+H]$^+$: 393.1234, found 393.1233.

***N*-(5-(benzo[d]oxazol-2-yl)-2-bromophenyl)-[1,1'-biphenyl]-4-carboxamide (14)**

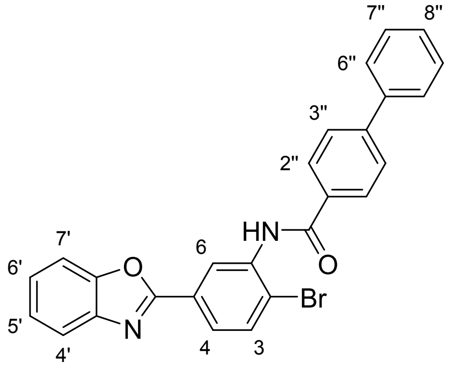

Prepared from **S49** (289 mg, 1.00 mmol) using general method B. **14** recovered after recrystallization (DCM) as a white solid (250 mg, 53%). **mp** 260-262 °C; **IR** (KBr) ν $_{max}$: 3269 (NH), 1653 (C=0), 1558, 1508, 1240; **^{1}H NMR** (500 MHz, Chloroform-*d*) δ 9.48 (d, *J* = 2.1 Hz, 1H, C6-H), 8.61 (br s, 1H, N*H*), 8.10 – 8.03 (m, 2H, 2 × C2''-H), 7.96 (dd, *J* = 8.4, 2.1 Hz, 1H, C4-H), 7.82 – 7.73 (m, 4H, C3-H, C4'-H, 2 × C3''-H), 7.70 – 7.64 (m, 2H, 2 × C6''-H), 7.64 – 7.60 (m, 1H, C7'-H), 7.52 – 7.46 (m, 2H, 2 × C7''-H), 7.45 – 7.40 (m, 1H, C8''-H), 7.39 – 7.36 (m, 2H, C5'-H, C6'-H); **^{13}C NMR** (126 MHz, Chloroform-*d*) δ 165.1 (CO), 162.1 (C2'), 151.0 (C5b), 145.5 (C4''), 142.2 (C4b), 139.9 (C5''), 136.6 (C1), 133.1 (C3), 132.9 (C1''), 129.2 (2 × C7''), 128.4 (C8''), 127.9 (2 × C2'', 2 × C3''), 127.4 (2 × C6''), 125.6 (ArCH), 124.9 (ArCH), 124.9 (C5), 124.3 (C4), 120.5 (C6), 120.3 (C4'), 117.0 (C2), 111.0 (C7'); ***m/z*** (ES$^+$) 469.05 ([^{79}BrM+H]$^+$, 100 %), 471.05 ([^{81}BrM+H]$^+$, 100%); **HRMS** (ES$^+$) Calcd for $C_{26}H_{18}O_2N_2Br$ [M+H]$^+$: 469.0546, found 469.0544.

***N*-(2-chlorophenyl)-[1,1'-biphenyl]-4-carboxamide (15)**

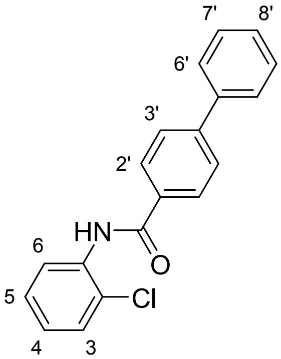

Prepared from 2-chloroaniline (128 mg, 1.00 mmol) using general method B. **15** recovered after recrystallization (DCM) as a white solid (250 mg, 81%). **mp** 144-146 °C; **IR** (solid) ν $_{max}$: 3279 (NH), 1643 (C=O), 1520, 1435, 1312, 741; **^{1}H NMR** (500 MHz, DMSO-d_6) δ 10.13 (br s, 1H, N*H*), 8.10 (d, *J* = 8.4 Hz, 2H, 2 × C2’-H), 7.85 (d, *J* = 8.4 Hz, 2H, 2 × C3’-H), 7.80 – 7.73 (m, 2H, 2 × C6’-H), 7.62 (dd, *J* = 7.7, 1.6 Hz, 1H, C6-H), 7.57 (dd, *J* = 8.1, 1.6 Hz, 1H, C3-H), 7.55 – 7.49 (m, 2H, 2 × C7’-H), 7.47 – 7.40 (m, 1H, C8’-H), 7.40 (dd, *J* = 7.7, 1.6 Hz, 1H, C5-H), 7.31 (td, *J* = 7.7, 1.6 Hz, 1H, C4-H); **^{13}C NMR** (126 MHz, DMSO-d_6) δ 165.0 (CO), 143.4 (C4’), 139.1 (C5’), 135.1 (C1), 132.7 (C1’), 129.6 (C3), 129.1 (C2, 2 × C7’), 128.6 (C6), 128.4 (2 × C2’), 128.2 (C8’), 127.5 (C4, C5), 127.0 (2 × C6’), 126.7 (2 × C3’); ***m/z*** (ES$^+$) 308.08 ([M+H]$^+$, 100 %); **HRMS** (ES$^+$) Calcd for $C_{19}H_{15}ClNO$ [M+H]$^+$: 308.0837, found 308.0839.

***N*-(2-bromo-5-(oxazolo[4,5-b]pyridin-2-yl)phenyl)benzamide (S31)**

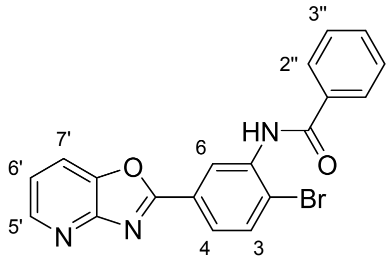

Prepared from **S45** (80 mg, 0.28 mmol) using general method B. **S31** recovered after recrystallization (DCM) as a white solid (74 mg, 67%). **mp** 249-251 °C; **IR** (solid) ν $_{max}$: 3271 (NH), 3055, 1651(C=O), 1520, 1404, 1257, 779, 709, 640 (C-Br); **^{1}H NMR** (400 MHz, DMSO-d_6) δ 10.29 (br s, 1H, N*H*), 8.58 (dd, *J* = 4.9, 1.4 Hz, 1H, C5’-H), 8.47 (d, *J* = 2.0 Hz, 1H, C6-H), 8.28 (dd, *J* = 8.3, 1.4 Hz, 1H, C7’-H), 8.10 – 7.98 (m, 4H, C3-H, C4-H, 2 × C2’’-H), 7.69 – 7.62 (m, 1H, C4’’-H), 7.58 (dd, *J* = 8.3, 6.5 Hz, 2H, 2 × C3’’-H), 7.50 (dd, *J* = 8.3, 4.9 Hz, 1H, C6’-H); **^{13}C NMR** (101 MHz, DMSO-d_6) δ 165.6 (CO), 163.7 (C2’), 155.3 (C4b), 146.8 (C5’), 142.9 (C5b), 137.6 (C1), 134.1 (C3), 133.8 (C1’’), 132.1 (C4’’), 128.6 (2 × C2’’), 127.8 (2 × C3’’), 126.8 (C6), 126.4 (C4), 125.8 (C5), 124.9 (C2), 121.1 (C6’), 119.3 (C7’); ***m/z*** (ES$^+$) 394.02 ([^{79}BrM+H]$^+$, 100 %), 396.02 ([^{81}BrM+H]$^+$, 100%); **HRMS** (ES$^+$) Calcd for $C_{19}H_{13}O_2N_3Br$ [M+H]$^+$: 394.0186, found 394.0188.

***N*-(2-bromo-5-(oxazolo[4,5-b]pyridin-2-yl)phenyl)-2-(3-methoxyphenyl)acetamide (S32)**

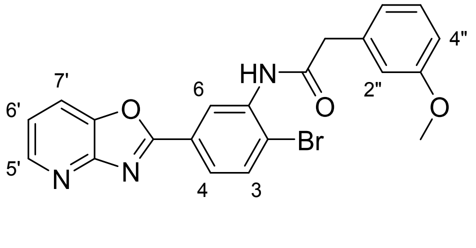

Prepared from **S45** (80 mg, 0.28 mmol) using general method B. **S32** recovered after recrystallization (DCM) as a white solid (92 mg, 75%). **mp** 211-213 °C; **IR** (solid) ν $_{max}$: 3271 (NH), 1667 (C=O), 1551, 1404, 1265, 1157, 802; **^{1}H NMR** (400 MHz, DMSO-d_6) δ 9.87 (br s, 1H, N*H*), 8.56 (dd, *J* = 4.8, 1.4 Hz, 1H, C5'-H), 8.52 (s, 1H, C6-H), 8.27 (dd, *J* = 8.3, 1.4 Hz, 1H, C7'-H), 7.94 (s, 2H, C3-H, C4-H), 7.48 (dd, *J* = 8.3, 4.8 Hz, 1H, C6'-H), 7.27 (t, *J* = 7.8 Hz, 1H, C5''-H), 7.02 – 6.95 (m, 2H, C2''-H, C6''-H), 6.85 (dd, *J* = 7.8, 2.3 Hz, 1H, C4''-H), 3.78 (s, 2H, CH_2), 3.76 (s, 3H, OCH_3); **^{13}C NMR** (101 MHz, DMSO-d_6) δ 169.7 (CO), 163.8 (C2'), 159.3 (C3''), 155.3 (C4b), 146.8 (C5'), 142.9 (C5b), 137.2 (C), 137.0 (C), 134.1 (C3), 129.4 (C5''), 125.6 (C5), 125.4 (C4), 124.7 (C6), 121.6 (C2), 121.5 (C6''), 121.1 (C6'), 119.3 (C7'), 115.0 (C2''), 112.2 (C4''), 55.0 (OCH_3), 42.6 (CH_2); ***m/z*** (ES$^+$) 438.04 ([^{79}BrM+H]$^+$, 100 %), 440.04 ([^{81}BrM+H]$^+$, 100%); **HRMS** (ES$^+$) Calcd for $C_{21}H_{17}O_3N_3Br$ [M+H]$^+$: 438.0448, found 438.0449.

***N*-(2-methyl-5-(oxazolo[4,5-b]pyridin-2-yl)phenyl)-[1,1'-biphenyl]-4-carboxamide (S33)**

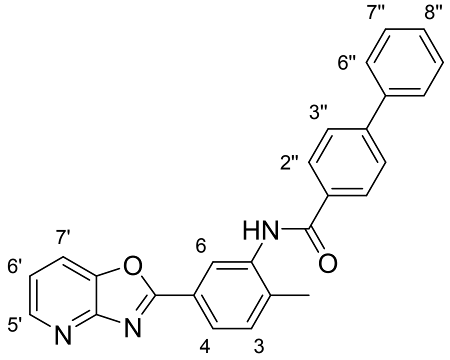

Prepared from **S47** (600 mg, 2.66 mmol) using general method B. **S33** recovered after recrystallization (DCM) as a white solid (360 mg, 33%). **mp** 275-277 °C; **IR** (solid) ν $_{max}$: 3264 (NH), 1670 (C=O), 1520, 1404, 1258, 741; **^{1}H NMR** (400 MHz, DMSO-d_6) δ 10.17 (br s, 1H, N*H*), 8.55 (dd, *J* = 4.8, 1.4 Hz, 1H, C5'-H), 8.36 (d, *J* = 1.9 Hz, 1H, C6-H), 8.26 (dd, *J* = 8.2, 1.4 Hz, 1H, C7'-H), 8.14 (d, *J* = 8.3 Hz, 2H, 2 × C2''-H), 8.06 (dd, *J* = 8.0, 1.9 Hz, 1H, C4-H), 7.88 (d, *J* = 8.3 Hz, 2H, 2 × C3''-H), 7.81 – 7.73 (m, 2H, 2 × C6''-H), 7.58 (d, *J* = 8.0 Hz, 1H, C3-H), 7.53 (t, *J* = 7.5 Hz, 2H, 2 × C7''-H), 7.49 – 7.46 (m, 1H, C6'-H), 7.48 – 7.39 (m, 1H, C8''-H), 2.41 (s, 3H, CH_3); **^{13}C NMR** (101 MHz, DMSO-d_6) δ 165.3 (CO), 164.7 (C2'), 155.6 (C4b), 146.6 (C5'), 143.3 (C4''), 142.8 (C5b), 139.1 (C5''), 138.8 (C2), 137.4 (C1), 133.0 (C1''), 131.6 (C3), 129.1

(2 × C7''), 128.5 (2 × C2''), 128.2 (C8''), 127.0 (2 × C6''), 126.7 (2 × C3''), 125.2 (C6), 124.9 (C4), 123.9 (C5), 120.8 (C6'), 119.0 (C7'), 18.3 (C̲H₃); ***m/z*** (ES$^+$) 406.15 ([M+H]$^+$, 100 %); **HRMS** (ES$^+$) Calcd for $C_{26}H_{20}N_3O_2$ [M+H]$^+$: 406.1550, found 406.1548.

***N*-(4-hydroxy-3-(oxazolo[4,5-b]pyridin-2-yl)phenyl)-[1,1'-biphenyl]-4-carboxamide (S34)**

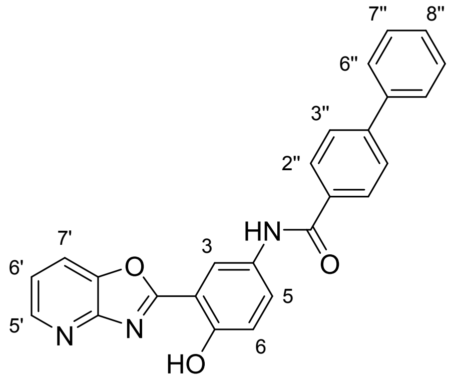

Prepared from **S48** (300 mg, 1.32 mmol) the general method B. **S34** recovered after recrystallization (DCM) as an off-white solid (346 mg, 64%). **mp** 330-332 °C; **IR** (KBr) ν $_{max}$: 3425 (NH), 3257, 3094, 3044, 1636 (C=O), 1534; **^{1}H NMR** (500 MHz, DMSO-d_6) δ 10.85 (br s, 1H, O*H*), 10.44 (br s, 1H, N*H*), 8.72 (d, *J* = 2.7 Hz, 1H, C3-H̲), 8.59 (dd, *J* = 4.7, 1.4 Hz, 1H, C5'-H̲), 8.35 (dd, *J* = 7.9, 1.4 Hz, 1H, C7'-H̲), 8.11 (d, *J* = 7.9 Hz, 2H, 2 × C2''-H̲), 7.92 (dd, *J* = 8.9, 2.7 Hz, 1H, C5-H̲), 7.86 (d, *J* = 8.2 Hz, 2H, 2 × C3''-H̲), 7.77 (d, *J* = 7.3 Hz, 2H, 2 × C6''-H̲), 7.58 – 7.48 (m, 3H, C6'-H̲, 2 × C7''-H̲), 7.43 (t, *J* = 7.3 Hz, 1H, C8''-H̲), 7.18 (d, *J* = 8.9 Hz, 1H, C6-H̲); **^{13}C NMR** (126 MHz, DMSO-d_6) δ 165.0 (CO), 164.6 (C2'), 154.4 (C1), 153.9 (C4b), 146.8 (C5'), 143.2 (C4''), 141.7 (C5b), 139.1 (C5''), 133.4 (C1''), 131.8 (C4), 129.1 (2 × C7''), 128.3 (2 × C2''), 128.2 (C8''), 127.5 (C5), 127.0 (2 × C6''), 126.7 (2 × C3''), 121.1 (C6'), 119.3 (C7'), 119.1 (C3), 117.6 (C6), 109.8 (C2); ***m/z*** (ES$^-$) 405.88 ([M-H]$^-$, 100 %); **HRMS** (ES$^-$) Calcd for $C_{25}H_{16}N_3O_3$ [M-H]$^-$: 406.1185, found 406.1192.

***N*-(2-bromo-5-(oxazolo[4,5-b]pyridin-2-yl)phenyl)-[1,1'-biphenyl]-4-sulfonamide (S35)**

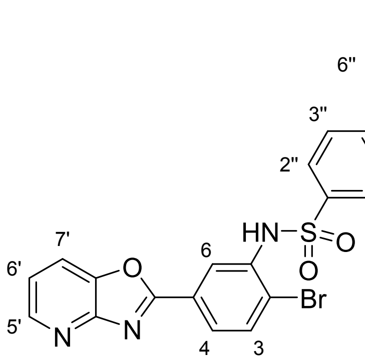

Prepared from **S45** (80 mg, 0.28 mmol) using the general method B. **S35** recovered after recrystallization (DCM) as a white solid (75 mg, 53%). **mp** 240-242 °C; **IR** (solid) ν $_{max}$: 3233 (NH), 1605, 1543, 1474, 1396 (S=O), 1342, 1258, 1157 (S=O); ^{1}**H NMR** (500 MHz, Chloroform-*d*) δ 8.66 – 8.60 (m, 2H, C5'-H, C6-H), 7.98 (dd, *J* = 8.4, 2.0 Hz, 1H, C4-H), 7.96 – 7.88 (m, 3H, C7'-H, 2 × C2''-H), 7.66 (d, *J* = 8.4 Hz, 2H, 2 × C3''-H), 7.63 (d, *J* = 8.4 Hz, 1H, C3-H), 7.60 – 7.54 (m, 2H, 2 × C6''-H), 7.44 (t, *J* = 7.6 Hz, 2H, 2 × C7''-H), 7.40 (d, *J* = 6.8 Hz, 1H, C8''-H), 7.36 (dd, *J* = 8.1, 4.9 Hz, 1H, C6'-H), 7.17 (br s, 1H, N*H*); ^{13}**C NMR** (126 MHz, Chloroform-*d*) δ 164.1 (C2'), 156.2 (C4b), 147.3 (C5'), 146.6 (C4''), 143.4 (C5b), 139.0 (C5''), 137.1 (C1''), 135.7 (C1), 133.7 (C3), 129.2 (2 × C7''), 128.8 (C8''), 128.1 (2 × C2''), 127.9 (2 × C3''), 127.4 (2 × C6''), 127.3 (C5), 125.8 (C4), 121.0 (C6), 120.8 (C6'), 120.0 (C2), 118.7 (C7'); ***m/z*** (ES^{+}) 506.02 ([^{79}BrM+H]$^{+}$, 100 %), 508.02 ([^{81}BrM+H]$^{+}$, 100%); **HRMS** (ES^{+}) Calcd for $C_{24}H_{17}O_3N_3BrS$ [M+H]$^{+}$: 506.0169, found 506.0160.

***N*-(2-bromo-5-nitrophenyl)-[1,1'-biphenyl]-4-carboxamide (S38)**

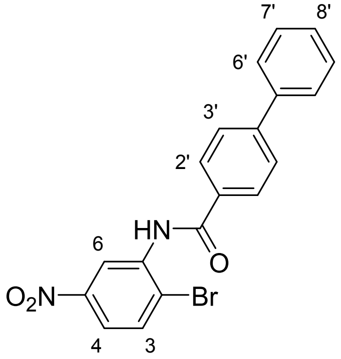

Biphenyl-4-carbonyl chloride (461 mg, 2.13 mmol, 1.0 eq.) was added to 2-bromo-5-nitroaniline (308 mg, 1.42 mmol, 1.5 eq.) in DCM (15 mL), followed by pyridine (0.17 mL, 2.13 mmol, 1.5 eq.). The

reaction was stirred at room temperature for 18 h. **S38** was recovered after recrystallization (DCM) as a white solid (300 mg, 53%). **mp** 203-205 °C; **IR** (solid) ν $_{max}$: 3379 (NH), 1690 (C=O), 1528 (N-O), 1412, 1312, 741, 602 (C-Br); **^{1}H NMR** (500 MHz, DMSO-d_6) δ 10.38 (s, 1H, N*H*), 8.51 (d, *J* = 1.8 Hz, 1H, C6-H), 8.12 (d, *J* = 8.1 Hz, 2H, 2 × C2'-H), 8.06 (s, 2H, C3-H, C4-H), 7.89 (d, *J* = 8.1 Hz, 2H, 2 × C3'-H), 7.81 – 7.75 (m, 2H, 2 × C6'-H), 7.52 (t, *J* = 7.5 Hz, 2H, 2 × C7'-H), 7.44 (t, *J* = 7.5 Hz, 1H, C8'-H); **^{13}C NMR** (126 MHz, DMSO-d_6) δ 165.2 (CO), 146.9 (C5), 143.8 (C4'), 139.0 (C5'), 137.8 (C1), 134.0 (C3), 132.2 (C1'), 129.1 (2 × C7'), 128.6 (2 × C2'), 128.3 (C8'), 127.5 (C2), 127.0 (2 × C6'), 126.8 (2 × C3'), 122.3 (C6), 121.9 (C4); ***m/z*** (ES$^+$) 497.02 ([^{79}BrM+Na]$^+$, 100 %), 399.02 ([^{81}BrM+H]$^+$, 100%); **HRMS** (ES$^+$) Calcd for $C_{19}H_{14}BrN_2O_3$ [M+H]$^+$: 397.0182, found 397.0184.

***N*-(5-amino-2-bromophenyl)-[1,1'-biphenyl]-4-carboxamide (S39)**

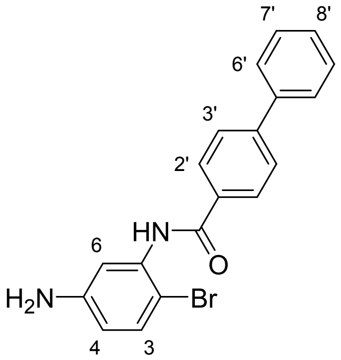

To **S38** (100 mg, 0.25 mmol, 1.0 eq.) and NH_4Cl (67 mg, 1.25 mmol, 5.0 eq.) in MeOH (2 mL) and H_2O (1 mL) at 100 °C was added iron powder (70 mg, 1.25 mmol, 5.0 eq.) portionwise. After 3 h the reaction was cooled to room temperature, filtered through Celite and concentrated *in vacuo*. The residue was dissolved in EtOAc (10 mL) and washed with H_2O (2 × 5 mL), brine (5 mL), dried over $MgSO_4$, filtered and concentrated to yield the desired product **S39** as a white solid (83 mg, 90%). **S39** was used in the next step without further purification. **^{1}H NMR** (500 MHz, DMSO-d_6) δ 9.77 (br s, 1H, N*H*), 8.11 – 7.99 (m, 2H, 2 × C2'-H), 7.89 – 7.79 (m, 2H, 2 × C3'-H), 7.79 – 7.73 (m, 2H, 2 × C6'-H), 7.55 – 7.46 (m, 2H, 2 × C7'-H), 7.46 – 7.39 (m, 1H, C8'-H), 7.27 (d, *J* = 8.6 Hz, 1H, C3-H), 6.85 (d, *J* = 2.7 Hz, 1H, C6-H), 6.44 (dd, *J* = 8.6, 2.7 Hz, 1H, C4-H), 5.39 (br s, 2H, N*H*$_2$); **^{13}C NMR** (126 MHz, DMSO-d_6) δ 164.7 (CO), 148.8 (C5), 143.2 (C4'), 139.1 (C5'), 136.4 (C1), 133.1 (C1'), 132.3 (C3), 129.1 (2 × C7'), 128.3 (2 × C2'), 128.2 (C8'), 127.0 (2 × C6'), 126.7 (2 × C3'), 113.6 (C4, C6), 104.5 (C2);

***N*-(3-([1,1'-biphenyl]-4-carboxamido)-4-bromophenyl)furan-2-carboxamide (S36)**

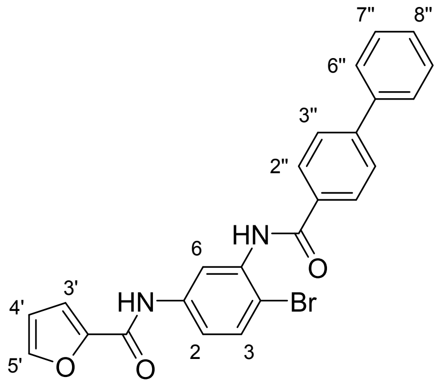

2-Furoyl chloride (47 mg, 0.36 mmol, 3.0 eq.) was added to **S39** (44 mg, 0.12 mmol, 1.0 eq.) in DCM (1.2 mL), followed by pyridine (29 µL, 0.36 mmol, 3.0 eq.). The reaction was stirred at room temperature for 18 h. The reaction was concentrated *in vacuo* and purified *via* the Biotage SP4 (silica-packed SNAP column 4 g; 0-4% MeOH/DCM) to give the title product **S36** as a white solid (40 mg, 72%). **mp** 210-212 °C; **IR** (solid) ν_{max}: 3410 (NH), 3317 (NH), 1674 (C=O), 1647 (C=O), 1508, 1260, 750; **^{1}H NMR** (500 MHz, Chloroform-*d*) δ 8.58 (d, *J* = 2.6 Hz, 1H, C6-H), 8.57 (br s, 1H, N*H*), 8.24 (br s, 1H, N*H*), 8.04 – 8.00 (m, 2H, 2 × C2''-H), 7.94 (dd, *J* = 8.8, 2.6 Hz, 1H, C2-H), 7.79 – 7.74 (m, 2H, 2 × C3''-H), 7.69 – 7.63 (m, 2H, 2 × C6''-H), 7.58 (d, *J* = 8.8 Hz, 1H, C3-H), 7.54 (dd, *J* = 1.7, 0.9 Hz, 1H, C5'-H), 7.53 – 7.46 (m, 2H, 2 × C7''-H), 7.45 – 7.38 (m, 1H, C8''-H), 7.27 (d, *J* = 0.9 Hz, 1H, C3'-H), 6.58 (dd, *J* = 3.5, 1.7 Hz, 1H, C4'-H); **^{13}C NMR** (126 MHz, Chloroform-*d*) δ 165.2 (CO), 156.2 (CO), 147.6 (C2'), 145.4 (C4''), 144.6 (C5'), 139.9 (C5''), 137.9 (C5), 136.2 (C1), 133.1 (C1''), 132.9 (C3), 129.2 (2 × C7''), 128.4 (C8''), 127.8 (2 × C2'', 2 × C3''), 127.4 (2 × C6'), 117.0 (C2), 115.8 (C3'), 112.9 (C4'), 112.3 (C6), 108.0 (C4); ***m/z*** (ES$^+$) 461.05 ([^{79}BrM+Na]$^+$, 100 %), 463.05 ([^{81}BrM+H]$^+$, 100%); **HRMS** (ES$^+$) Calcd for $C_{24}H_{18}BrN_2O_3$ [M+H]$^+$: 461.0495, found 461.0493.

***N*-(3-([1,1'-biphenyl]-4-carboxamido)-4-bromophenyl)thiophene-2-carboxamide (S37)**

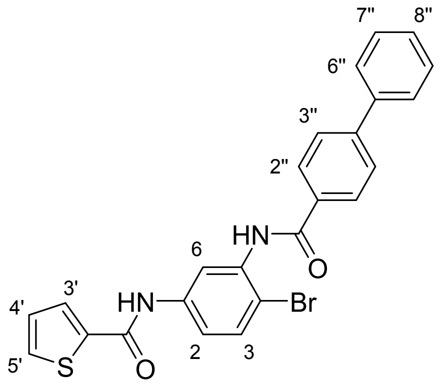

2-Thiophene carbonyl chloride (36 mg, 0.25 mmol, 3.0 eq.) was added to **S39** (30 mg, 0.08 mmol, 1.0 eq.) in DCM (1.0 mL), followed by pyridine (20 µL, 0.25 mmol, 3.0 eq.). The reaction was stirred at room temperature for 18 h. The reaction was concentrated *in vacuo* and purified *via* the Biotage SP4 (silica-packed SNAP column 4 g; 0-40% EtOAc/hexanes) to give the title product **S37** as a white solid (30 mg, 85%). **mp** 189-191 °C; **IR** (solid) ν_{max}: 3410 (NH), 3279 (NH), 1643 (C=O), 1589, 1515, 1420, 1258, 1018, 802, 741, 694; **^{1}H NMR** (500 MHz, Chloroform-*d*) δ 8.58 – 8.47 (m, 2H, C6-H, N*H*), 8.11 (br s, 1H, N*H*), 8.02 – 7.95 (m, 2H, 2 × C2''-H), 7.91 (dd, *J* = 8.8, 2.6 Hz, 1H, C2-H), 7.77 – 7.69 (m, 2H, 2 × C3''-H), 7.68 – 7.60 (m, 3H, C3'-H, 2 × C6''-H), 7.56 (d, *J* = 8.8 Hz, 1H, C3-H), 7.54 (dd, *J* = 5.0, 1.2 Hz, 1H, C5'-H), 7.52 – 7.46 (m, 2H, 2 × C7''-H), 7.45 – 7.39 (m, 1H, C8''-H), 7.11 (dd, *J* = 5.0, 3.7 Hz, 1H, C4'-H); **^{13}C NMR** (126 MHz, Chloroform-*d*) δ 165.4 (CO), 160.2 (CO), 145.4 (C4''), 139.8 (C5''), 139.1 (C2'), 138.3 (C5), 136.0 (C1), 133.0 (C1''), 132.8 (C3), 131.3 (C5'), 129.2 (2 × C7''), 128.8 (C3'), 128.4 (C8''), 128.1 (C4'), 127.8 (2 × C2'', 2 × C3''), 127.4 (2 × C6'), 117.5 (C2), 112.7 (C6), 108.2 (C4); ***m/z*** (ES$^+$) 499.01 ([^{79}BrM+Na]$^+$, 100 %), 501.01 ([^{81}BrM+H]$^+$, 100%); **HRMS** (ES$^+$) Calcd for $C_{24}H_{17}BrN_2O_2SNa$ [M+Na]$^+$: 499.0086, found 499.0080

5 ^{1}H NMR Spectra of Purchased Compounds (Chembridge)

2-(1-Adamantyl)-*N*-(2-chloro-5-[1,3]oxazolo[4,5-b]pyridin-2-ylphenyl)acetamide (3)

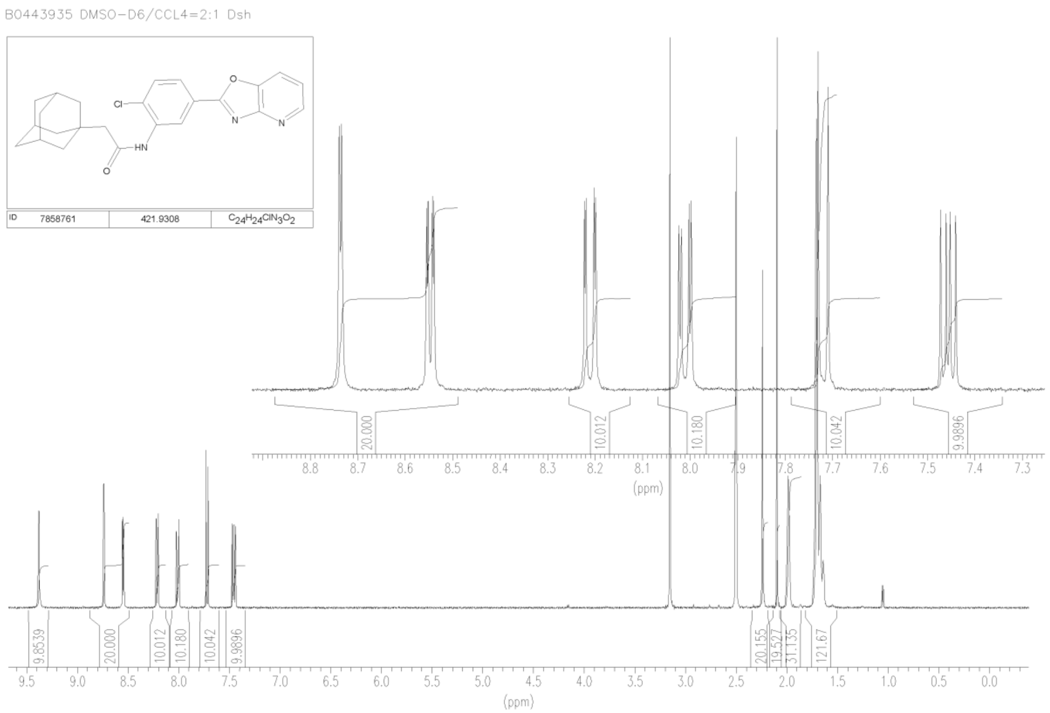

N-(2-chloro-5-[1,3]oxazolo[4,5-b]pyridin-2-ylphenyl)-1-naphthamide (4)

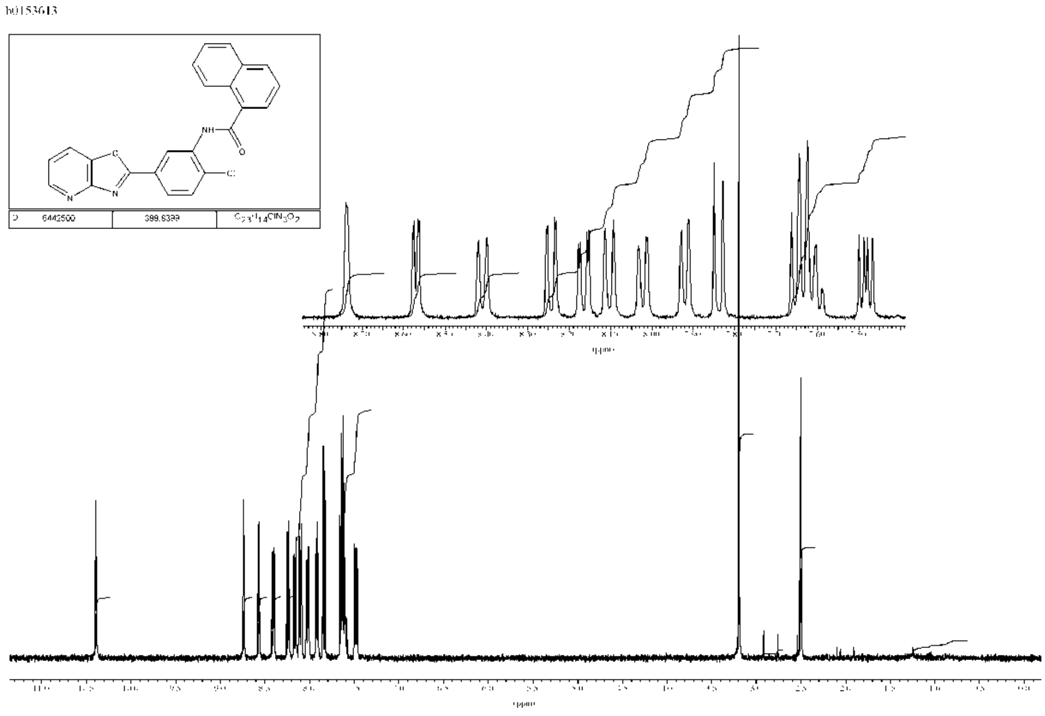

3-(Benzyloxy)-*N*-(2-chloro-5-[1,3]oxazolo[4,5-b]pyridin-2-ylphenyl)benzamide (5)

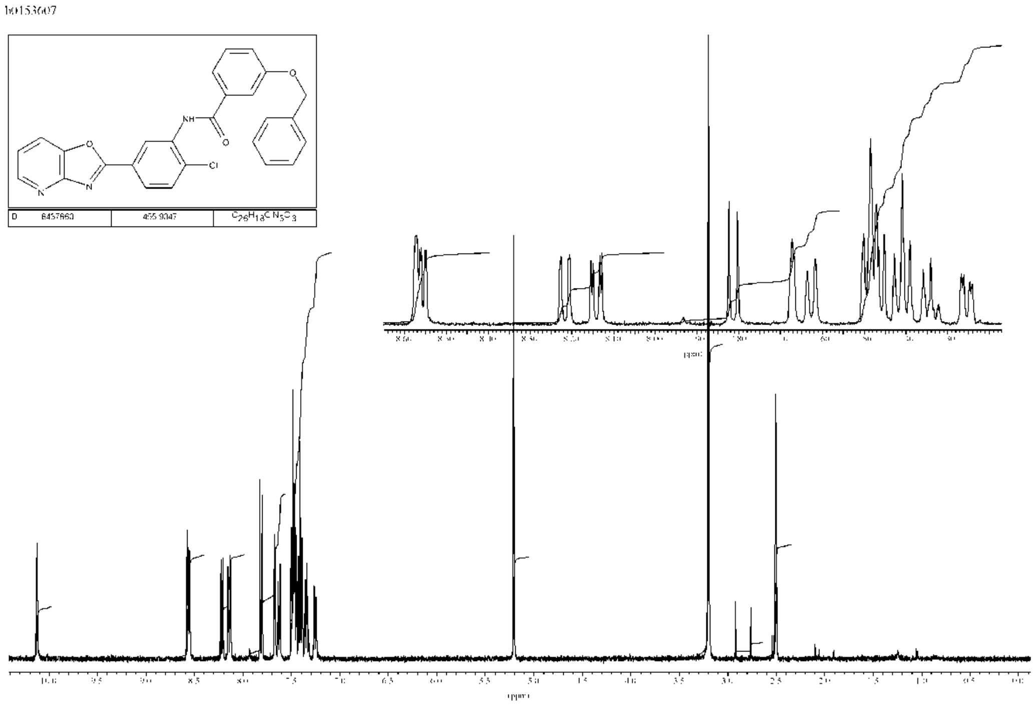

_N_-(2-chloro-5-[1,3]oxazolo[4,5-b]pyridin-2-ylphenyl)acetamide (6)

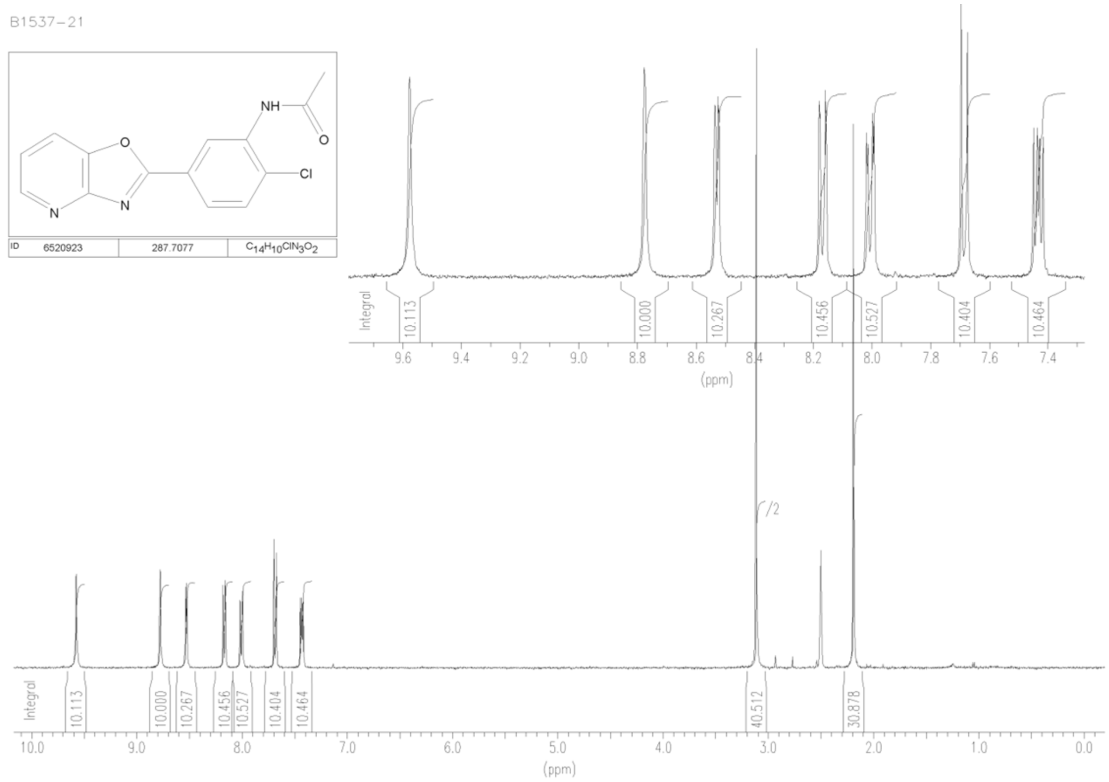

_N_-(2-chloro-5-[1,3]oxazolo[4,5-b]pyridin-2-ylphenyl)-2-(4-cyclohexylphenoxy)acetamide (S29)

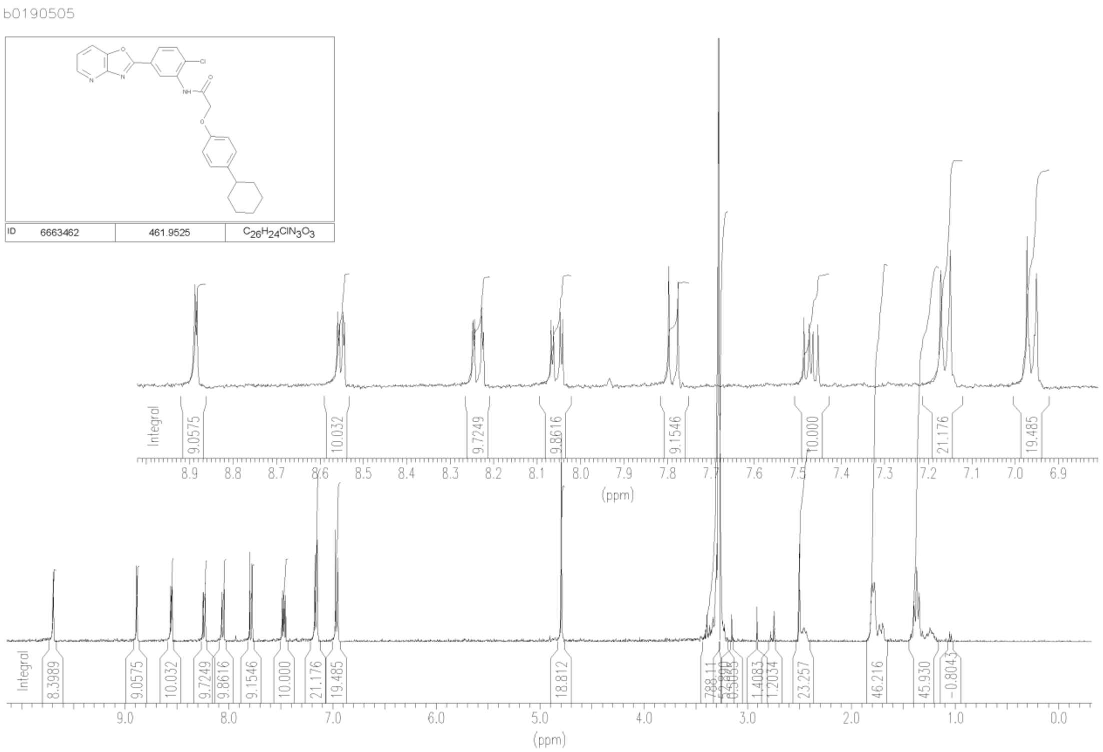

5-Bromo-*N*-(2-chloro-5-[1,3]oxazolo[4,5-b]pyridin-2-ylphenyl)-2-furamide (S30)

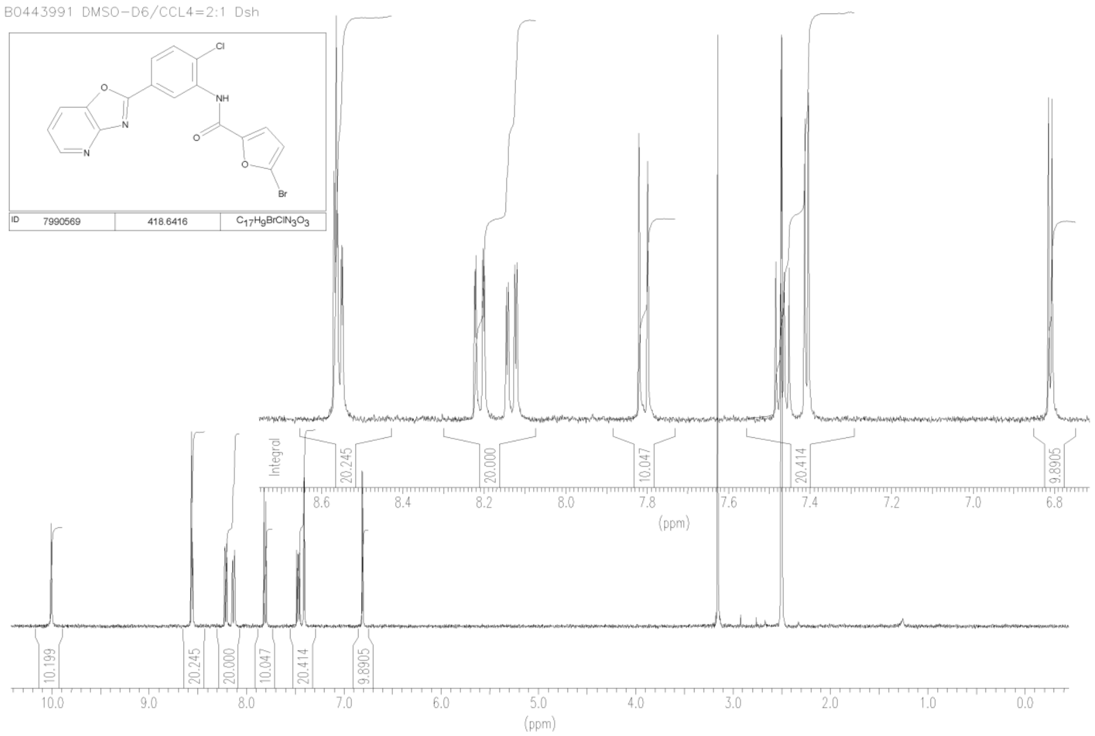

6 References

1. Taylor, P. *et al.* Ligand discovery and virtual screening using the program LIDAEUS. *Br. J. Pharmacol.* **153,** S55–67 (2008).
2. Petukhov, M. *et al.* Large-scale conformational flexibility determines the properties of AAA+ TIP49 ATPases. *Structure* **20,** 1321–31 (2012).
3. Sanders, M. P. a *et al.* Comparative analysis of pharmacophore screening tools. *J. Chem. Inf. Model.* **52,** 1607–20 (2012).
4. Pearlman, R. Concord. Tripos International; *St. Louis, Missouri, 63144, USA; Distrib. by.*
5. Hann, M. M. & Oprea, T. I. Pursuing the leadlikeness concept in pharmaceutical research. *Curr. Opin. Chem. Biol.* **8,** 255–63 (2004).
6. Sauton, N., Lagorce, D., Villoutreix, B. O. & Miteva, M. MS-DOCK: accurate multiple conformation generator and rigid docking protocol for multi-step virtual ligand screening. *BMC Bioinformatics* **9,** 184 (2008).
7. Wang, R., Lu, Y. & Wang, S. Comparative evaluation of 11 scoring functions for molecular docking. *J. Med. Chem.* **46,** 2287–303 (2003).
8. Wang, R. & Wang, S. How does consensus scoring work for virtual library screening? An idealized computer experiment. *J. Chem. Inf. Comput. Sci.* **41,** 1422–6 (2001).
9. Houston, D. R. & Walkinshaw, M. D. Consensus docking: improving the reliability of docking in a virtual screening context. *J. Chem. Inf. Model.* **53,** 384–90 (2013).
10. Neudert, G. & Klebe, G. DSX: a knowledge-based scoring function for the assessment of protein-ligand complexes. *J. Chem. Inf. Model.* **51,** 2731–45 (2011).
11. Dolinsky, T. J. *et al.* PDB2PQR: expanding and upgrading automated preparation of biomolecular structures for molecular simulations. *Nucleic Acids Res.* **35,** W522–5 (2007).
12. Li, H., Robertson, A. D. & Jensen, J. H. Very fast empirical prediction and rationalization of protein pKa values. *Proteins* **61,** 704–21 (2005).
13. O'Boyle, N. M. *et al.* Open Babel: An open chemical toolbox. *J. Cheminform.* **3,** 33 (2011).
14. Trott, O. & Olson, A. J. AutoDock Vina: improving the speed and accuracy of docking with a new scoring function, efficient optimization, and multithreading. *J. Comput. Chem.* **31,** 455–61 (2010).
15. Huey, R., Morris, G. M., Olson, A. J. & Goodsell, D. S. Software News and Update A Semiempirical Free Energy Force Field with Charge-Based Desolvation. (2007). doi:10.1002/jcc
16. Ferrins, L. *et al.* 3-(Oxazolo[4,5-b]pyridin-2-yl)anilides as a novel class of potent inhibitors for the kinetoplastid Trypanosoma brucei, the causative agent for human African trypanosomiasis. *Eur. J. Med. Chem.* **66,** 450–65 (2013).
17. Zhuravlev, F. A. Unprecedentedly mild direct Pd-catalyzed arylation of oxazolo[4,5-b]pyridine. *Tetrahedron Lett.* **47,** 2929–2932 (2006).
18. Myllymäki, M. J. & Koskinen, A. M. P. A rapid method for the preparation of 2-substituted oxazolo[4,5-b]pyridines using microwave-assisted direct condensation reactions. *Tetrahedron Lett.* **48,** 2295–2298 (2007).
19. Holler, M. G., Campo, L. F., Brandelli, A. & Stefani, V. Synthesis and spectroscopic characterisation of 2-(2'-hydroxyphenyl)benzazole isothiocyanates as new fluorescent probes for proteins. *J. Photochem. Photobiol. A Chem.* **149,** 217–225 (2002).
